# Supplementary material for: Type and Frequency in Use of Nutraceutical and Micronutrient Supplementation for the Management of Polycystic Ovary Syndrome: A Systematic Scoping Review
Source: Biomedicines. 2023 Dec 18;11(12):3349. doi: 10.3390/biomedicines11123349 (PMC10742329; doi:10.3390/biomedicines11123349)
Supplement: Supplementary file 1 [file biomedicines-11-03349-s001.zip › biomedicines-2758273-supplementary.pdf]

Supplementary Material

Table S1: Search strategy for Medline database

Search (#number)	Medline Search Terms (*Title, abstract, keyword)*
#1	PCOS OR "Polycystic Ovar*" OR PCOD OR sclerocystic OR "Stein Leventhal" OR "Polycyctic Ovar*"
#2	antioxidant* OR vitamin* OR selenium OR "folic acid" OR folate OR glutathione OR "Menevit anti-oxidant" OR carnitine OR carnitene OR "ascorbic acid" OR ascorbate OR zinc OR "fatty acid*" OR "fish oil*" OR "plant extract*" OR tocopherol OR ubiquinol OR "coenzyme Q10" OR multivitamin* OR "N-acetyl cysteine" OR "L-acetyl-carnitine" OR "acetyl L-carnitine" OR acetylcysteine OR "alpha tocopherol" OR pycnogenol OR "myo-inositol" OR inositol OR melatonin OR "omega-3*" OR "docosahexaenoic acid" OR "eicosapentaenoic acid" OR "N3 fatty acid*" OR EPA OR DHA OR "n-3*"
#3	"D-chiro-inositol" OR resveratrol OR curcumin OR cinnamon OR lipo* OR "alpha-lipoic acid" OR flaxseed* OR banaba OR berberine OR barberry OR bayberry "Gamma-aminobutyr*" OR "wine grape pomace flour" OR "grape seed extract" OR "spearmint extract" OR Bergacyn OR lactobacillus OR probiotic OR "Trazer F forte" OR monacolin* OR "red yeast rice" OR quercetin OR "thylakoid-rich spinach extract powder" OR "green tea" OR "Hibiscus sabdariffa L. powder" OR "aloe vera" OR "wild bitter melon" OR "bitter gourd" OR "berry polyphenol*" OR fenugreek OR spirulina OR hawthorn OR cardamom OR sumac
#4	thiamine OR riboflavin OR niacin OR "pantothenic acid" OR pyridoxine OR biotin OR cobalamin OR calcium OR phosphorus OR magnesium OR sodium OR chloride OR potassium OR sulfur OR sulphur OR iron OR manganese OR copper OR iodine
#5	retinaldehyde OR cholecalciferol* OR ergocalciferol* OR tocotrienol OR caroten* OR molybdenum OR cobalt OR nickel OR bromine
#6	"free radical scavenger*" OR "radical scavenger*" OR "nutritional supplement*" OR micronutrient* OR nutraceutical* OR "bioactive compound*" OR polyphenol* OR flavonoid* OR nutrient* OR mineral* OR diet* OR "trace element*"
#7	#1 AND #2
#8	#1 AND #3
#9	#1 AND #4
#10	#1 AND #5
#11	#1 AND #6
#12	#7 OR #8 OR #9 OR #10 OR #11

Table S2. Details of primary studies

Author	Aim	Country of origin	Study design	Sample	Duration	Diagnostic Criteria	ART	Supplement investigated	Findings of primary outcomes
**Agrawal et al. [1]**	Evaluate the benefit of synergetic effect of Metformin plus Myo-inositol versus Metformin alone in infertile PCOS women undergoing ovulation induction.	India	RCT	120	3 months	Rotterdam	y	Myo-inositol	The study concluded significantly higher live birth rate in women receiving the combination as compared to metformin alone.
**Nestler et al. [2]**	Investigate if D-chiro-inositol would replenish stores of the DCI-IPG mediator and improve insulin sensitivity.	Venezuela	RCT	44	6-8 weeks	Descriptive	n	Dchiro-inositol	D-chiro-inositol increases the action of insulin in patients with the polycystic ovary syndrome, thereby improving ovulatory function and decreasing serum androgen concentrations, blood pressure, and plasma triglyceride concentrations.
**Cheang et al. [3]**	Investigate whether oral DCI mediates an increase in the release of the DCI-IPG mediator and an improvement in insulin sensitivity in women with PCOS.	USA	RCT	11	6 weeks	Descriptive	n	Dchiro-inositol	The significant relationship between DCI-IPG release and insulin sensitivity suggests that the DCI-IPG mediator may be a target for therapeutic interventions in PCOS.
**Emekci Ozay et al. [4]**	Investigate the effect of myo-inositol on pregnancy rates of patients diagnosed with PCOS who have undergone controlled ovulation induction and intrauterine insemination (IUI).	Turkey	RCT	196	12 weeks	Rotterdam	y	Myo-inositol	This study shows that MYO should be considered in the treatment of infertile PCOS patients. MYO administration increases clinical pregnancy rates, lowers total rFSH dose and the duration of the ovulation induction.
**Costantino et al. [5]**	Investigate the effects of treatment with Myo-inositol on circulating insulin, glucose tolerance, ovulation and serum androgens concentrations in women with the PCOS	Italy	RCT	42	12-16 weeks	Descriptive	n	Myo-inositol plus folic acid (combined_	Treatment of PCOS patients with Myo-inositol provided a decreasing of circulating insulin and serum total testosterone as well as an improvement in metabolic factors.
**Unfer et al. [6]**	Compare the effects myo-inositol and D-chiro-inositol on oocyte quality in euglycemic PCOS patients.	Italy	RCT	84	8 weeks	Rotterdam	y	Myo-inositol, Dchiro-inositol	Data showed that, in PCOS patients having a normal insulin response, myo-inositol treatment rather than D-chiro-inositol is able to improve oocyte and embryo quality during ovarian stimulation protocols.
**Genazzani et al. [7]**	Investigate hormonal dynamics in a group of non-obese PCOS patients under myo-inositol administration.	Italy	Single arm	24	12 weeks	Rotterdam	n	Myo-inositol	MYO administration positively modulates insulin sensitivity in non-obese PCOS patients without compensatory hyperinsulinemia, improving hormonal parameters.
**De Cicco et al. [8]**	Investigate the effects of a combined treatment with alpha-lipoic acid and myoinositol on clinical, endocrine and metabolic features of women affected by PCOS	Italy	Single arm	40	6 months	Rotterdam	n	Myo-inositol, Alpha-lipoic acid	The combined treatment of ALA and MYO is able to restore the menstrual pattern and to improve the hormonal milieu of PCOS women, even in the absence of apparent changes in insulin metabolism.

**Bahadur et al. [9]**	Compare the effects of metformin alone versus combined therapy of metformin with myoinositol plus D-chiro-inositol in women with PCOS	India	RCT	72	6 months	Rotterdam	n	Myo-inositol plus Dchiro-inositol (combined)	Combined therapy with metformin and MYO plus DCI in women with PCOS and insulin resistance seems promising with the need for further studies with a greater sample size to evaluate the efficacy of this treatment.
**Prabhakar et al. [10]**	Evaluate the benefits of myoinositol plus metformin versus myoinositol alone in infertile PCOS women undergoing ovulation induction cycles.	India	RCT	116	6 months	Rotterdam	y	Myo-inositol	Myoinositol might be used alone as an insulin sensitizer to improve metabolic, hormonal and reproductive outcome in infertile PCOS women. Further studies with large numbers are warranted to confirm the role of myoinositol as a sole insulin sensitizer.
**Lesoine et al. [11]**	Investigate if the combination of myoinositol + folic acid was able to improve the oocyte quality, the ratio between follicles and retrieved oocytes, the fertilization rate, and the embryo quality in PCOS patients undergoing IVF treatments.	Germany	RCT	29	2 months	Descriptive	y	Myo-inositol	Evidence suggests that myoinositol therapy in women with PCOS results in better fertilization rates and a clear trend to a better embryo quality. As the number of retrieved oocytes was smaller in the myoinositol group, the risk of hyper stimulation syndrome can be reduced in these patients.
**Troisi et al. [12]**	The effect of inositols on the metabolomic profile of PCOS women	Italy	NRCT	30	3 months	Rotterdam	n	Myo-inositol plus Dchiro-inositol plus glucomanna (combined)	Our pilot study has identified a complex network of serum molecules that appear to be correlated with PCOS, and with a combined treatment with inositols and glucomannan.
**Nordio et al. [13]**	Evaluate the efficacy of seven different ratios between myo-inositol and Dchiro-inositol), in the therapy of PCOS	Italy	RCT	56	3 months	Rotterdam	n	Myo-inositol plus Dchiro-inositol (combined)	Our data demonstrated that DCI activity is beneficial mainly at a specific ratio with MYO, whereas the increase of DCI causes the loss of the beneficial effects at reproductive level.
**Stracquadanio et al. [14]**	Compare the clinical, endocrine, and metabolic parameters in PCOS women treated with myo-inositol, gymnemic acid, and l-methylfolate or myo- inositol and folic acid only	Italy	RCT	100	6 months	Rotterdam	n	Myo-inositol, gymnemic acid, l-methylfolate	We conclude that a good option for the treatment of PCOS is the combined administration of myo-inositol, gymnemic acid, l-methyl-folate, especially for overweight/obese patients with marked insulin resistance and with associated hyperhomocysteinemia.
**Januszewski et al. [15]**	Evaluate the effects of a combined Myo-inositol (MI) and D-chiro-inositol (DCI) therapy on the hormonal and metabolic parameters of women with PCOS.	Poland	Single arm	70	6 months	Rotterdam	n	Myo-inositol, Dchiro-inositol, folic acid, vitD, Vit B6, B5, B12	Combination of MYO and DCI in a ratio 10:1 seems to be efficient in improving both metabolic and hormonal parameters in patients with PCOS.
**Salenpour et al. [16]**	Investigate the effects of myoinositol treatment on metabolic and cardiovascular profile in PCOS women over 30 years of age	Iran	Single arm	50	3 months	Rotterdam	n	Myo-inositol plus folic acid (combined)	Results showed that supplementation with MYO and folic acid in PCOS patients over 30 years of age could decrease the risk of

									cardiovascular problems by normalising the metabolic profile.
**Ozay et al. [17]**	Examine myo-ins effects on anti-Mullerian hormone levels and compare them with those ones obtained administering COC.	Turkey	RCT	137	3 months	Rotterdam	n	Myo-inositol plus folic acid (combined)	In PCOS treatment, MYO is observed more effective in reductions of total ovarian volume and anti-Mullerian hormone levels.
**Sacchinelli et al. [18]**	Evaluate the efficacy of NAC + Inositol + folic acid on ovulation rate and menstrual regularity in PCOS patients with and without insulin resistance	Italy	NRCT	91	12 months	Rotterdam	n	Inositol plus folic acid plus N-Acetyl Cysteine (combined)	Inositol and NAC may have additional non insulin-related mechanisms of action that allow achieving benefits also in those patients with negative HOMA-index.
**Akhtar et al. [19]**	Compare the efficacy of insulin sensitizer myoinositol versus a combination of myoinositol plus metformin for ovulation induction in polycystic ovarian syndrome.	Pakistan	RCT	60	3 months	NR	n	Myo-inositol	The combination of these two drugs 'Metformin and Myoinositol', work synergistically that gives more metabolic and reproductive benefits as compared to single drug work alone.
**Carlomagno et al. [20]**	Test the synergistic effect of myo-inositol and melatonin in IVF protocols with PCOS patients	Italy	RCT	526	from day 1 of cycle to 14 days after embryo transfer	Rotterdam	y	Myo-inositol plus folic acid (combined), melatonin	Myo-inositol and melatonin have shown to enhance, synergistically, oocyte and embryo quality.
**Akbari et al. [21]**	Evaluate the effect of myo-Inositol administration on oocyte quality, fertilization rate and embryo quality in patients with PCOS during assisted reproductive technology (ART) cycles.	Iran	RCT	50	1 month before antagonist cycle til ovum pick up	Rotterdam	y	Myo-inositol plus folic acid (combined)	Findings suggest that myo-Inositol alters the gene expression in granulosa cells and improves oocyte and embryo quality among PCOS patients undergoing ART.
**Minozzi et al. [22]**	Evaluate whether the combined therapy myo-inositol plus D-chiro-inositol (in a in a physiological ratio of 40:1) improve the metabolic profile, therefore, reducing cardiovascular risk in PCOS patients.	Italy	Single arm	20	6 months	Rotterdam	n	Myo-inositol plus Dchiro-inosititol plus folic acid (combined)	The combined therapy myo- inositol plus D-chiro-inositol is able to improve the metabolic profile of PCOS women, therefore, reducing the cardiovascular risk.
**Nordio et al. [23]**	The effectiveness in reducing the risk of metabolic syndrome as well as in enhancing the ovarian functions of a combined therapy with MI and DCI was compared to a mono therapy	Italy	RCT	50	6 months	Rotterdam	n	Myo-inositol plus Dchiro-inositol plus folic acid (combined), Myo-inositol plus folic acid (combined)	The combined administration of MYO and DCI in physiological plasma ratio (40:1) should be considered as the first line approach in PCOS overweight patients, being able to reduce the metabolic and clinical alteration of PCOS and, therefore, reduce the risk of metabolic syndrome.
**Alquzwini, T. [24]**	Identify the successful outcomes of using a choline-inositol combination (CIC) in the treatment of infertility in women with PCOS.	Iraq	Single arm	60	unclear	NR	n	choline, inositol	The choline-inositol combination may introduce a new treatment era of the polycystic ovarian syndrome in women with primary and secondary infertility.

**Luorno et al. [25]**	Does the administration of D-chiro-inositol affect the concentration of circulating insulin, the levels of serum androgens, and the frequency of ovulation in lean women with the polycystic ovary syndrome.	Venezuela	RCT	20	6-8 weeks	Descriptive	n	Dchiro-inositol	We conclude that, in lean women with the polycystic ovary syndrome, D-chiro-inositol reduces circulating insulin, decreases serum androgens, and ameliorates some of the metabolic abnormalities (increased blood pressure and hypertriglyceridemia) of syndrome X.
**Ciotta et al. [26]**	Determine the effects of myo-inositol on oocyte's quality on a sample of women with polycystic ovary syndrome.	Italy	RCT	34	3 months	Descriptive	y	Myo-inositol plus folic (combined)	These data suggest that myo- inositol may be useful in the treatment of PCOS patients undergoing ovulation induction, both for its insulin-sensitising activity, and its role in oocyte maturation.
**Montanino et al. [27]**	Test a new formulation made up of MI and α-LA to successfully treat MI- resistant PCOS women.	Italy	Single arm	37	6 months	Rotterdam	n	Myo-inositol plus alpha lactalbumin (combined)	The combination of MYO with α-LA was able to re-establish ovulation, greatly increasing the chances of desired pregnancy in the treatment of PCOS MYO-resistant patients
**Fruzzetti et al. [28]**	Compare the effects of metformin and myo-inositol on the clinical and metabolic features of the women with PCOS.	Italy	RCT	50	6 months	Rotterdam	n	Myo-inositol plus folic acid (combined)	The two insulin-sensitizers, metformin and myo- inositol, show to be useful in PCOS women in lowering BMI and ameliorating insulin sensitivity, and improving menstrual cycle without significant differences between the two treatments.
**Colazingari et al. [29]**	Investigate the effects of the combined therapy myo-inositol (MI) plus D-chiro- inositol (DCI) or D-chiro-inositol treatment in oocyte quality.	Italy	RCT	100	12 weeks	Rotterdam	y	Myoinositol plus Dchiro-inositol plus folic acid (combined), Dchiro-inositol,	The present paper further supports the hypothesis that MYO plays a crucial role in the ovary in PCOS women. In particular, due to the physiological role played by MYO and DCI, the combined therapy should represent a better choice.
**Fruzzetti et al. [30]**	Evaluate the effects of the combination of D-chiro inositol and alpha lipoic acid on menstrual cycles and insulin sensitivity in women with PCOS	Italy	NRCT	72	180 days	Rotterdam	n	Dchiro-inositol, alpha lipoic acid	The association of D-chiro-inositol and alpha lipoic acid improves menstrual cycle length, restoring ovulation in the majority of women. Insulin sensitivity improved in women with IR only, confirming that in presence of IR the D-chiro-inositol has a role in restoring the insulin action overcoming the inactivity of epimerase in transforming myo-inositol to D-chiro inositol.
**Benelli et al. [31]**	Evaluate the effects of a therapy that combines myo-inositol (MI) and D-chiro-inositol (DCI) in young overweight women affected by PCOS	Italy	RCT	46	6 months	Rotterdam	n	Myoinositol plus Dchiro-inositol plus folic acid (combined)	The combined therapy of MYO plus DCI is effective in improving endocrine and metabolic parameters in young obese PCOS affected women.

**Rastegar et al. [32]**	Compare the effectiveness of metformin and myo-inositol drugs on improving reproductive outcomes of PCOS patients undergoing in vitro fertilization (IVF) treatment.	Iran	RCT	140	3 months before IVF cycle til ovum puncture	NR	y	Myo-inositol plus folic acid (combined)	There is no significant difference in the effect of metformin and myo-inositol on improving reproductive outcomes.
**Soufizadeh et al. [33]**	Determine the effect of inofolic supplementation on women with PCOS	Iran	RCT	70	3 months	NR	n	Myo-inositol plus folic acid (combined)	MYO supplementation improved fat profile status, foetal quality and reduced miscarriage and also increased follicles in women with polycystic ovary syndrome.
**Gerli et al. [34]**	Assess ovarian activity (two blood samples per week) to assess the validity of a myo-inositol therapeutic approach in this group of women.	Italy	RCT	92	14 weeks	Descriptive	n	Myo-inositol plus folic acid (combined)	These data support a beneficial effect of myo-inositol in women with oligomenorrhea and polycystic ovaries in improving ovarian function.
**Gerli et al. [35]**	Assess ovarian activity (two blood samples per week) to assess the validity of inositol in this group of women.	Italy	RCT	283	14 weeks	Descriptive	n	Inositol	These data support a beneficial effect of inositol in improving ovarian function in women with oligomenorrhea and polycystic ovaries.
**Rajasekaran et al. [36]**	Study the effects of myoinositol in comparison to metformin, in reducing the risk of OHSS and improving ART outcome in PCOS women undergoing IVF.	India	RCT	102	3 months	Rotterdam	y	Myo-inositol	Myo is equally beneficial as Met in reducing the risk of OHSS and has better ART outcome in PCOS women undergoing antagonist cycles.
**Mendoza et al. [37]**	Evaluate the effect of two doses of D-chiro-inositol (DCI) in combination with Myo-inositol (MYO) in women with PCOS undergoing ICSI.	Spain	RCT	60	12 weeks	Rotterdam	y	Myo-inositol, Dchiro-inositol	The combination of MYO-DCI at high doses of DCI improves the pregnancy rates and reduces the risk of OHSS in women with PCOS undergoing ICSI.
**Papaleo et al. [38]**	Determine the effects of myo-inositol on oocyte quality in PCOS patients undergoing intracytoplasmic sperm injection (ICSI) cycles.	Italy	RCT	60	unclear	Descriptive	y	Myo-inositol plus folic acid (combined)	These data show that in patients with PCOS, treatment with myo-inositol and folic acid, but not folic acid alone, reduces germinal vesicles and degenerated oocytes at ovum pick-up without compromising total number of retrieved oocytes.
**Mendoza et al. [39]**	Evaluate the effect of two doses of D-chiro-inositol (DCI) in combination with Myo-inositol (MYO) on the oocyte quality (OQ) of women with PCOS undergoing intracytoplasmic sperm injection (ICSI)	Spain	RCT	11	12 weeks	Rotterdam	y	Myo-inositol, Dchiro-inositol	The combination of MYO with high doses of DCI improved oocyte cytoplasm quality in women with PCOS undergoing ICSI.
**Soldat-Stankovic et al. [40]**	To compare the effects of insulin sensitizers metformin and myo-inositol on adiponectin levels and metabolic characteristics in women with PCOS with respect to their body mass index.	Bosnia and Herzegovina	RCT	66	6 months	Rotterdam	n	Myo-inositol	The two insulin sensitizers, MET and MYO, were useful in reducing BMI and improving body composition without significant differences between the two treatments in PCOS women.

**Artini et al. [41]**	Evaluate the effects the administration of myo-inositol on hormonal parameters in a group of PCOS patients.	Italy	RCT	50	12 weeks	Descriptive	y	Myo-inositol	MYO administration improves reproductive axis functioning in PCOS patients reducing the hyperinsulinemic state that affects LH secretion.
**Kitaya et al. [42]**	Evaluate the effect of myoinositol supplementation on intracytoplasmic sperm injection outcome in Japanese infertile polycystic ovarian syndrome (PCOS) women with non-obese less-androgenic phenotype	Japan	NRCT	50	1 treatment cycle of ICSI	NR	y	Myo-inositol	Myoinositol supplementation is a safe and effective treatment modality to increase the mature and fertilized oocytes, along with a reduction in the gonadotropin dose in ICSI cycles in Japanese infertile PCOS women with non-obese less-androgenic phenotype.
**Zacche et al. [43]**	Evaluate the effects of myo-inositol in the treatment of cutaneous disorders like hirsutism and acne.	Italy	Single arm	50	6 months	NR	n	Myo-inositol plus folic acid (combined)	MYO administration is a simple and safe treatment that ameliorates the metabolic profile of patients with PCOS, reducing hirsutism and acne.
**Isabella et al. [44]**	Study the role played by D-chiro-inositol at ovarian level.	Italy	RCT	54	8 weeks	Rotterdam	y	Dchiro-inositol	Increasing DCI dosage progressively worsens oocyte quality and ovarian response.
**Tagliaferri et al. [45]**	Investigate which is the more effective between metformin and myoinositol on hormonal, clinical and metabolic parameters in obese patients with PCOS.	Italy	RCT	34	6 months	Rotterdam	n	Myo-inositol	Both treatments improved the glyco-insulinaemic features of obese PCOS patients, but only metformin seems to exert a beneficial effect on the endocrine and clinical features of the syndrome.
**Tabatabaie et al. [46]**	Investigate the mechanism of Myo-Inositol, as an adjuvant, on key signalling pathways related to oocyte maturation, fertilization rate, and embryo quality as well as ovarian steroidogenesis in cumulus cells of PCOS patients	Iran	RCT	90	1 month prior to IVF until ovum pick up	NR	y	Myo-inositol plus folic acid (combined)	Our study provides new insight into the molecular mechanism underlying the positive effect of Myo-Inositol on intrinsic ovarian defects in PCOS, steroidogenesis, oocyte maturation, fertilization rate, and embryo quality.
**Piomboni et al. [47]**	Evaluate the oxidative stress status of follicular fluid and the oocyte quality in women with PCOS undergoing different ovarian stimulation protocols.	Italy	RCT	68	unclear	Rotterdam	y	Dchiro-inositol	A natural supplement and a drug both showed a statistically significant positive effect on follicular milieu by decreasing the oxidative damage on FF proteins, as well as in recovering good quality oocytes.
**Marin et al. [48]**	Evaluating the effects of myo-inositol plus alpha-lactalbumin in two groups of PCOS women, treated in Mexico and Italy.	Mexico/Italy	NRCT	34	6 months	Rotterdam	n	Myo-inositol plus alpha-lipoic acid plus folic acid (combined)	This clinical trial demonstrated for the first time that myo-inositol and alpha-lactalbumin improve important parameters in PCOS patients characterized by different metabolic profiles.
**Dona et al. [49]**	Investigate oxidative-related alterations in erythrocytes and anti-inflammatory effects of inositol in women with PCOS before and after treatment with myo-inositol (MYO).	Italy	RCT	26	12 weeks	Rotterdam	n	Myo-inositol	Treatment with MYO is effective in reducing hormonal, metabolic, and oxidative abnormalities in PCOS patients by improving IR.

**Rolland et al. [50]**	Study whether supplementation with MYO can improve patients' sensitivity to clomiphene citrate (CC) in terms of ovulation and pregnancy rates.	France	Single arm	26	unclear	Rotterdam	n	Myo-inositol, folic acid	This study proves the great interest of a RCT and re-opens the possibilities of insulin-sensitizing agents in the treatment of anovulatory patients with PCOS, such as natural products like MYO.
**Ozay et al. [51]**	To evaluate whether myoinositol and folic acid therapy has any effects on ovarian stromal blood flow in PCOS.	Turkey	NRCT	180	3 months	Rotterdam	n	Myo-inositol plus folic acid (combined)	MYO therapy reduced ovarian vascularization in both PCOS and healthy users after 3months and this decrease is especially noticeable in women with PCOS compared to healthy women. OCP therapy also reduced ovarian vascularization just like MYO therapy.
**Morgante et al. [52]**	Evaluate the role of inositol supplementation in insulin-resistant patients with PCOS, undergoing gonadotropin ovulation induction using the low-dose step-down regimen	Italy	NRCT	30	unclear	Rotterdam	n	Inositol plus lactoferrin plus bromelain (combined)	Inositol nutritional supplementation produced very good clinical results with a significant reduction in cancellation rate and the consequent improvement in clinical pregnancy rate.
**Kamenov et al. [53]**	Evaluate the effectiveness of myo-inositol alone or in combination with clomiphene citrate for (1) induction of ovulation and (2) pregnancy rate in anovulatory women with PCOS and proven insulin resistance.	Bulgaria	Single arm	50	unclear	Rotterdam	n	Myo-inositol plus folic acid (combined)	Myo-inositol treatment ameliorates insulin resistance and body weight and improves ovarian activity in PCOS patients.
**Papaleo et al. [54]**	Investigate if the administration of myo-inositol would improve the insulin-receptor activity, restoring normal ovulatory function.	Italy	Single arm	25	6 months	Descriptive	n	Myo-inositol plus folic acid (combined)	Myo-inositol is a simple and safe treatment that is capable of restoring spontaneous ovarian activity and consequently fertility in most patients with PCOS. This therapy did not cause multiple pregnancy.
**Minozzi et al. [55]**	Compare the effects of a combined contraceptive pill (OCP) in combination with myo-inositol (MI) on endocrine, metabolic, and clinical parameters in patients with PCOS	Italy	NRCT	155	12 months	Rotterdam	n	Myo-inositol plus folic acid (combined)	Our data show that a combination of combined contraceptive pill and MYO may be more effective in controlling endocrine, metabolic, and clinical profile in patients with PCOS than OCP alone and may reduce insulin levels and insulin resistance. Hence, combined treatment may become a more effective long-term therapeutic choice for controlling PCOS symptoms.
**Nehra et al. [56]**	Evaluate and compare the effect of insulin sensitizers i.e., metformin and myoinositol on anthropometric parameters in PCOS patients	India	RCT	60	24 weeks	AES	n	Myo-inositol	There was a definite improvement in anthropometric parameters with both metformin and myoinositol but on comparing these drugs, no significant difference was observed. Thus, myoinositol can also be used

									as an alternative to metformin for the treatment of PCOS.
**Nazirudeen et al. [57]**	Compare the effect of metformin monotherapy versus a combination of metformin with Myoinositol and D-chiro-inositol in PCOS.	India	RCT	65	6 months	Rotterdam	n	Myo-inositol, Dchiro-inositol	The addition of myoinositol to metformin exerts additional benefits in improving menstrual cycle regularity, and quality of life in women with PCOS.
**Isran et al. [58]**	Determine the two years' experience of myo-inositol use in women presented with PCOS	Pakistan	Single arm	65	2 years	NR	n	Myo-inositol	The treatment of females with PCOS with myoinositol has showed considerable amount of improvements in terms of increasing fertilization along with omitting menstrual cycle irregularities and weight loss of the patients.
**Vartanyan et al. [59]**	Evaluate the effectiveness of inositol in patients with PCOS for improving the quality of oocytes, the quality of embryos and the outcomes of IVF cycles	Russia	NRCT	270	unclear	NR	y	Inositol	Use of inositol in patients with PCOS during preconception care is an effective method allowing improvement of oocytes quality and positively affecting IVF cycle prognosis. High pregnancy rates per ET and 'take home baby' index after treatment are justifying inositol usage in patients with PCOS and infertility.
**Alviggi et al. [60]**	Investigate the effects of FT500 Plus® supplementation in women with PCOS undergoing controlled ovarian stimulation, and to evaluate antioxidant activity in FF collected during oocyte retrieval.	Italy	Observational	50	retrospectively evaluated - women who had undergone IVF between Jan - Dec 2014	Rotterdam	y	Myo-inositol plus folic acid plus active antioxidants (combined)	Treatment group had significantly decreased ovarian volume, increase in number of MII oocytes.
**Artini et al. [61]**	Evaluate the effect of DCI and ALA administration in PCOS patients who underwent IVF, in overweight or obese patients with a family history of diabetes	Italy	Observational	20	retrospectively evaluated women undergoing IVF from March 2018 to March 2019	Rotterdam	y	Dchiro-inositiol, alpha lipoic acid	Our results showed that combined regimen of DCI and ALA, at the low dosage we used, could be an interesting strategy in overweight PCOS patients with familial diabetes, because reproductive outcomes seem to improve after this treatment, probably through an improvement in IR.
**Regidor et al. [62]**	The aim of this study was to determine the pregnancy rates under the use of a combination of myoinositol and folic acid in patients with a PCOS in Germany, to establish if this molecule can be used as a safer treatment option for the fertility improvement of this disease.	Germany	Observational	3602	questionnaire presented to 245 gynaecologists in Germany between June 2014 and March 2015	Rotterdam		Myo-inositol plus folic acid (combined)	This confirms that myoinositol is not only an effective alternative in the treatment of PCOS patients but also a secure one as no side effects could be observed in the standard dosage. Compliance results in better outcomes in the management of ovulation, hyperandrogenism, and metabolic parameters on patients with a PCOS.

**Yaylali et al. [63]**	Compare the effects of metformin and inositol co- treatments on IVF outcomes in PCOS patients receiving IVF treatment in our centre.	Turkey	Observational	109	Patients presented at IVF centre between Jan 2016 and Jun 2017	Rotterdam	y	Myo-inositol plus folic acid (combined)	Insulin-sensitizing agents may have a positive effect on follicular development in PCOS patients applied with IVF.
**Fruzzetti et al. [64]**	Evaluate the effects of a long-term treatment with ALA plus MI on clinical and metabolic features of PCOS women.	Italy	Observational	44	retrospective analysis of completed clinical charts - follow-up visits after 6, 12, 18 and 24 months	Rotterdam	n	Myo-inositol plus alpha lipoic acid, (combined)	the combination of ALA and MYO showed to be useful as long-term therapy in PCOS women, providing a normalization of the menstrual cycle and an amelioration of insulin levels with a high tolerability.
**Wdowiak et al. [65]**	Evaluate the influence of myo-Ins on the pregnancy rate and on the dynamics of embryo development in PCOS patients undergoing ICSI.	Poland	Observational	217	3 months prior to ICSI	Rotterdam	y	Myo-inositol plus folic acid (combined)	Myoinositol increased embryo development dynamics and accelerated blastocyst stage reaching time; however, no effect was shown on clinical pregnancy.
**La Marca et al. [66]**	Analyse the effect of DCI on menstrual cycle regularity in PCOS women.	Italy	Observational	47	any point	Rotterdam	n	Dchiro-inositol	The use of DCI is associated to clinical benefits for many women affected by PCOS including the improvement in insulin resistance and menstrual cycle regularity.
**Rashad et al. [67]**	Clarify the impact of vitamin D supplementation on cardio-metabolic status, androgen profile, and clinical features of PCOS.	Egypt	RCT	145	12 weeks	Rotterdam	n	Vitamin D, calcium	The supplementation of VD for 12 weeks improved the cardio-metabolic and androgenic profiles of PCOS.
**Al-Bayyari et al. [68]**	Investigate the effect of vitamin D treatment on androgen levels and hirsutism scores in overweight women with PCOS.	Jordan	RCT	60	12 weeks	Rotterdam	n	Vitamin D	Vitamin D3 at a treatment dose of 50,000 IU per week improved 25(OH)D levels and decreased the hirsutism scores and androgen levels of overweight women with PCOS.
**Javed et al. [69]**	Explore and compare the effects of vitamin D supplementation vs. placebo on cardiovascular risk factors, hormones, and markers of liver injury and fibrosis in vitamin D overweight and obese vitamin D deficient women with PCOS.	UK	RCT	40	3 months	Rotterdam	n	Vitamin D	This study supports beneficial effects of vitamin D supplementation on liver markers and modest improvements in insulin sensitivity in vitamin D deficient women with PCOS.
**Abdulameery ahya et al. [70]**	Evaluate the effect of combining oral vitamin D3 tablet or CoQ10 capsule with clomiphene citrate on hormonal, oxidative marker, and ovulation outcome in clomiphene citrate resistance PCOS patients.	Iraq	RCT	41	2 months	Rotterdam	n	Vitamin D, co-enzyme Q10	Supplementation with vitamin D and CoQ10 to clomiphene citrate resistance PCOS patients result in improving hormonal profile, oxidative marker, and ovulation outcome.

**Fatemi et al. [71]**	Investigate the possible role of combined vitamin E and D supplementation in the ICSI outcomes of PCOS subjects.	Iran	RCT	105	8 weeks	Rotterdam	y	Vitamin D, Vitamin E	The findings of this trial do not add clinical support to the evidence that vitamins E and D3 may play a role in the success rate of IVF via an antioxidant mechanism.
**Firouzabadi et al. [72]**	Evaluate the efficacy of calcium & vitamin D supplementation in infertile women suffering from PCOS	Iran	RCT	100	6 months	Rotterdam	n	Vitamin D, calcium	This study showed the positive effects of calcium & vitamin D supplementation on weight loss, follicle maturation, menstrual regularity, and improvement of hyperandrogenism, in infertile women with PCOS.
**Kadoura et al. [73]**	This study aims to investigate the effect of combining calcium and vitamin D supplements with metformin on menstrual cycle abnormalities, gonadotropins, and IGF-1 system in vitamin D-deficient/insufficient PCOS women.	Syria	RCT	40	8 weeks	Rotterdam	n	Vitamin D, calcium	Calcium and vitamin D supplements can support metformin effect on regulation of menstrual cycle irregularity in vitamin D-deficient/insufficient PCOS patients, but this effect is not associated with any significant changes in gonadotropins or IGF-1 system.
**Lerchbaum et al. [74]**	We examined VD effects on anti-Mullerian hormone and other endocrine markers in PCOS and non- PCOS women.	Austria	RCT	330	24 weeks	Rotterdam	n	Vitamin D	In PCOS women, VD treatment for 24 weeks had a significant effect on FSH and LH/FSH ratio but no effect on AMH levels.
**Kaddoura et al. [75]**	Investigate the impact of combining calcium and vitamin D supplements with metformin on lipid profile in vitamin D deficient/insufficient PCOS women.	Syria	RCT	40	8 weeks	Rotterdam	n	Vitamin D, calcium	Adding calcium and vitamin D to metformin therapy had no superior effect on improving lipid profile in vitamin D deficient/insufficient subjects with PCOS.
**Seyyed et al. [76]**	Evaluate the effect of vitamin D supplementation on insulin resistance, visceral fat, and adiponectin in hypovitaminosis D women with PCOS	Iran	RCT	44	8 weeks	Rotterdam	n	Vitamin D	Vitamin D supplementation in vitamin D deficient women with PCOS, improved the FPG, HOMA-B, Adiponectin, and serum vitamin D level.
**Rahimi-Ardabili et al. [77]**	To investigate the effect of cholecalciferol on CVD risk factors in PCOS women with vitamin D deficiency.	Iran	RCT	50	2 months	Rotterdam	n	Vitamin D	this study showed that vitamin D3 therapy had beneficial effects on some CVD risk factors in PCOS patients with vitamin D deficiency.
**Wehr et al. [78]**	Examine the effect of vitamin D supplementation on metabolic and endocrine parameters in PCOS women.	Austria	Single arm	57	24 weeks	Rotterdam	n	Vitamin D	These results suggest that vitamin D treatment might improve glucose metabolism and menstrual frequency in PCOS women.
**Rasheedy et al. [79]**	Assess the effect of vitamin D supplementation on ovulation rate in overweight subfertile women with PCOS undergoing ovulation induction.	Egypt	RCT	186	4 cycles	Rotterdam	n	Vitamin D, calcium	In subfertile women with PCOS undergoing induction of ovulation, vitamin D supplementation significantly improved the ovulation rate; however, there was no effect on clinical or biochemical pregnancy.
**Asadi et al. [80]**	Determine whether administration of vitamin D affects the success rates of intra uterine insemination in infertile PCOS women	Iran	RCT	110	2 months	Rotterdam	y	Vitamin D	It seems that administration of vitamin D induces endometrial proliferation in PCOS women during IUI cycle.

**Jafari-Sfidvajani et al. [81]**	Evaluate the effects of vitamin D supplementation in combination with low-calorie diet on anthropometric indices, reproductive hormones and menstrual regularity in overweight and obese PCOS women.	Iran	RCT	60	12 weeks	Rotterdam	n	Vitamin D	In women with PCOS, androgen profile did not change with vitamin D supplementation when combined with low-calorie diet, but menstrual frequency significantly improved.
**Rashidi et al. [82]**	Investigate the effects of vitamin D on metabolic disorders in women with PCOS and vitamin D deficiency.	Iran	RCT	86	2 months	Rotterdam	n	Vitamin D, calcium	This study showed that vitamin D replacement in women with PCOS and vitamin D deficiency has no effect on the improvement of metabolic parameters and HOMA-IR.
**Rashidi et al. [83]**	Evaluate the effects of calcium-vitamin D and metformin on the menstrual cycle and ovulation in patients with PCOS	Iran	RCT	60	3 months	Rotterdam	n	Vitamin D, Calcium	The effects of metformin and calcium-vitamin D in regulating the menstrual cycle suggest that they could also be effective for the treatment of anovulation and oligomenorrhea, with possible consequences for pregnancy rates in PCOS patients.
**Al-Thuwaynee et al. [84]**	Evaluate the effect of Vitamin D supplementation on insulin sensitivity and androgen level in Iraqi females with PCOS.	Iraq	Single arm	60	2 months	Rotterdam	n	Vitamin D	Women with PCOS have a statically significant low level of Vitamin D in mean, IGT test in the mean, and high level of testosterone, and there were inverse correlations between Vitamin D with IGT and testosterone.
**Karadag et al. [85]**	Identify the effects of vitamin D supplementation on insulin sensitivity and androgen levels in vitamin-D-deficient PCOS patients.	Turkey	NRCT	121	12 weeks	Rotterdam	n	Vitamin D	Vitamin D supplementation increased insulin sensitivity and decreased androgen levels in vitamin-D-deficient women with PCOS but did not have any effect in vitamin-D-deficient non-PCOS women.
**Ghazi et al. [86]**	Evaluate the association of vitamin D deficiency with sex hormones and metabolic markers.	Iraq	NRCT	80	6 months	Rotterdam	n	Vitamin D	Vitamin D plays a role in the improvement of biochemical variables like serum insulin level, fasting blood sugar and Homeostasis model assessment of insulin resistance in both obese and nonobese polycystic ovarian Iraqi women.
**Salehpour et al. [87]**	Explore the effect of vitamin D supplementation on insulin resistance in a group of Iranian patients with PCOS and vitamin D deficiency	Iran	Single arm	41	2 months	Rotterdam	n	Vitamin D	A single injection of vitamin D significantly decreased serum insulin levels and insulin resistance among patients with polycystic ovary syndrome.
**Bonakdaran et al. [88]**	Determine the effects of vitamin D treatment in metabolic components and ovulation evidence in PCOS.	Iran	RCT	51	3 months	Rotterdam	n	Vitamin D	Calcitriol treatment in PCOS may be beneficial prior to metformin in ovulation induction.
**Irani et al. [89]**	Determine the effect of VD supplementation on TGF-◎1 bioavailability in VD-deficient	USA	RCT	45	4 months	Rotterdam	n	Vitamin D	VD supplementation in VD-deficient women with PCOS significantly decreases the

	women with PCOS and assess whether changes in TGF-◎1/soluble endoglin (sENG) levels correlate with an improvement in PCOS clinical manifestations.								bioavailability of TGF-β1, which correlates with an improvement in some abnormal clinical parameters associated with PCOS.
**Raja-Khan et al. [90]**	To determine the effects of high-dose vitamin D on insulin sensitivity in PCOS	USA	RCT	28	12 weeks	NIH	n	Vitamin D	In women with PCOS, insulin sensitivity was unchanged with high-dose vitamin D but there was a trend towards decreased 2-hour insulin and a protective effect on blood pressure.
**Trummer et al. [91]**	Investigate the effects of vitamin D supplementation on plasma glucose and on other metabolic and endocrine parameters	Austria	RCT	180	24 weeks	Rotterdam	n	Vitamin D	Vitamin D supplementation had no significant effect on metabolic and endocrine parameters in PCOS with the exception of a reduced plasma glucose during OGTT.
**Ardabili et al. [92]**	Investigate if vitamin D supplementation would lower the glucose level and insulin resistance in women with PCOS and a vitamin D deficiency.	Iran	RCT	50	2 months	Rotterdam	n	Vitamin D	The study did not demonstrate the effect of vitamin D supplementation on insulin sensitivity and insulin resistance in women with PCOS and vitamin D deficiency.
**Al-Mosawi et al. [93]**	Investigate the association between vitamin D3 deficiency and ovulation in PCOS women patients	Iraq	NRCT	66	3 months	NR	n	Vitamin D	The important finding was that vitamin D3 might enhance follicular maturation in PCOS patients.
**Figurova et al. [94]**	Evaluate the effect of vitamin D supplementation in obese, insulin-resistant and vitamin D-deficient PCOS women on metabolic abnormalities in comparison to the effect of metformin or combined metformin plus vitamin D therapy.	Slovakia	RCT	39	6 months	AES	n	Vitamin D	We conclude that vitamin D supplementation has no significant effect on anthropometric and metabolic parameters in PCOS women.
**Dravecka et al. [95]**	Evaluate the effect of vitamin D supplementation in obese, insulin resistant and vitamin D deficient PCOS women on biochemical and clinical hyperandrogenism and menstrual irregularity in comparison to effect of metformin or combined metformin plus vitamin D therapy	Slovakia	RCT	39	6 months	Androgen excess society criteria	n	Vitamin D	Vitamin D administration has no significant effect on androgen levels and clinical features of hyperandrogenism in obese vitamin D deficient PCOS women. However, it can potentiate effect of metformin on testosterone levels and LH/FSH ratio but not on clinical hyperandrogenism and pregnancy rate.
**Pal et al. [96]**	Assess effects of vitamin D and Calcium on hormonal and metabolic milieu of PCOS	USA	Single arm	12	3 months	Descriptive	n	Vitamin D, calcium	Androgen and BP profiles improved followed three-month intervention, suggesting therapeutic implications of vitamin D and Ca in overweight and vitamin D deficient women with PCOS.
**Sert et al. [97]**	Investigated the effect of calcium and vitamin D (Ca/Vit D) supplementation on the clinical, hormonal, and metabolic profile of patients with low vitamin D levels.	Turkey	Single arm	75	8 weeks	Rotterdam	n	Vitamin D, calcium	Ca/Vit D supplementation can improve PCOS symptoms such as menstrual dysfunction, hirsutism, and hyperandrogenism.

**Garg et al. [98]**	To assess the effect of vitamin D supplementation on parameters of insulin sensitivity/ resistance and insulin secretion in subjects with PCOS	India	RCT	36	6 months	Rotterdam	n	Vitamin D	Supplementation of vitamin D, at a dose of 4000 IU/day for 6 months, did not have any significant effect on parameters of IS/IR and insulin secretion in subjects with PCOS.
**Selimoglu et al. [99]**	Determine the effect of vitamin D replacement therapy on glucose metabolism, insulin, and androgen levels in obese, insulin-resistant women with PCOS.	Turkey	Single arm	11	3 weeks	Descriptive	n	Vitamin D	Women with PCOS have mostly insufficient vitamin D levels, and vitamin D replacement therapy may have a beneficial effect on IR in obese women with PCOS.
**Tehrani et al. [100]**	Evaluating the efficacy of calcium and Vitamin D supplementation on the regularity of menstrual cycles, body mass index and hyper androgenism state of women with PCOS.	Iran	RCT	80	4 months	NIH	n	Vitamin D, calcium	Vitamin D and calcium supplementation in addition to metformin therapy in women with PCOS could result in a better outcome in a variety of PCOS symptoms including menstrual regularity, and ovulation.
**Kotsa et al. [101]**	Determine the effect of treatment with vitamin D3 analogue in the parameters of glucose metabolism in obese women with PCOS	Greece	Single arm	15	3 months	Rotterdam	n	Vitamin D	Treatment with the vitamin D3 analogue (alphacalcidol) could be of value in the management of PCOS.
**Zhuang et al. [102]**	Explore the clinical efficacy of vitamin D combined with metformin and clomiphene in the treatment of patients with PCOS combined with infertility.	China	Observational	396	retrospective analysis clinical data	NR	n	Vitamin D	The clinical efficacy of VD combined with MF and CC in the treatment of infertility patients with polycystic ovary syndrome is better than that of MF and CC, which has reference significance for the clinical treatment of PCOS infertility.
**Kudugunti et al. [103]**	Study the effect of vitamin D supplementation on menstrual cycle dysfunction and metabolic parameters in PCOS women	India	Observational	75	retrospective analysis of clinical data from baseline and 6month follow-up	Rotterdam	n	Vitamin D	vitamin D supplementation was not associated with menstrual regularization, but weight loss is associated with regularization of menstrual cycles and improvement in metabolic parameters and vitamin D supplementation may play a role in synergy with weight loss measures.
**Kumar et al. [104]**	Evaluate and compare the efficacy of metformin and N-acetyl cysteine (NAC) on clinical, hormonal, and fertility aspects in PCOS	India	RCT	100	12 weeks	NR	n	N-acetyl cysteine	The present study concludes that NAC found to improve some clinical features, biochemical markers of insulin resistance and hormonal levels and long-term health of women with PCOS through improvement in peripheral insulin and fewer side effects.
**Chandil et al. [105]**	Compare of metformin and N acetylcysteine on clinical, metabolic parameter and hormonal profile in women with PCOS	India	RCT	100	24 weeks	Rotterdam	n	N-acetyl cysteine	Better improvement of metabolic and hormonal profile was observed in the N acetylcysteine group
**Badawy et al. [106]**	Compare clomiphene citrate plus N-acetyl cysteine versus clomiphene citrate for	Egypt	RCT	573	2 cycles	Descriptive	n	N-acetyl cysteine	N-Acetyl cysteine is proved effective in inducing or augmenting ovulation in polycystic ovary patients.

	inducing ovulation in patients with polycystic ovary syndrome.								
**Elnashar et al. [107]**	Compare the effect of N-acetyl cysteine and metformin on hormonal profile and ovulation rate in women with clomiphene citrate–resistant PCOS	Egypt	RCT	61	6 weeks	Rotterdam	n	N-acetyl cysteine	Metformin alone is an effective drug in inducing ovulation in clomiphene citrate resistant polycystic ovary syndrome, whereas N-acetyl cysteine alone is not.
**Cheraghi et al. [108]**	Investigate the effects of metformin, N-acetylcysteine and their combination on follicular fluid parameters, oocytes and embryo quality in PCOS patients.	Iran	RCT	60	6 weeks	Rotterdam	y	N-acetyl cysteine	This study concluded that NAC improves oocyte and embryo quality and could be administered as an alternative to MET.
**Cheraghi et al. [109]**	Compare the effects of MTF and NAC combination on serum metabolite and hormonal levels during the course of ovulation induction in PCOS individual candidates of intracy- toplasmic sperm injection (ICSI).	Iran	RCT	80	day 3 of previous cycle to ovum pickup	Rotterdam	y	N-acetyl cysteine	Considering the adverse effect of combination therapy, we proposed the coadministration might have no beneficial effect for PCOS patient during course of ovulation induction of ICSI
**Nemati et al. [110]**	Evaluate the effects of short- and long-term treatment with metformin and NAC, in an adjuvant to clomiphene citrate (CC), on the improvement of hormonal profile and fertility status in CC-resistant women with PCOS.	Iran	RCT	108	12 weeks	Rotterdam	y	N-acetyl cysteine	Compared with metformin, administration of NAC in an adjuvant to CC is recommended for improving of hormonal profile and treatment of anovulatory infertility in hyperinsulinemic patients especially women with PCOS who are CC-resistant.
**Masha et al. [111]**	Study the effects of a prolonged treatment with NAC and ARG in combination in patients with PCOS, focusing on their ovarian function as well as on some metabolic parameters.	Italy	Single arm	8	6 months	AES	n	N-acetyl cysteine, l-arginine	This preliminary, open study suggests that prolonged treatment with NAC+ARG might restore gonadal function in PCOS. This effect seems associated to an improvement in insulin sensitivity.
**Waseem et al. [112]**	Compare the frequency of ovulation with clomiphene citrate plus N-acetyl cysteine versus clomiphene citrate alone in PCOS	Pakistan	RCT	60	3 months	NR	n	N-acetyl cysteine	This study could not find any clinical superiority for clomiphene citrate plus N-acetyl cysteine versus clomiphene citrate alone in term of ovulation rate.
**Teimouri et al. [113]**	Compare the effect of letrozole alone with letrozole plus N-acetyl cysteine (NAC) on the pregnancy rate in patients with PCOS.	Iran	RCT	317	12 days	Rotterdam	n	N-acetyl cysteine	The results of this search revealed that the number of follicles with the size of 18 mm in the control group was higher compared to the intervention group, but the fertility rate in patients with PCOS receiving letrozole plus N-acetylcysteine was significantly higher.
**Kilic-Okman et al. [114]**	Investigate the effects of NAC administration on insulin resistance, the blood hormone and lipid profile, and homocysteine levels in women with PCOS	Turkey	Single arm	20	4 weeks	Not reported	n	N-acetyl cysteine	N-acetyl-cysteine appears to be effective as an insulin and testosterone-lowering drug in women with polycystic ovary syndrome. Furthermore, we show that NAC may also be

									used as a therapeutic agent to ameliorate the homocysteine and lipid profile in PCOS.
**Lak et al. [115]**	To evaluate the effect of NAC on pregnancy rate in PCOS patients who were candidates for intrauterine insemination	Iran	RCT	100	1 cycle	Rotterdam	y	N-acetyl cysteine	NAC is ineffective in inducing or augmenting ovulation in PCOS patients who were candidates for intrauterine insemination and cannot be recommended as an adjuvant to CC in such patients.
**Mostajeran et al. [116]**	Evaluate the influence of oral N-acetylcysteine (NAC) application as an adjuvant to letrozole on induced ovulation outcomes in patients with polycystic ovary syndrome (PCOS).	Iran	RCT	130	1 cycle including 6th week of gestation	Rotterdam	n	N-acetyl cysteine	NAC is demonstrated to be a safe and well-tolerated adjuvant to letrozole and can increase the pregnancy rates in PCOS patients.
**Salehpour et al. [117]**	Evaluate the effect of oral N-acetylcysteine administration as an adjuvant to clomiphene citrate on induction of ovulation outcomes in patients with PCOS	Iran	RCT	180	1 cycle including 6th week of gestation	Rotterdam	n	N-acetyl cysteine	NAC as a safe and well-tolerated adjuvant to CC for induction of ovulation can improve the ovulation and pregnancy rates in PCOS patients.
**Maged et al. [118]**	Assess the adjuvant effect of metformin and N-acetylcysteine to clomiphene citrate in induction of ovulation in PCOS patients	Egypt	RCT	120	3 cycles	Rotterdam	n	N-acetyl cysteine	NAC as an adjuvant to CC for induction of ovulation improves ovulation and pregnancy rates in PCOS patients with beneficial impacts on endometrial thickness.
**Salehpour et al. [119]**	Evaluate the effects of NAC on manifestations of PCOS as well as improvement of fertility status.	Iran	RCT	46	6 weeks	Rotterdam	n	N-acetyl cysteine	N-Acetyl Cysteine improves lipid profile, hormonal levels, ovulation status, and long-term health of women with PCOS with limited adverse effects.
**Gupta et al. [120]**	Compare the effects of N-acetyl cysteine, metformin and vitamin D3 with calcium on clinical and metabolic profile in PCOS.	India	RCT	66	3 months	Rotterdam	n	N-acetyl cysteine, vitamin D, calcium	N-acetyl cysteine had better improvement in clinical, and metabolic profile than metformin and vitamin D3 with Calcium group in PCOS patients.
**Cheraghi et al. [121]**	Investigate the effects of metformin (MET), N-acetylcysteine (NAC) and their combination on the hormonal levels and expression profile of GDF-9, BMP-15 and c-kit, as hallmarks of oocyte quality, in PCOS patients.	Iran	RCT	80	6 weeks	Rotterdam	y	N-acetyl cysteine	This study concluded that NAC can improve the quality of oocytes in PCOS.
**Javanmanesh et al. [122]**	Compare N-acetyl cysteine and metformin on polycystic ovary syndrome (PCOS).	Iran	RCT	120	24 weeks	Rotterdam	n	N-acetyl cysteine	NAC can improve lipid profile and fasting blood sugar (FBS) and fasting blood insulin better than metformin.
**Nasr et al. [123]**	Evaluate N-acetyl-cysteine as an adjunctive therapy following unilateral laparoscopic ovarian drilling for clomiphene citrate-resistant women with PCOS	Egypt	RCT	60	12 cycles	Rotterdam	n	N-acetyl cysteine	In conclusion, NAC is a novel adjuvant therapy after unilateral LOD which might help improve overall reproductive outcome.

**Fulghesu et al. [124]**	Evaluate the effect of N-acetyl-cysteine on insulin secretion and peripheral insulin resistance in subjects with PCOS	Italy	NRCT	37	5-6 weeks	Descriptive	n	N-acetyl cysteine	NAC may be a new treatment for the improvement of insulin circulating levels and insulin sensitivity in hyperinsulinemic patients with polycystic ovary syndrome.
**Rizk et al. [125]**	Evaluate the effect of N-acetyl-cysteine as an adjuvant therapy in subjects with PCOS resistant to clomiphene citrate	Egypt	RCT	150	1 cycle plus 12 weeks	Descriptive	n	N-acetyl cysteine	The NAC as an adjuvant to CC was more effective than placebo for CC-resistant patients with PCOS. It is safe and well tolerated
**Vine et al. [126]**	Determine the effect of 12 weeks of high dose fish oil and metformin therapy on fasting and nonfasting plasma lipids and ApoB-remnants in young women with the metabolic syndrome (MetS) and PCOS.	Canada	RCT	29	12 weeks	Rotterdam	n	Fish oil	The findings of this pilot trial show that high dose FO and FO-metformin combination therapy tend to lower fasting and postprandial plasma TG and ApoB- lipoprotein remnants compared with metformin; however, the study is limited by small sample size.
**Cussons et al. [127]**	Examine the effects of omega-3 fatty acids on liver fat in PCOS.	Australia	RCT	25	8 weeks	Descriptive	n	Fish oil	Omega-3 fatty acid supplementation has a beneficial effect on liver fat content and other cardiovascular risk factors in women with PCOS, including those with hepatic steatosis.
**Vargas et al. [128]**	Compare the effects of essential vs long-chain omega (n)-3 polyunsaturated fatty acids in PCOS	USA	RCT	67	6 weeks	NIH	n	Fish oil, Flaxseed, Soybean oil	Long- chain vs essential n-3 PUFA–rich oils have distinct metabolic and endocrine effects in polycystic ovary syndrome; and therefore, they should not be used interchangeably.
**Phelan et al. [129]**	Determine the associations between plasma PUFAs and metabolic and hormonal aspects of PCOS to investigate the efficacy of LC n23 PUFA supplementation	Ireland	RCT	22	6 weeks	NIH	n	Fish oil	Cross-sectional data suggest that PUFAs modulated hormonal and lipid profiles and that supplementation with LC n23 PUFAs improves androgenic profiles in PCOS.
**Rafraf et al. [130]**	Investigate the effects of omega-3 fatty acids on obesity status, insulin resistance, and serum levels of visfatin in PCOS patients.	Iran	RCT	61	8 weeks	Rotterdam	n	Fish oil	Omega-3 fatty acids improved insulin sensitivity in PCOS patients. This beneficial effect was not associated with alteration in anthropometric measurements and serum visfatin levels.
**Vaziri et al. [131]**	Determine the effect of omega-3 supplementation on sex hormone-binding protein, testosterone, free androgen index and menstrual status in women with PCOS.	Iran	RCT	78	8 weeks	Rotterdam	n	Omega-3	Omega-3 supplementation could reduce serum concentrations of testosterone and regulate menstrual cycle without significant effect on SHBG and FAI.
**Trop-Steinberg et al. [132]**	Evaluate the effect of omega 3 during ovulation induction using clomiphene	Israel	RCT	34	60 treatment cycles	NR	n	Fish oil	Omega 3 treatment V placebo significantly improved the clinical pregnancy rate in overweight/obese women with PCOS
**Nadjarzadeh et al. [133]**	Determine the effect of omega-3 supplementation on visfatin, adiponectin, and anthropometric indices in PCOS women.	Iran	RCT	84	8 weeks	R	n	Fish oil	Our results showed that 8 weeks of supplementation of omega-3 may have some beneficial effects on PCOS biochemical

									characteristics such as LH, LH/FSH, and adiponectin.
**Karakas Se Perroud et al. [134]**	Investigate the effects of fish, flaxseed and soybean oils on BCAA and other primary metabolites in PCOS.	USA	RCT	67	6 weeks	NIH	n	Fish oil, Flaxseed, Soybean oil	As compared to Flaxseed, Fish oil and Soybean oil increased insulin response and resistance as well as several BCAA and aromatic AA.
**Benharrat et al. [135]**	Evaluate the effect of Omega 3 on the improvement of some metabolic parameters.	Algeria	RCT	60	6 weeks	Rotterdam	n	Fish oil	Omega 3 supplementation improves hyperglycemia, inflammation, and antioxidant defence in may lead to decreased cardiovascular complications.
**Mohammadi et al. [136]**	Investigate the effects of omega-3 fatty acids on serum paraoxonase 1 activity and lipids ratios in PCOS	Iran	RCT	64	8 weeks	Rotterdam	n	Fish oil	Omega-3 fatty acids may decrease the risk for cardiovascular disease through the improvement in paraxonase-1 activity and reduction in some lipid ratio in PCOS women.
**Khani et al. [137]**	Evaluate effectiveness of omega-3 supplementation on metabolic syndrome and symptoms in women with PCOS.	Iran	RCT	88	6 months	NIH	n	Fish oil	Omega-3 decrease lipid profiles, WC, and interval between periods while weight, hip circumference, fasting blood sugar, number of ovarian follicle, size of ovary, bleeding volume, menstrual bleeding, and hirsutism score did not change by administration of omega-3.
**Mohammadi et al. [138]**	Investigate the effects of omega−3 fatty acids on serum adiponectin levels and some metabolic risk factors in PCOS patients.	Iran	RCT	64	8 weeks	Rotterdam	n	Fish oil	Omega−3 fatty acids had some beneficial effects on serum adiponectin levels, insulin resistance and lipid profile in PCOS patients and may contribute to the improvement of metabolic complications in these patients.
**Farzana et al. [139]**	Assess the effects of flaxseed powder on ovarian morphology, menstrual cycle, hirsutism and blood sugar in PCOS	India	Single arm	32	3 months	Rotterdam	n	Flaxseed	Flaxseed supplementation has caused significant reduction in ovarian volume and number of follicles in polycystic ovaries, improved the menstrual cycles and not altered the body weight, blood sugar and hirsutism.
**Genazzani et al. [140]**	To evaluate the efficacy of alpha-lipoic acid administration on hormonal and metabolic parameters of obese PCOS patients.	Italy	Single arm	32	12 weeks	Rotterdam	n	Alpha-lipoic acid	ALA integrative administration at a low dosage of 400 mg daily improved the metabolic impairment of all PCOS patients especially in those PCOS with familiar diabetes who have a higher grade of risk of NAFLD and predisposition to diabetes.
**Masharani et al. [141]**	Determine whether a preparation of controlled-release alpha lipoic acid influences features of the PCOS	USA	Single arm	6	16 weeks	Rotterdam	n	Alpha-lipoic acid	These data suggest that the CRLA has positive effects on the PCOS phenotype. The effects of CRLA, however, may have been exerted through a mechanism not involving changes in oxidative stress.

**Karimi et al. [142]**	Evaluate the effects of synbiotics supplementation on lipid and anthropometric profiles in infertile women with PCOS.	Iran	RCT	99	12 weeks	Rotterdam	n	Synbiotic (7 strains bacteria)	Overall, 12 weeks of synbiotics supplementation among PCOS women resulted in beneficial effects on LDL and HDL, although it is not yet clear how much our findings are clinically significant and more clinical studies with larger sample sizes are still needed.
**Zhang et al. [143]**	Monitor the impact of probiotic supplementation on the intestinal microbiome of PCOS patients	China	Single arm	14	10 weeks	Descriptive	n	Bifidobacterium lactis V9	Notably, we observed that the PCOS-related clinical indices and the intestinal microbiotas of the participating patients exhibited an inconsistent response to the intake of the B. lactis V9 probiotic.
**Ghanei et al. [144]**	Evaluate the effects of probiotic supplement on clinical and immunological parameters of PCOS.	Iran	RCT	60	12 weeks	Rotterdam	n	4 strains of lactobacillus	This study observed that Lactobacillus supplementation modulates inflammation in PCOS patients.
**Karimi et al. [145]**	Investigate the effect of synbiotics on metabolic parameters and apelin in PCOS patients.	Iran	RCT	88	12 weeks	Rotterdam	n	Synbiotic (7 strains of bacteria)	A 12-week synbiotic supplementation has no significant beneficial effects on HOMA-IR and CRP in PCOS patients, whereas the level of apelin 36 significantly decreased.
**Darvishi et al. [146]**	Investigated the symbiotic supplement influences on serum glycaemic indices and lipids as well as apelin rates and obesity values in PCOS patients.	Iran	RCT	68	8 weeks	Rotterdam	n	Synbiotic (7 strains of bacteria)	Synbiotic supplementation improved glycaemic indices, lipid profile and obesity values in women with PCOS.
**Shamasbi et al. [147]**	Determine the effect of prebiotics on anthropometric indices in patients with PCOS	Iran	RCT	62	6 months	Rotterdam	n	Prebiotic	This study confirmed the positive and significant effects of prebiotics in reducing anthropometric indices, three and six months after the intervention in women with PCOS.
**Chudzicka-Strugala et al. [148]**	Evaluate effects of lifestyle modifications and synbiotic supplementation on PCOS.	Poland	RCT	65	3 months	Rotterdam	n	Synbiotic (2 strains of bifidobacterium lactis, lactobacillus acidophilus, lactobacillus paracasei, lactobacillus plantarum, lactobacillus salivarius, lactobacillus lactis, prebiotics fructooligosaccharides and inulin	Synbiotic supplementation potentiated effects of lifestyle modifications on weight loss and led to significant reduction of serum testosterone.

**Shamasbi et al. [149]**	Determine the effect of resistant Dextrin on metabolic parameters and high sensitivity C-reactive protein, and androgen levels	Iran	RCT	62	3 months	Rotterdam	n	Prebiotic	Resistant dextrin consumption can regulate metabolic parameters and androgen levels and manifestations including hirsutism and menstrual cycle irregularity in women with PCOS.
**Lagowska et al. [150]**	Evaluate if changes in faecal short-chain fatty acids content with an energy-restricted diet and with/ without 12 × 109 CFU/day of Lactobacillus rhamnosus supplementation affect the abundance of selected gut bacteria and lipid profile in overweight and obese women with PCOS.	Poland	RCT	40	20 weeks	Rotterdam	n	Lactobacillus rhammosus	Twenty weeks of probiotic supplementation has no additional beneficial effects on selected gut bacteria abundance, SC- FA levels, or lipid profile beyond the effect of an energy-restricted diet in overweight and obese women with PCOS.
**Kaur et al. [151]**	Ascertain the impact of multi-strain probiotic along with dietary and lifestyle modifications on restoration of menstrual regularity, weight reduction, metabolic and hormonal profile in women diagnosed with PCOS.	India	RCT	104	6 months	Rotterdam	n	Probiotic (multi-strain)	Multi-strain probiotic along with dietary and lifestyle modifications were effective in the management of PCOS.
**Talaat et al. [152]**	Evaluate the added value of cinnamon to metformin in controlling symptoms of PCOS	Saudi Arabia	RCT	233	6 months	Rotterdam	n	Cinnamon	Cinnamomum Zeylanicum supplement in a daily dose of 336 mg constitutes an added value to metformin in weight reduction, redistribution of body fat and restoring cycle regularity.
**Kort et al. [153]**	Determine the effect of cinnamon on menstrual cyclicity and metabolic dysfunction in women with PCOS	USA	RCT	45	6 months	Rotterdam	n	Cinnamon	These preliminary data suggest that cinnamon supplementation improves menstrual cyclicity and may be an effective treatment option for some women with PCOS.
**Wiweko et al. [154]**	To determine the effect of metformin and DLBS3233 on serum AMH level.	Indonesia	RCT	51	6 months	Rotterdam	n	Cinnamon	There was a significant decrease in the serum AMH level after administration of either metformin or Cinnamon supplement.
**Mika et al. [155]**	Investigate the effects of cinnamon supplementation on glycaemic indices, serum lipids and adiponectin in patients with PCOS	Iran	RCT	84	8 weeks	Rotterdam	n	Cinnamon	Short term supplementation of cinnamon had some favourable effects on metabolic risk factors of women with PCOS and may be useful in management of PCOS complications.
**Borzoei et al. [156]**	Investigate the effects of cinnamon supplementation on antioxidant status and serum lipids in women with PCOS	Iran	RCT	84	8 weeks	Rotterdam	n	Cinnamon	Cinnamon supplementation improved antioxidant status and serum lipid profile in women with PCOS and may be applicable for reducing PCOS risk factors.
**Hajimonfarednejad et al. [157]**	Our aim is to assess the effect of cinnamon powder capsules on insulin resistance, anthropometric measurements, glucose and	Iran	RCT	66	12 weeks	Rotterdam	n	Cinnamon	The present results suggest that complementary supplementation of cinnamon significantly reduced fasting insulin and insulin resistance in women with PCOS.

	lipid profiles, and androgens of women with PCOS								
**Khan et al. [158]**	Evaluate the effect of darchini (cinnamon) in the management of PCOS.	India	RCT	40	60 days	Rotterdam	n	Cinnamon	Our results support the fact that, despite the small metabolic and hormonal changes, cinnamon therapy is well tolerated by the majority of patients and may be clinically useful, especially in patients with menstrual disturbances in PCOS.
**Wang et al. [159]**	Test that oral cinnamon extract would improve insulin sensitivity in women with PCOS.	USA	RCT	15	8 weeks	NR	n	Cinnamon	Comparisons of post-treatment to baseline insulin sensitivity indices using fasting and 2-hour oral glucose tolerance tests showed significant reductions in insulin resistance in the cinnamon group but not in the placebo group.
**Permadi et al. [160]**	Investigate the effect of DLBS3233 on lipid profile, insulin resistance, and free testosterone of women with PCOS with high BMI.	Indonesia	Single arm	66	6 months	Rotterdam	n	Cinnamon	This study showed that a cinnamon supplement holds promise as a novel therapy to improve lipid profile for women with PCOS.
**Kazemi et al. [161]**	Report on the antioxidant properties of Ellagic acid for its evaluation on the Insulin resistance, oxidative stress and sex hormones levels in women with PCOS	Iran	RCT	60	8 weeks	Rotterdam	n	Ellagic acid	Ellagic acid supplementation can be helpful as a diet supplement in women with PCOS through improvement in insulin resistance.
**Gharaei et al. [162]**	Investigate the effect of astaxanthin on the oxidative stress response and assisted reproductive technology outcomes in PCOS patients.	Iran	RCT	42	40 days	Rotterdam	y	Astaxanthin	Astaxanthin treatment has been shown to increase both serum TAC levels and activation of the Nrf2 axis in PCOS patients' GCs.
**Arentz et al. [163]**	Compare the effectiveness and safety of a lifestyle intervention plus herbal medicine against lifestyle alone in overweight women with PCOS	Australia	RCT	122	3 months	Rotterdam	n	Extract of Glycyrrhiza glabra, Paeonia lactiflora, Cinnamomum verum and Hypericum perforatum (combined), Tribulus terrestris extract	This trial provides evidence of improved effectiveness and safety for lifestyle intervention when combined with herbal medicines in women with PCOS
**Ainehchi et al. [164]**	Evaluate the effect of a herbal mixture alone and in combination with clomiphene citrate (CC) compared to CC on the treatment of PCOS	Iran	RCT	60	3 months	Rotterdam	n	Mentha spicata, Zingiber officinale, Cinnamomum zeylanicum and Citrus sinensis (combined)	In general, the herbal mixture along with CC was found to improve free testosterone, HOMA-IR, lipid profile, and clinical features of PCOS women.

**Schiuma et al. [165]**	Explore the effect of an Hcy-targeted supplement in women with PCOS independently of their fasting blood Hcy baseline levels.	Italy	RCT	32	83-105 days	Rotterdam	n	betaine, l-cystine, chelated zinc, niacin, pyridoxine, riboflavin, 5-methyl tetrahydrofolate, methylcobalamin (combined)	In PCOS ladies, blood homocysteine is increased and inversely correlated with the SHBGs. Physiologic amounts of activated micronutrients in support to the carbon cycle achieve a reduction virtually in all exposed patients
**Kucuk et al. [166]**	Explore the ability of a micronutrient support to decrease Hcy concentration in the follicular fluid of PCOS women undergoing controlled ovarian hyperstimulation for assisted reproduction purposes.	Turkey	RCT	48	2 months before stimulation until 7 weeks after embryo transfer	Rotterdam	y	betaine, l-cystine, zinc, niacin, pyridoxine, riboflavin, 5-methyl tetrahydrofolate, methylcobalamin (combined)	A diet enriched with methyl donors may be useful in PCOS and supplements may also help.
**Santofimia et al. [167]**	Evaluate the efficacy of dietary supplementation with a combination of antioxidants for the modulation of metabolic, endocrine, and clinical parameters in comparison with oral contraception in non-diabetic women newly diagnosed with PCOS	Spain	NRCT	96	6 months	Descriptive	n	Alpha-lipoic acid, N-acetyl cysteine, vitamin B6, S-adenosyl-l-methionine (combined)	This study showed that the antioxidant combination might be a suitable therapy for patients with PCOS when oral contraceptive is not indicated, because in all group's clinical parameters, irregular menstruation as well as androstenedione and quality of life were significantly improved with no statistical difference between groups.
**Hager et al. [168]**	Evaluate whether a micronutrient supplementation preparation that includes a high amount of omega-3 unsaturated acids, other antioxidants and co-enzyme Q10 would have an impact on specific serum parameters in women with PCOS	Austria	RCT	60	3 months	Rotterdam	n	Omega -3, vitamin E, selenium, glycyrrhizin, co-enzyme Q10, folic acid (combined)	A micronutrient supplementation that includes omega-3 fatty acids, folic acid, selenium, vitamin E, catechin, glycyrrhizin, and co-enzyme Q10, given for a minimum of 3 months, is beneficial for women with PCOS in terms of PCOS- specific parameters (LH:FSH ratio, serum testosterone and serum AMH).
**Hernndez-Yero et al. [169]**	Determine the effect of Diamel on the insulin resistance, insulin sensitivity, and sexual hormones results in women with PCOS	Cuba	RCT	37	6 months	Rotterdam	n	Arginine, ascorbic acid, zinc, folic acid, fumaric acid, l-carnitine, sodium methyparaben, cyanocolbalamin, glycine, ornithine, calcium pantothenate, cranberry extract, lettuce extract, l-cysteine, pyridoxal (combined)	The study concluded that the Diamel decreases insulin resistance and improves sensitivity to this hormone in women with PCOS, with improvement in the levels of LH and testosterone.

**Al-Qadhi et al. [170]**	Find the effects of CoQ10 on body weight, serum testosterone level and oxidative stress in women with PCOS.	Iraq	RCT	80	3 months	Rotterdam	n	Co-enzyme Q10	It has been concluded that CoQ10 treatment produces good effects in PCOS patients mainly due to its effects as an antioxidant.
**Ammar et al. [171]**	Evaluate potential benefits of adding the active form of Coenzyme Q10 to Clomiphene Citrate compared with Human Menopausal Gonadotropins in Clomiphene Citrate resistant PCOS patients	Saudi Arabia	RCT	148	3 cycles	Rotterdam	n	Co-enzyme Q10	Addition of co-enzyme-Q10 to Clomiphene Citrate improved ovarian responsiveness in Clomiphene Citrate resistant patients with results comparable to conventional hMG stimulation protocol.
**Karamali et al. [172]**	Evaluate the impact of coenzyme Q10 supplementation on hormonal indices, mental health, and biomarkers of inflammatory responses and oxidative stress among female patients suffering from PCOS	Iraq	RCT	55	12 weeks	Rotterdam	n	Co-enzyme Q10	12-week supplementation of CoQ10 to PCOS women showed beneficial impact on BDI, BAI, hs-CRP, total testosterone, DHEAS, hirsutism, SHBG, TAC and MDA levels.
**Izadi et al. [173]**	Investigate the effects of CoQ10 and/or vitamin E on cardiometabolic outcomes in patients with PCOS.	Iran	RCT	86	8 weeks	Rotterdam	n	Co-enzyme Q10, vitamin E	Co-enzyme-Q10, vitamin E (alone or in combination) had beneficial effects on cardiometabolic outcomes among women with PCOS.
**Refaeey et al. [174]**	Evaluated the effect of combined oral coenzyme Q10 and clomiphene citrate for ovulation induction in clomiphene-citrate-resistant PCOS	Egypt	RCT	101	37 cycles	Rotterdam	n	Co-enzyme Q10	Combination of CoQ10 and clomiphene citrate in the treatment of clomiphene-citrate-resistant PCOS patients improves ovulation and clinical pregnancy rates
**Izadi et al. [175]**	Evaluate the effects of CoQ10 and/or vitamin E on glucose homeostasis parameters and reproductive hormones in women with PCOS.	Iran	RCT	86	8 weeks	Rotterdam	n	Co-enzyme Q10, vitamin E	CoQ10 with or without vitamin E supplementation among women with PCOS had beneficial effects on serum FBS and insulin levels, as well as HOMA-IR and total testosterone levels. However, only co-supplementation affected SHBG concentrations.
**Jamal et al. [176]**	Compare the effects of the CoQ10 and Clomiphene citrate vs Clomiphene citrate alone in induction of ovulation in a Pakistan population	Pakistan	RCT	136	12 months	Rotterdam	n	Co-enzyme Q10	This study showed that the addition of CoQ10 to Clomiphene citrate, increases the chances of ovulation induction.
**Pekcan et al. [177]**	Investigate the effect of coenzyme Q10 supplementation on ovulation induction and intrauterine insemination outcomes in PCOS patients with clomiphene citrate failure or resistance	Turkey	NRCT	130	dose 1 month observed for 3 months	NR	y	Co-enzyme Q10	Short term CoQ10 supplementation was not found to be an effective adjuvant therapy for cumulative pregnancy rates in young infertile women with PCOS who have undergone ovulation induction with intrauterine insemination.
**Sheida et al. [178]**	Investigate the effect of adding L-Carnitine to the gonadotropins on ART outcome in frozen-	Iran	RCT	83	1 IVF cycle till 2 weeks following	Rotterdam	y	L-carnitine	Our result showed that oral L-Carnitine administration during induction of ovulation

	thawed embryo transfer cycles among PCOS women.				positive HHCG test				among PCOS women could not improve laboratory and pregnancy outcome.
**Sangouni et al. [179]**	Investigate the effect of l-carnitine supplementation on insulin resistance, sex hormone-binding globulin (SHBG) and lipid profile in overweight/obese women with PCOS.	Iran	RCT	62	12 weeks	Rotterdam	n	L-carnitine	12-week l-carnitine supplementation in overweight or obese women with PCOS ameliorate insulin resistance, but has no effect on SHBG and lipid profile.
**Tauqir et al. [180]**	Compare the endocrine and metabolic parameters as well as subjective and objective measures of stress in women with PCOS before and after treatment with acetyl-L-carnitine (ALC) and metformin plus pioglitazone.	Pakistan	RCT	147	3 months	AES	n	Acytel-l-carnitine	We conclude that addition of ALC therapy is superior to metformin plus pioglitazone in ameliorating insulin resistance, polycystic ovaries, menstrual irregularities, and hypoadiponectinemia in women with PCOS.
**Ismail et al. [181]**	Evaluate the effectiveness of L-carnitine on improving the ovulation and pregnancy rates as well as adverse metabolic indices in clomiphene-resistant PCOS.	Egypt	RCT	170	12 weeks	Rotterdam	n	L-carnitine	Adding L-carnitine when treating clomiphene-resistant PCOS patients not only improved the quality of ovulation and the pregnancy rate with an acceptable patient tolerability, but also enhanced the patient lipid profile and body mass index.
**Sangouni et al. [182]**	Investigate the effect of L-carnitine supplementation on liver fat content and cardiometabolic outcomes in overweight/obese women with PCOS.	Iran	RCT	62	12 weeks	Rotterdam	n	L-carnitine	L-carnitine supplementation for 12 weeks has no beneficial effect on liver fat content and cardiometabolic outcomes in overweight or obese women with PCOS
**Pakravanfar et al. [183]**	Evaluate the effect of weight loss regimen with and without supplementation	Iran	RCT	62	12 weeks	NR	n	L-carnitine	The present study showed that 1000 mg oral L-carnitine had no significant effect on body weight, body mass index, body composition, and hip circumference, but had a significant effect on waist circumference size.
**Sharkwy et al. [184]**	Evaluate the reproductive and metabolic effects of L-carnitine plus metformin in clomiphene citrate (CC) resistant obese polycystic ovary syndrome (PCOS) women	Egypt	RCT	274	3 months	Rotterdam	n	L-carnitine	L-Carnitine may act synergistically with metformin for improvement of reproductive performance, insulin resistance, and lipid profile in clomiphene-resistant obese PCOS women.
**Sohaei et al. [185]**	Assess the effects of curcumin supplementation on glycaemic status	Iran	RCT	60	6 weeks	Rotterdam	n	Curcumin	Curcumin supplementation might be beneficial for improving serum insulin and QUICKI, however, future investigations are suggested in order to draw a firm link between curcumin and glycemia control.
**Sohrevardi et al. [186]**	Evaluate the effect of metformin with and without curcumin nanomicelles in the treatment of women with PCOS	Iran	RCT	100	3 months	Rotterdam	n	Curcumin	This study showed that curcumin has a synergistic effect with metformin in the

									improvement of insulin resistance and lipid profile in patients with PCOS.
**Asan et al. [187]**	Evaluate curcumin supplementation on the lipid profile and high sensitivity C-reactive protein (hs-CRP) serum levels in women with polycystic ovary syndrome (PCOS).	Turkey	RCT	30	8 weeks	Rotterdam	n	Curcumin	These results indicated that curcumin supplementation added to diet in women with PCOS improved the anthropometric measurements and glycaemic parameters; however, it did not restore the hormone and lipid profiles.
**Heshmati et al. [188]**	Investigate the effect of curcumin in improving blood sugar levels, insulin resistance and hyperandrogenism in individuals with PCOS.	Iran	RCT	72	12 weeks	Rotterdam	n	Curcumin	Curcumin might be a safe and useful supplement to ameliorate PCOS-associated hyperandrogenemia and hyperglycaemia.
**Amooee et al. [189]**	Compare the effects of chromium picolinate vs. metformin in clomiphene citrate resistant PCOS patients.	Iran	RCT	92	3 months	Rotterdam	n	Chromium picolinate	Overall, chromium picolinate was better tolerated compared to metformin; nonetheless, the two study groups were not significantly different regarding ovulation and pregnancy rates.
**Lucidi et al. [190]**	Determine whether chromium supplementation would change insulin sensitivity in women with PCOS and restore normal ovulation.	America	RCT	10	4 months	NIH	n	Chromium picolinate	In women with polycystic ovary syndrome, chromium picolinate (200 μg/d) improves glucose tolerance compared with placebo but does not improve ovulatory frequency or hormonal parameters.
**Ashoush et al. [191]**	Investigate the effect of chromium picolinate on insulin resistance in PCOS.	Egypt	RCT	100	6 months	Rotterdam	n	Chromium picolinate	Chromium picolinate is useful in PCOS to reduce IR and stimulate ovulation.
**Lydic et al. [192]**	Investigate if chromium improved glucose disposal rate (Rd) in PCOS subjects after 2 months of treatment in our pilot study	USA	Single arm	6	2 months	NIH	n	Chromium picolinate	This study suggests that chromium picolinate may be useful as an insulin sensitizer in the treatment of polycystic ovary syndrome.
**Taheri et al. [193]**	Use a placebo-controlled approach with the detailed assessment of the ovarian morphology by applying transvaginal ultrasound to examine the effectiveness of this resveratrol in this group of women.	Iran	RCT	41	3 months	Rotterdam	n	Resveratrol	Treatment with resveratrol significantly reduced the ovarian volume and polycystic ovarian morphology, thus suggesting a disease-modifying effect in PCOS.
**Banaszewska et al. [194]**	Evaluate endocrine and metabolic effects of resveratrol on PCOS.	Poland	RCT	34	3 months	Rotterdam	n	Resveratrol	Resveratrol significantly reduced ovarian and adrenal androgens. This effect may be, at least in part, related to an improvement of insulin sensitivity and a decline of insulin level
**Brenjian et al. [195]**	Evaluate the effect of resveratrol treatment on pro-inflammatory and ER stress markers in patients with PCOS.	Iran	RCT	40	40 days	Rotterdam	y	Resveratrol	Our findings suggest that ER stress is a potential therapeutic target for patients with PCOS.

**Mansour et al. [196]**	Investigate whether resveratrol could improve menstrual dysfunction, clinical signs (i.e., acne and hair loss), and the biochemical evidence of hyperandrogenism in the women with PCOS.	Iran	RCT	78	3 months	Rotterdam	n	Resveratrol	Resveratrol improved menstrual cyclicity and hair loss, even though levels of androgens, insulin and lipids remained unchanged.
**Bahramrezaie et al. [197]**	Describe the effect of resveratrol on the angiogenesis pathway, for management of PCOS through assessing VEGF, HIF1 gene expression, and laboratory parameters.	Iran	RCT	62	40 days	Rotterdam	y	Resveratrol	Based on the results, resveratrol may improve some outcomes of PCOS patients, probably through changing the serum levels of some sex hormones and expression of VEGF & HIF1 genes in the angiogenesis pathway of granulosa cells.
**Hassan et al. [198]**	Compare the effectiveness of combined resveratrol and myoinositol in comparison with the combined metformin and pioglitazone therapy on metabolic parameters (adiponectin and insulin) and hormonal profiles (testosterone, LH, FSH) in women with PCOS.	Pakistan	RCT	110	12 weeks	AES	n	Resveratrol, Myo-inositol	We conclude that the therapeutic intervention with combined resveratrol and myoinositol is more effective in ameliorating altered endocrine, metabolic indices and stress burden and could be of clinical importance in high-risk group of obese, oligo-anovulatory married PCOS affected women.
**Rondanelli et al. [199]**	Evaluate, in normal–overweight PCOS women with normal menses, the effectiveness of berberine on: Insulin resistance, Inflammation, lipid metabolism; Sex hormone profile and symptoms correlated to hyperandrogenism; Body composition and adverse effects.	Italy	s	12	60 days	Rotterdam	n	Berberine	Berberine can represent a safe novel dietary supplement, helpful in treatment strategy for improvements to levels of insulin and sex hormones, as well as anthropometric measures for women with PCOS .
**Wei et al. [200]**	Evaluate the effects of BBR in comparison to metformin (MET) on the metabolic features of women with PCOS.	China	RCT	100	3 months	Rotterdam	n	Berberine	Intake of berberine improved some of the metabolic and hormonal derangements in a group of treated Chinese women with PCOS. Main effects could be related to the changes in body composition in obesity and dyslipidaemia.
**Wu et al. [201]**	Study whether a combination of berberine and letrozole results in higher live births than letrozole alone in infertile women with PCOS	China	RCT	644	6 months	Rotterdam	n	Berberine	Berberine did not add fecundity in PCOS when used in combination with the new ovulation agent letrozole.
**An et al. [202]**	Evaluate the clinical, metabolic and endocrine effects of berberine vs metformin in PCOS women scheduled for IVF treatment and to explore the potential benefits to the IVF process.	China	RCT	150	3 months plus ivf cycle	Rotterdam	y	Berberine	Berberine and metformin treatments prior to IVF improved the pregnancy outcome by normalizing the clinical, endocrine and metabolic parameters in PCOS women. Berberine has a more pronounced therapeutic effect and achieved more live births with fewer side effects than metformin.
**Li et al. [203]**	Evaluate the effects of berberine on the menstrual pattern, ovulation rate, hormonal	China	Single arm	102	4 months	Descriptive	n	Berberine	This study found that administration of berberine alone may improve the menstrual

	and metabolic profiles in anovulatory Chinese women with PCOS								pattern and ovulation rate in anovulatory Chinese women with polycystic ovary syndrome. Berberine can also decrease sex hormone binding globulin, insulin resistance, total cholesterol, triglycerides and low-density lipoprotein cholesterol in normal weight polycystic ovary syndrome women.
**Rashidi et al. [204]**	Investigate the effect of selenium supplementation on ADMA, cardiometabolic risk factors, and hormonal status in women with PCOS.	Iran	RCT	66	12 weeks	Rotterdam	n	Selenium	Selenium supplementation for 12 weeks had beneficial effects on reduction of circulating ADMA and total testosterone levels in women with PCOS. No significant improvements were seen in other cardiometabolic risk factors.
**Mohammad et al. [205]**	Assess whether selenium consumption can improve the metabolic response to insulin and reduce the insulin resistance in these women.	Iran	RCT	53	12 weeks	Rotterdam	n	Selenium	This study showed that selenium supplementation in PCOS patients may worsen insulin resistance in them. Until the results of larger studies become available, indiscriminate consumption of selenium supplements in PCOS patients will warrant caution.
**Alizadeh et al. [206]**	Investigate the effects of melatonin and/or magnesium supplementation on metabolic profile and levels of sex hormones in PCOS women.	Iran	RCT	84	8 weeks	Rotterdam	n	Melatonin, magnesium	Co-supplementation with magnesium and melatonin had beneficial effects on sleep quality and total testosterone. Additionally, combined melatonin and magnesium supplementation was more effective in improving serum levels of cholesterol, LDL-C, HDL-C and insulin, and HOMA-IR.
**Mousavi et al. [207]**	Determine the effects of magnesium and/or melatonin supplementation on metabolic profiles in women with PCOS	Iran	RCT	84	8 weeks	Rotterdam	n	Melatonin, magnesium	Overall, we found a favourable effect of co-supplementation of magnesium and melatonin for 8 weeks in women with PCOS on hirsutism, serum TNF-α, and TAC levels.
**Tagliaferri et al. [208]**	Investigate the effects of 6 months of melatonin administration on clinical, endocrine, and metabolic features of women affected by PCOS	Italy	Single arm	40	6 months	Rotterdam	n	Melatonin	Based on our results, melatonin could be considered a potential future therapeutic agent for women affected by PCOS.
**Al-Qadhi, H. [209]**	Evaluate the effect of supplementation of melatonin on S. LH and body BMI in patients with polycystic ovaries in whom obesity and elevated LH level were important pathological features.	Iraq	NRCT	70	2 months	Descriptive	n	Melatonin	Melatonin treatment has beneficial effects on PCOS patients by affecting s.LH and body weight.

**Mokhtari et al. [210]**	Investigate the effects of melatonin on chemical pregnancy rates of a significant number of PCOS patients undergoing intrauterine insemination.	Iran	RCT	198	1 cycle plus 2 weeks after insemination	Rotterdam	y	Melatonin	The results of this study demonstrated that the treatment of PCOS patients undergoing IUI with melatonin significantly improves the rate of chemical pregnancy
**Farhadian et al. [211]**	Compare the effect of green tea and metformin treatment on the anthropometric indices of women with PCOS	Iran	RCT	45	3 months	Rotterdam	n	Green tea	The use of green tea for patients with PCOS is recommended as a complementary therapy that can have potential effects on obesity.
**Chan et al. [212]**	Study the effects of green tea on bodyweight, and biochemical and hormonal profiles in obese Chinese women with PCOS	China	RCT	34	3 months	Descriptive	n	Green tea	Green tea supplementation did not significantly reduce bodyweight in obese women with PCOS, nor did it alter the glucose or lipid metabolism.
**Tehrani et al. [213]**	Study the effect of green tea on weight and hormonal changes of women suffering from PCOS	Iran	RCT	70	12 weeks	Rotterdam	n	Green tea	The consumption of green tea by overweight and obese women suffering from PCOS leads to weight loss, a decrease in fasting insulin, and a decrease in the level of free testosterone.
**Mombaini et al. [214]**	Determine the effect of a green tea supplement on anthropometric indices and inflammatory factors in women with PCOS	Iran	RCT	45	45 days	Rotterdam	n	Green tea	The present results suggest that daily consumption of green tea tablets did not cause any effect on inflammation biomarkers in PCOS women. However, it may be effective as a complementary treatment for weight control in these women.
**Shirazi et al. [215]**	Evaluate its effects of vitamin E on angiogenic indices in PCOS patients.	Iran	RCT	43	8 weeks	Rotterdam	n	Vitamin E	Vitamin E supplementation for 8 weeks in the PCOS women had beneficial effects on body weight.
**Morsy et al. [216]**	Evaluate the effect of vitamin E on ovulation and pregnancy in women with clomiphene citrate (CC)-resistant PCOS	Egypt	RCT	60	3 menstrual cycles	Rotterdam	n	Vitamin E	The findings of this trial do not support the hypothesis that vitamin E may increase the ovulation and pregnancy rates in women with clomiphene citrate-resistant PCOS.
**Chen et al. [217]**	Determine if short- term supplementation of vitamin E would lead to improved reproductive performance in ovulation induction for PCOS and to explore the associations between vitamin E and pregnancy rates.	China	Observational	321	until miscarriage or delivery	Rotterdam	n	Vitamin E	A short-term supplementation of vitamin E does not alter the pregnancy rate in the ovulation induction cycle.
**Romualdi et al. [218]**	Evaluate the effect of the soy isoflavone genistein on the metabolic and hormonal disturbances of PCOS.	Italy	Single arm	12	6 months	Rotterdam	n	Phytoestrogen	The possible advantages derived from the therapeutic use of phytoestrogens in PCOS are limited to improvement of the lipidic assessment.
**Kamel, H. [219]**	Study the role of a phyto-oestrogen, Cimicifuga racimosa extract (Klimadynon1,	Egypt	RCT	100	3 cycles	NR	n	Phytoestrogen	Phyto-oestrogen can be used as an alternative to clomiphene citrate for ovulation induction in women with polycystic ovarian syndrome.

	Bionorica, Neumarkt, Germany), in ovulation induction in women with PCOS								
**Shahin et al. [220]**	Assess the effect of phyto-oestrogen adjuvant treatment when added to CC induction cycles in patients with PCOS.	Egypt	RCT	206	3 cycles with 2-month rest between each	Rotterdam	n	Phytoestrogen	Adding phyto-oestrogen supplement to clomiphene-induction cycles with timed intercourse in polycystic ovarian syndrome improves cycle outcomes and pregnancy rates.
**Rezvan et al. [221]**	Investigated the effect of quercetin supplementation on the expression of adiponectin receptors at the transcript level in peripheral blood mononuclear cells (PBMC) samples of PCOS patients.	Iran	RCT	84	12 weeks	Rotterdam	n	Quercetin	Oral quercetin supplementation improves the metabolic features of PCOS patients by upregulating the expression of adiponectin receptors and AMPK
**Rezvan et al. [222]**	Determine the effect of quercetin on the adiponectin-mediated insulin sensitivity in PCOS patients.	Iran	RCT	84	12 weeks	Rotterdam	n	Quercetin	Oral quercetin supplementation was effective in improving the adiponectin-mediated insulin resistance and hormonal profile of women with PCOS.
**Khorshidi et al. [223]**	Investigate the effect of quercetin on metabolic and hormonal parameters in overweight or obese women with PCOS	Iran	RCT	78	12 weeks	Rotterdam	n	Quercetin	Quercetin supplementation decreased resistin plasma levels and gene expression, and testosterone and LH concentration in overweight or obese women with PCOS.
**Ghavi et al. [224]**	Investigate and evaluate the effects of fennel essential oil capsules on PCOS symptoms.	Iran	RCT	30	12 weeks	Rotterdam	n	Fennel	Fennel was not effective in alleviating the ovarian cyst symptoms in polycystic women but since the sample size calculation in our small study was based on very large effect sizes, the study might not be powered enough to detect smaller effects.
**Marnani et al. [225]**	Investigated the effects of fennel supplementation with energy-restricted diets on body fat and muscle percentage and insulin resistance in women with PCOS.	Iran	RCT	64	12 weeks	Rotterdam	n	Fennel	High- protein diet and fennel compared with standard diet and placebo had no significant effect on insulin resistance, body fat and muscle.
**Nadjarzadeh et al. [226]**	Evaluate the effect of hypocaloric high-protein, low-carbohydrate weight loss diet supplemented with fennel on anthropometric and androgen indices in overweight and obese women with PCOS	Iran	RCT	64	3 months	Rotterdam	n	Fennel	A hypocaloric high-protein diet along with fennel supplementation could not provide additional improvements in anthropometric and androgen indices among PCOS women.
**Amirkanloo et al. [227]**	Compare the efficacy of fennel and metformin on the insulin resistance of PCOS women.	Iran	RCT	57	3 months	Rotterdam	n	Fennel	Fennel could not improve insulin resistance and anthropometric indices in comparison to metformin in women with polycystic ovary, however, the generalisability of these results is subject to certain limitations and need to be interpreted with caution.

**Schachter et al. [228]**	Examined the effect of B vitamins and/or metformin on plasma Hcy levels in insulin-resistant patients with PCOS wishing to conceive	Iran	RCT	102	up to 3 cycles of IVF	Rotterdam	y	Vitamin B	Plasma homocysteine levels were significantly reduced by both B vitamins and metformin, but to a greater degree by B vitamins, and higher pregnancy rates were associated with vitamin B treatment.
**Palomba et al. [229]**	Evaluate whether the administration of metformin exerts any effects on Hcy levels in PCOS and if folate supplementation enhances the positive effects on the structure and function of the vascular endothelium	Italy	NRCT	50	6 months	NIH	n	Folate	Metformin exerts a slight but significant deleterious effect on serum Hcy levels in patients with PCOS and supplementation with folate is useful to increase the beneficial effect of metformin on the vascular endothelium
**Ammar et al. [230]**	Investigate the potential benefit of adding thymoquinone to metformin in alleviating symptoms of PCOS	Saudi Arabia	RCT	207	6 months	Rotterdam	n	Black cumin oil	Black cumin oil supplementation constitutes a beneficial added value to metformin in ameliorating PCOS-related disorders with resumption of menstrual regularity, weight reduction, change of body fat distribution, and regaining oxidative balance.
**Naeimi et al. [231]**	Evaluate the effect of N. sativa oil on oligo-amenorrhea in patients with PCOS.	Iran	RCT	84	16 weeks	NIH	n	Nigella sativa	Findings suggest that nigella sativa is an alternative treatment and could be useful for menstrual irregularities in women with PCOS.
**Farsinejad-Marj et al. [232]**	Investigate the effect of magnesium supplementation on metabolic profiles and levels of sex hormones in women with PCOS	Iran	RCT	60	8 weeks	Rotterdam	n	Magnesium	This study found evidence indicating that magnesium supplementation did not influence serum lipid profiles and glycaemic indicators among women with PCOS. However, it reduced BMI and testosterone levels as well as increased DHEA concentrations and may increase serum LH levels.
**Muneyyirci-Delale et al. [233]**	Assess the effect of Glucophage, magnesium oxide and spironolactone in altering free fatty acids (FFAs)	USA	RCT	36	12 weeks	Rotterdam	n	Magnesium	The FFA levels were unchanged in the groups treated with Glucophage and magnesium oxide but were significantly decreased in the group treated with spironolactone.
**Swaroop et al. [234]**	Assessed the efficacy of a novel, patent-pending fenugreek (Trigonella foenum-graecum) seed extract enriched in furostanolic saponins (Furocyst), to determine its efficacy in 50 premenopausal women diagnosed with PCOS	India	Single arm	50	90 days	NR	n	Fenugreek seeds extract	In summary, Furocyst was efficacious in ameliorating the symptoms of PCOS.
**Bashtian et al. [235]**	Assessment of fenugreek effects on insulin resistance in women with PCOS.	Iran	RCT	58	8 weeks	Rotterdam	n	Fenugreek seeds extract	Adjuvant therapy to the fenugreek seeds extract (with metformin) in PCOS women improved the sonographic results and menstrual cyclicity.

**Tabrizi et al. [236]**	Evaluated the effects of thylakoid-rich spinach extract supplementation combined with a hypocaloric diet on body composition and serum levels of neopterin, chemerin, and omentin-1 in obese women with PCOS	Iran	RCT	48	12 weeks	Rotterdam	n	Thylakoid-rich spinach extract	Thylakoid-rich spinach extract combined with a low- calorie diet increased circulating omentin-1 and decreased fat mass, abdominal obesity, as well as circulating chemerin, neopterin, and insulin in obese women with PCOS.
**Tabrizi et al. [237]**	Evaluate the effects of spinach-derived thylakoids supplementation combined with a calorie-restricted diet on anthropometric and metabolic profiles in obese women with the PCOS	Iran	RCT	48	12 weeks	Rotterdam	n	Thylakoid-rich spinach extract	Spinach-derived thylakoid supplementation resulted in more favourable improvements in anthropometric indices and insulin sensitivity compared to the calorie restriction alone.
**Battaglia et al. [238]**	Verify the effects of a pill containing drospirenone on the surrogate markers of arterial function and to evaluate the possible improvements induced by the addition of L-arginine.	Italy	RCT	38	6 months	Descriptive	n	L-arginine	Although, the present pilot study was conducted in a limited number of patients, it seems that the L-arginine co-treatment may improve the long-term side effects of the pill reducing the risk of cardiovascular diseases.
**Chen et al. [239]**	Explore the effects of Maitake extract to induce ovulation in patients with PCOS in comparison with and in combination with clomiphene citrate (CC).	USA	RCT	80	12 weeks or 3 cycles (non-responders received combination for up to 16 weeks or 4 cycles	Descriptive	n	Maitake mushroom	
**Shayan et al. [240]**	Compare the effect of Agnugol and Metformin drugs on oligomenorrhea in patients with PCOS.	Iran	RCT	120	7 months	NIH	n	Agnugol	Agnugol and Metformin drugs in the treatment of oligomenorrhea patients with PCOS have the same effect.
**Shahnazi et al. [241]**	Evaluate the effects of a combined low-dose oral contraceptive (LD) and Vitex agnus on the improvement of clinical and paraclinical parameters of PCOS.	Iran	RCT	70	3 cycles	Descriptive	n	Vitex agnus	The effects of low-dose oral contraceptive and Vitex agnus on the normalization of the menstrual cycle and the means of serum prolactin, free testosterone, and DHEA-S levels in the women with PCOS were similar.
**Armanini et al. [242]**	Compare the effect of spironolactone versus spironolactone plus licorice on plasma renin activity, aldosterone and androgen levels in women with PCOS	Italy	RCT	32	60 days	Rotterdam	n	Licorice	In patients with PCOS the mineralocorticoid properties of licorice can reduce the prevalence of side effects related to the diuretic activity of spironolactone.
**Tabrizi et al. [243]**	Evaluate the effect of zinc on inflammatory markers in women with PCOS	Iran	RCT	65	8 weeks	Rotterdam	n	Zinc sulphate	The findings of the present study indicate that zinc supplementation may be considered as an inexpensive adjunct to treatments in patients with polycystic ovary syndrome in the hope of

									reducing cardiovascular disease risk factors, particularly inflammation.
**Tarkesh et al. [244]**	Investigate the effect of oral vitamin K2 (Menaquinone-7 [MK-7]) on clinical and biochemical parameters in PCOS patients.	Iran	RCT	84	8 weeks	Rotterdam	n	Vitamin K2	This study highlights the beneficial effects of MK-7 on insulin resistance, fat mass, skeletal muscle, and serum levels of triglyceride, DHT, and SHBG in PCOS patients.
**Jazani et al. [245]**	Compare the effects of Celery and Anise combination (CAC) and metformin (met.) on oligomenorrhea in PCOS patients.	Iran	RCT	72	16 weeks	Rotterdam	n	Celery plus anise seed extract	This study showed that Celery and Anise combination could regulate menstrual cycles and improve oligomenorrhea in polycystic ovary syndrome patients superiorly to metformin.
**Zadhoush et al. [246]**	Investigate the effect of garlic on the lipid parameters and blood pressure levels in women with PCOS.	Iran	RCT	80	8 weeks	Rotterdam	n	Garlic	The present study suggested that garlic supplementation might be effective on lipid markers improvement.
**Mousavi et al. [247]**	Determining the effect of green coffee supplementation on androgens level in women with PCOS	Iran	RCT	34	6 weeks	Descriptive	n	Green coffee	The results of the clinical trial showed that supplementation of 400 mg green coffee a day for six weeks significantly reduced free testosterone, triglyceride, and cholesterol levels.
**Cheshmeh et al. [248]**	Assess the effects of green cardamom supplementation on the serum level of inflammatory markers and their gene expression among obese women with PCOS.	Iran	RCT	194	4 months	Rotterdam	n	Cardamom	The present study supports the beneficial anti-inflammatory effect of green cardamom on the inflammatory state in PCOS women.
**Qorbani et al. [249]**	Evaluate whether the PCOS-specific serum markers and metabolic parameters would change in the women with PCOS during the three-month administration of oligopin.	Iran	RCT	80	3 months	Rotterdam	n	Oligopin	Oligopin supplementation does not seem to be exerting a beneficial effect on both hormonal and metabolic parameters in the women with PCOS.
**Pourbehi et al. [250]**	Evaluate the effect of psyllium supplementation on insulin resistance and the lipid profile in non-diabetic women with PCOS.	Iran	RCT	54	8 weeks	Rotterdam	n	Psyllium	Psyllium supplementation decreased FBS, fasting insulin, and the lipid profile while improving insulin resistance in non-diabetic women with PCOS.
**Afandak et al. [251]**	Determine the effect of sumac powder on clinical symptoms and laboratory parameters in women with PCOS.	Iran	RCT	88	12 weeks	Rotterdam	n	Sumac	Sumac powder can reduce the inflammatory effects, and glycaemic status and lipid profile of polycystic ovaries in affected women, but has no significant effect on anthropometric parameters and sex hormones.

Abbreviations: ART, Assisted Reproductive Technology; NRCT, Non-randomized controlled trial; NR, Not reported; AES, Androgen Excess Society; RCT, Randomised controlled trial; NIH. National Institutes of Health

Table S3: Details of Scoping reviews

Author	Aim	Reported use of a quality assessment tool	Language/s included in search criteria	Criteria for PCOS diagnosis reported in the inclusion criteria	Includes ART?	Supplement investigated	Findings of primary outcomes
**Lagana et al. [252]**	Summarise the available evidence from randomized controlled trials (RCTs) about the effects of MI supplementation on COH variables (gonadotropins consumption, duration of ovarian stimulation, and number of cancelled cycles), including both PCOS and non-PCOS patients undergoing IVF cycles.	Yes	English	NR	Yes	Myo-inositol	Despite the flaw of available evidence, our data analysis suggests that oral myo-inositoI supplementation is able to reduce GA used in both PCOS and non-PCOS women undergoing IVF. Conversely, this supplement seems able to reduce COH length only in PCOS population.
**Tang et al. [253]**	Assess the effectiveness of insulin sensitising drugs in improving reproductive outcomes and metabolic parameters for women with PCOS and menstrual disturbance.	Yes	all languages	Rotterdam	No	Dchiro-inositol	It would be difficult to make any conclusion regarding the use of Dchiro- inositol as the number of women in this review is small.
**Morley et al. [254]**	Evaluate the effectiveness and safety of insulin-sensitising drugs in improving reproductive and metabolic outcomes for women with PCOS undergoing ovulation induction.	Yes	all languages	Rotterdam	No	Dchiro-inositol	We were unable to draw conclusions regarding Dchiro-inositol as no studies reported primary outcomes
**Mendoza et al. [255]**	Assess the effectiveness of the major inositol stereoisomers, myo-inositol and d-chiro-inositol, in improving reproductive outcomes (oocyte or embryo quality and pregnancy rates) for women with PCOS undergoing ICSI.	Yes	NR	NR	Yes	Myo-inositol, Dchiro-inositol	The data obtained from this systematic review and meta-analysis indicate a lack of arguments to justify that myo-inositol supplementation is sufficient to improve the oocyte or embryo quality and pregnancy rates.
**Facchinetti et al. [256]**	Systematically review randomised studies on comparisons of Metformin and myo-inositol and to perform a meta-analysis in order to compare these two treatments for their short-term efficacy and safety in PCOS patients.	Yes	all languages	Rotterdam, AES	No	Myo-inositol	This meta-analysis demonstrates no differences in the effect of metformin and myo-inositol on short-term hormone changes in subjects with PCOS. The better tolerability of myo-inositol makes it more acceptable for the recovery of androgen and metabolic profile in PCOS women
**Pundir et al. [257]**	Undertake a systematic review to assess the effects of inositol on ovulation induction and reproductive outcomes, as well as on hormonal and glycaemic profile, when compared with placebo and/or metformin or clomiphene, in women with PCOS.	Yes	all languages	NR	No	Myo-inositol, Dchiro-inositol	Inositol appears to significantly improve the ovulation rate, and metabolic and hormonal profiles in women with PCOS compared with placebo.

**Galazis et al. [258]**	Gather all the relevant information concerning the link between DCI and PCOS and specifically to investigate the effects of DCI on ovulation and insulin resistance in PCOS.	Yes	all languages	NR	No	Dchiro-inositol	In summary, it showed that Dchiro-inositol was an effective intervention on the key clinical parameters of interest to women with PCOS such as resumption of ovulation and weight reduction.
**Tang et al. [259]**	Assess the effectiveness of insulin sensitising drugs in improving reproductive outcomes and metabolic parameters for women with PCOS and menstrual disturbance.	Yes	all languages	Rotterdam	No	Dchiro-inositol	It would be difficult to make any conclusion regarding the use of Dchiro- inositol as the number of women in this review is small.
**Showell et al. [260]**	Evaluate the effectiveness and safety of oral supplementation of inositol for reproductive outcomes among subfertile women with PCOS who are trying to conceive.	Yes	all languages	Rotterdam	Yes	Myo-inositol, Dchiro-inositol	In light of available evidence of very low quality, we are uncertain whether myo-inositol improves live birth rate or clinical pregnancy rate in subfertile women with PCOS undergoing IVF pre-treatment taking myo-inositol compared to standard treatment. We are also uncertain whether myo-inositol decreases miscarriage rates or multiple pregnancy rates for these same women compared to standard treatment.
**Zarezadeh et al. [261]**	Assess the impact of inositol supplementation on body mass index through a systematic review and meta-analysis of controlled clinical trials.	Yes	English	NR	No	Myo-inositol, Dchiro-inositol	This study elucidated the significant reducing effect of inositol on BMI in adults younger than 30 years as well as in individuals with PCOS, although the clinical magnitude of this effect may not be much considerable. However, inositol supplementation could be administered as an adjunct therapy to improve anthropometric indices and glycaemic responses.
**Tang et al. [262]**	Assess the effectiveness of insulin sensitising drugs in improving reproductive outcomes and metabolic parameters for women with PCOS and menstrual disturbance.	Yes	all languages	Rotterdam	No	Dchiro-inositol	The number of women remains small. It would be difficult to make any conclusion based on the current findings.
**Lord et al. [263]**	Assess the effectiveness of insulin sensitising drugs in improving clinical and biochemical features of PCOS.	Yes	all languages	Rotterdam	No	Dchiro-inositol	There is no conclusion regarding the use of Dchiro- inositol as the single study was not large enough to analyse.
**Unfer et al. [264]**	Analyse recent randomized clinical trials of inositol(s) in PCOS, in particular myo- and D-chiro-inositol, in order to better elucidate their physiological involvement in PCOS and potential therapeutic use, alone and in conjunction with assisted reproductive	No	all languages	Rotterdam	Yes	Myo-inositol, Dchiro-inositol	This systematic review highlights that oral administration of myo-inositol, alone or in combination with D-chiro-inositol, is capable of restoring spontaneous ovulation and improving fertility in women with PCOS.

	technologies, in the clinical treatment of women with PCOS.						
**Azizi et al. [265]**	Determine and compare the effects of myo-inositol and metformin on hormonal and metabolic profiles and fertility outcomes.	Yes	English	Rotterdam, AES	No	Myo-inositol	The present systematic review could not establish any differences between metformin and myo-inositol concerning the hormonal profile and the ovarian function. However, the findings indicated that myo-inositol could improve fertility outcomes by modulating hyperandrogenism.
**Zeng et al. [266]**	Assess the effectiveness and safety of omega-3 fatty acid for patients with PCOS.	Yes	all languages	NR	No	Myo-inositol	Based on current evidence, myoinositol may be recommended for the treatment of PCOS with insulin resistance, as well as for improving symptoms caused by decreased estrogen in PCOS.
**Zhang et al. [267]**	Perform an updated meta-analysis to evaluate myo-inositol (myo-ins) and the classical insulin sensitizer metformin in terms of efficacy and safety for treating women with PCOS.	Yes	English and Chinese	Rotterdam, AES	No	Myo-inositol plus folic acid (combined), myo-inositol	Compared with metformin, the suitable supplemental dosage of myo-inositol may be helpful in lowering levels of TG and avoiding adverse events.
**Zhao et al. [268]**	The present study aims to compare the effects of oral insulin sensitizers on endocrine and metabolic profiles in women with PCOS.	Yes	all languages	Rotterdam, NIH, AES, Guidelines for diagnosis and treatment of PCOS in China	No	Myo-inositiol, Dchiro-inositol, Berberine	For women with PCOS, myo-inositol combined with Dchiro-inositol and metformin combined with thiazolidinediones appear superior to metformin alone in improving insulin resistance and decreasing total testosterone. Myo-inositol combined with d-chiro-inositol is particularly efficacious in menstrual recovery.
**Unfer et al. [269]**	Provide an overview on the clinical outcomes of MYO as a treatment to improve ovarian function and metabolic and hormonal parameters in women with PCOS.	No	all languages	NR	Yes	Myo-inositol	Myo-inositol mechanism of action appears to be mainly based on improving insulin sensitivity of target tissues, resulting in a positive effect on the reproductive axis and hormonal functions through the reduction of insulin plasma levels.
**Greff et al. [270]**	Evaluate the efficacy and safety of inositols in treating PCOS.	Yes	NR	Rotterdam, Descriptive	No	Inositol	Inositol is an effective and safe treatment in PCOS. Moreover, inositols showed non-inferiority in most outcomes compared to the gold standard treatment, metformin.
**Unanyan et al. [271]**	Compare the effect of metformin, inositol and their combinations on the efficiency in improving outcomes of assisted reproductive technologies in women with PCOS.	Yes	NR	Rotterdam, NIH	Yes	Inositol	Based on our systematic review and meta-analysis, metformin seems to be a good option for improving ART outcomes in women with

							PCOS. However, it is not clear whether inositol usage is adequate.
**Abboud et al. [272]**	Summarise evidence of RCTs on various health effects of a combined supplementation of Vitamin D and probiotics among adults and children	Yes	NR	NR	No	Viamin D, Lactobacillus, Bifidobacterium, Streptococcus.	Beneficial effects included decreased disease severity, improved mental health, metabolic parameters, mainly insulin sensitivity, dyslipidemia, inflammation, and antioxidative capacity, and lower use of healthcare. Co-supplementation of vitamin D and probiotics generated greater health benefits than its comparators did. More studies in other diseases and various populations are needed to confirm these findings and to elucidate the optimal form, composition, and frequency of this co-supplementation.
**Jia et al. [273]**	Investigate the association between the serum level of vitamin D and the risk of PCOS and further examined the therapeutic effect of vitamin D on the clinical features of PCOS.	Yes	English and Chinese	Rotterdam, NIH	No	Vitamin D	This study failed to detect any therapeutic effect of vitamin D3 supplementation in PCOS, which will be a topic of further study.
**Shojaeian et al. [274]**	Review the current evidence and make recommendations about the effect of calcium and vitamin D supplementation on metabolic factors, menstrual cycles and follicular responses in PCOS patients.	Yes	NR	NR	No	Vitamin D plus calcium (combined)	The main finding of this study which is calcium and vitamin D supplementation may improve metabolic parameters and menstrual disturbances in PCOS cases.
**Williams et al. [275]**	Evaluate all epidemiological evidence in the literature linking the effect of vitamin D supplementation to metabolic and hormonal functions in women with PCOS.	No	English	Rotterdam, NIH	No	Vitamin D	We found that vitamin D supplementation had a significant effect on insulin metabolism, total serum testosterone, hirsutism, C-reactive protein, and total antioxidant capacity in women with polycystic ovary syndrome. Evidence from available randomized controlled trials suggests that patients with polycystic ovary syndrome should take vitamin D supplementation for the beneficial effect of metabolic profiles.
**Trummer et al. [276]**	Provide an overview on the effects of vitamin D on PCOS in women and androgen metabolism in men.	No	English	NR	No	Vitamin D	In summary, vitamin D deficiency is associated with adverse fertility outcomes including PCOS and hypogonadism, but the evidence is insufficient to establish causality.
**Guo et al. [277]**	Due to the conflicting results of vitamin D supplementation effects on glucose, lipid, androgenic, and inflammatory profile, we conducted a comprehensive systematic review	Yes	all languages	Rotterdam, NIH	No	Vitamin D	Our systematic review showed that oral intake of vitamin D supplementation attenuated insulin resistance and hyperlipemia, but not androgenic profile or inflammatory markers in

	and meta-analysis focusing on RCTs and studies only employing vitamin D supplementation alone without cosupplementation to reach a more convincing conclusion.						women with PCOS who have vitamin D deficiency.
**Xue et al. [278]**	Investigate the therapeutical effect of vitamin D supplementation on the metabolism and endocrine parameters of PCOS patients.	Yes	English	Rotterdam, NIH	No	Vitamin D	Vitamin D supplementation significantly attenuates serum PTH and triglyceride in PCOS patients but not for serum HOMA-IR, QUICKI, LDL, DHEAS, FT, and TT.
**Pergialiotis et al. [279]**	Investigate the impact of vitamin D supplementation on the hormonal and metabolic profile of PCOS women.	Yes	all languages	NR	No	Vitamin D, calcium	Current data do not support the use of vitamin D supplementation in PCOS patients for the improvement of their hormonal and metabolic status.
**Miao et al. [280]**	Quantitatively summarize the available evidence to assess the effects of vitamin D supplementation on metabolic and hormonal functions in patients with PCOS.	Yes	English	Rotterdam	No	Vitamin D plus calcium (combined)	Data from the available randomized controlled trials suggested vitamin D supplementation reduced insulin resistance and hyperandrogenism, as well improving the lipid metabolism of patients with PCOS to an extent.
**Lagowska et al. [281]**	Assess the effect of vitamin D supplementation on insulin resistance in women with PCOS, based on data available in randomized controlled trials.	Yes	English	Rotterdam	No	Vitamin D	This systematic review provides evidence that vitamin D co-supplementation among PCOS women is effective in decreased fasting glucose concentration and HOMA-IR treatment. Additionally, HOMA-IR also decreased when vitamin D alone was taken every day (not once a week) and in low doses (<4000 IU/d).
**Luo et al. [282]**	Evaluate the effect of vitamin D (alone or with co-supplementation) on lipid profile in PCOS patients.	Yes	all languages	NR	No	Vitamin D plus calcium (combined)	To date, the evidence from RCTs indicated that vitamin D supplementation (alone or with co-supplementation) could statistically improve lipid metabolism, but the effect is small.
**Wang et al. [283]**	Evaluate the effects of vitamin D supplementation on metabolic parameters of PCOS women, based on data available in randomized controlled trials.	Yes	English	Rotterdam, NIH	No	Vitamin D	This meta-analysis indicated that vitamin D supplementation decreases fasting glucose concentration and total cholesterol concentration in PCOS women with vitamin D deficiency.
**Zhao et al. [284]**	Explore the efficacy of vitamin D supplementation at improving hormone metabolism, inflammation, and oxidative stress levels in patients with PCOS.	Yes	all languages	NR	No	Vitamin D	This meta-analysis data demonstrated that vitamin D supplementation could improve TT, hs-CRP, TAC, and MDA in PCOS patients. Vitamin D combined with supplementary

							therapy, vitamin D dose less than 1,000 IU/day, and daily dose frequency appeared to be more conducive to improving hormone, inflammation, and oxidative stress in patients with PCOS.
**Azadi-Yazdi et al. [285]**	Systematically review the published clinical trials regarding the effect of vitamin D supplementation on the androgenic profiles and if possible to conduct a meta-analysis quantifying their results to summarize their result and the possible sources of variation between study results.	Yes	all languages	NR	No	Vitamin D, calcium	The results of the present systematic review and meta-analysis suggest that vitamin D supplement decreases total testosterone in patients with PCOS.
**Han et al. [286]**	Elucidate the possible effect of vitamin D supplementation in PCOS women on hormones, including Luteinizing hormone (LH), follicle-stimulating hormone (FSH), the ratio of LH and FSH (LH/FSH), and the menstrual cycle regularization.	Yes	English	Rotterdam, NIH, AES	No	Vitamin D	Evidence from the existing randomized controlled trials indicated that vitamin D supplementation might improve the LH level and the menstrual cycle regularization but did not have any effect on FSH and LH/FSH levels in PCOS patients.
**Yang et al. [287]**	Evaluate the effect of vitamin D supplementation on pregnancy and ovulation in patients with PCOS	Yes	English and Chinese	Rotterdam	Yes	Vitamin D	Vitamin D supplementation contribute to the higher pregnancy and ovulation rates, and lower androgen, LH, FSH and early miscarriage rates in women with PCOS, regardless of the use of ovulation induction drugs or assisted reproductive technologies. However, no significant improvement was observed in fertilization rate or cleavage rate.
**Devi et al. [288]**	The objective of this study is to explore the efficacy and safety of N-acetyl-cysteine (NAC) as adjuvant therapy in female infertility.	Yes	all languages	NR	Yes	N-acetyl cysteine	N-acetyl cysteine can be an effective adjuvant in PCOS related and unexplained female infertility. The effect could be more profound in women with high BMI, insulin resistance, and oxidative stress.
**Thakker et al. [289]**	To review the benefits and harms of N-acetylcysteine (NAC) in women with polycystic ovary syndrome (PCOS).	Yes	English	Rotterdam, NIH	No	N-acetyl cysteine	N-acetyl cysteine showed significant improvement in pregnancy and ovulation rate as compared to placebo.
**Song et al. [290]**	Evaluate the clinical effectiveness and safety by comparing N-acetyl-cysteine (NAC) with metformin administrated by PCOS patients.	Yes	all languages	Rotterdam	No	N-acetyl cysteine	N-acetyl cysteine significantly reduced BMI and total testosterone, there was no significant difference in pregnancy rate, serum LH level, fasting insulin, and LH/FSH ratio.
**Liu et al. [291]**	This study aimed to comprehensively review published population-based RCTs to evaluate the effect of NAC on metabolic parameters in	Yes	NR	NR	No	N-acetyl cysteine	This study suggests that N-acetyl cysteine is effective in improving metabolic parameters in

	women with PCOS and to obtain preliminary quantitative data on this effect by meta-analysis.						PCOS and may be a promising nutritional supplement for the treatment of PCOS.
**Asl et al. [292]**	Evaluate the potential effects of NAC supplementation on ovulation and sex hormones profile.	Yes	English	NR	No	N-acetyl cysteine	The results indicated that N-acetyl cysteine supplementation decreased TT levels and increased FSH levels. Overall, NAC supplementation might be effective in the improvement of reproductive system function in patients with PCOS.
**Yang et al. [293]**	Assess the effectiveness and safety of omega-3 fatty acid for patients with PCOS	Yes	all languages	NR	No	Omega-3	Based on current evidence, omega-3 fatty acid may be recommended for the treatment of PCOS with insulin resistance as well as high TC (especially LDL-C) and TG.
**Xia et al. [294]**	Evaluate the effect of omega-3 fatty acid supplementation on cardiovascular risk factors in patients with PCOS	Yes	all languages	NR	No	Omega-3	The current meta-analysis demonstrated that omega-3 fatty acid supplementation for women with PCOS resulted in a statistical improvement in insulin, HOMA-IR, TC, triglyceride, LDL-C, VLDL-C, and HDL-C, but did not affect serum glucose.
**Melo et al. [295]**	Verify, based on the scientific data published, if there are any benefits in the omega-3 supplementation in the treatment of PCOS and to indicate its possible dosages for the treatment of polycystic ovary.	NR	NR	NR	No	Omega-3	The selected studies did not show any direct benefits of the omega-3 supplementation in PCOS, this fatty acid clearly promotes indirect benefits by improving the metabolic profile associated with the disease.
**Yuan et al. [296]**	Evaluate the changes in hormones, oxidative stress, and inflammatory factors of PCOS patients who were supplemented with omega-3 polyunsaturated fatty acids (n-3 PUFAs).	Yes	NR	NR	No	Omega-3	This meta-analysis showed that supplementation of n-3 PUFAs in PCOS women can significantly improve CRP, MDA, LH, TT, TAC, and SHBG, but did not affect the concentrations of GSH, DHEAS, FAI, or FSH.
**Sadeghi et al. [297]**	This current meta-analysis is intended to investigate the effect of omega-3 supplementation on insulin resistance in women with PCOS.	Yes	English	NR	No	Omega-3	The results provide evidence that supplementation with omega-3 fatty acids may not have a beneficial effect on improving insulin resistance in women with PCOS.
**Zhou et al. [298]**	Systematically evaluate the efficacy of n-3 PUFA supplementation on metabolic status in patients with PCOS.	Yes	all languages	Rotterdam, NIH, Descriptive	No	Omega-3	The meta-analysis demonstrates that n-3 PUFA may be an effective intervention for alleviating metabolic status in PCOS
**Hajishafiee et al. [299]**	Systematically review clinical trials assessing the effects of n-3 PUFAs consumption on androgen status among adult females with PCOS.	Yes	all languages	NR	No	Omega-3	It does not appear that n-3 PUFAs supplementation significantly affects the androgenic profile of females with PCOS; however, some before-after and long-term

							intervention studies show reduced DHEAS levels.
**Di Tucci et al. [300]**	High- light the role of alpha lipoic acid in the context of female and male infertility.	No	English	NR	Yes	Alpha-lipoic acid alone and combined with Myoinositol, Dchiro-inositol	All the studies showed that a combination of antioxidants may play a very important role in patients affected by endometriosis in the treatment of individual symptoms; further studies are needed to confirm this data.
**Emamat et al. [301]**	Investigate the effectiveness of flaxseed or its oil supplementation on PCOS patients.	Yes	English	Rotterdam, NIH	No	Flaxseed	Based on the results of the present systematic review, we suggest that flaxseed supplementation has the potential to improve metabolic, hormonal, and anthropometric parameters in women with PCOS
**Shirvani-Rad et al. [302]**	Systematically review the meta-analyses of controlled trials and investigate the effects of probiotics on obesity.	Yes	English	NR	No	Probiotics (multi-strain)	It seems that the probiotic products could have beneficial effects as an adjunct therapy for care and management of obesity when used in high dose. However, due to heterogeneity of included studies, it is required to confirm our results by more meta-analyses of clinical trials.
**Shamasbi et al. [303]**	Evaluate the effects of probiotics, prebiotics, and synbiotics on hormonal indicators, such as testosterone, dehydroepiandrosterone sulfate (DHEA-S), sex hormone binding globulin (SHBG), Free Androgen Index (FAI), and inflammatory indicators, such as high sensitive C-reactive protein (hsCRP), malondialdehyde (MDA), total glutathione (GSH), nitric oxide (NO), and total antioxidant capacity (TAC).	Yes	English and Farsi	Rotterdam	No	Prebiotics, probiotics and synbiotics (multi-strain)	Using synbiotics and probiotics in women with polycystic ovary syndrome improve hormonal (FAI, SHBG) and inflammatory (NO, MDA) indices in these patients.
**Cozzolino et al. [304]**	Evaluate the effectiveness of probiotics and synbiotics on metabolic, hormonal and inflammatory parameters of PCOS	Yes	English	Rotterdam	No	Probiotics, synbiotics (multiple-strain)	Probiotics and synbiotics seem to either an effect on/influence metabolic, hormonal and inflammatory parameters, or can influence them. Consequently, it could lead to an improvement of fertility in PCOS.
**Heshmati et al. [305]**	The current study is to evaluate the effect of probiotics (or synbiotics) supplementation in women suffering from polycystic ovary syndrome (PCOS).	Yes	all languages	Rotterdam, NIH	No	Probiotics, synbiotics (multiple-strain)	Although probiotic (or synbiotics) supplementation was effective on some metabolic indices, the effect was negligible and not clinically significant.
**Hadi et al. [306]**	Systematically review the effect of pro-/synbiotic supplementation on anthropometric	Yes	all languages	NR	No	Probiotics, synbiotics (multiple-strain)	The findings suggest that pro-/synbiotic supplementation may improve glucose

	indices, blood glucose level, hormonal, and inflammatory factors in women with PCOS.						homeostasis parameters, hormonal, and inflammatory indices in women with PCOS.
**Talebi et al. [307]**	Examine systematic reviews to determine the effectiveness of pro-, pre-, and synbiotics on hormonal parameters, glycaemic control markers, blood lipids, anthropometric indices, and inflammatory and oxidative stress biomarkers in women with PCOS and update the evidence.	Yes	all languages	NR	No	Prebiotics, probiotics and synbiotics (multi-strain)	Although pro-, pre-, and synbiotics supplementation had beneficial effects on some PCOS-related outcomes, the certainty of the evidence was rated as low to very low.
**Angoorani et al. [308]**	Summarise and critically evaluate the evidence of systematic reviews regarding the effect of probiotics/prebiotics/synbiotics on the management of PCOS.	Yes	English	NR	No	Prebiotics, probiotics and synbiotics (multi-strain)	Our overview confirmed that probiotic supplementation had a potentially beneficial effect on some PCOS-related parameters including body mass index, fasting plasma glucose, and lipid profiles. Evidence shows that synbiotics in comparison with probiotics were less effective on these parameters.
**Heydarpour et al. [309]**	Evaluate the effect of cinnamon supplementation on metabolic parameters of PCOS patients through a systematic review and meta-analysis of clinical trials.	Yes	all languages	NR	No	Cinnamon	The present study indicated that cinnamon supplementation may help PCOS patients to manage their metabolic parameters.
**Heshmati et al. [310]**	This study provides comprehensive information about the effect of cinnamon on insulin resistance (IR) indices in women with PCOS.	Yes	all languages	Rotterdam, NIH, AES	No	Cinnamon	The results indicated that cinnamon supplementation significantly reduced homeostatic model assessment for insulin resistance scores in women with PCOS.
**Keramati et al. [311]**	Evaluate the effects of cinnamon on obesity indices by an umbrella meta-analysis.	Yes	English	NR	No	Cinnamon	The results of this study showed that supplementation with cinnamon significantly reduces BMI and body weight. The impacts were greater in doses of ≥3 g/day and in PCOS patients.
**Maleki et al. [312]**	Determine the potential effect of cinnamon on the metabolic status in the PCOS.	Yes	English	NR	No	Cinnamon	The results showed that increased high-density lipoprotein and insulin sensitivity were increased by the cinnamon supplementation while low-density lipoprotein, triglyceride, and blood glucose were decreased in patients with PCOS
**Sarmadi et al. [313]**	Assess the explicit impact of cinnamon on oxidative stress, inflammation, and lipid profile as main risk factors for cardiovascular health in adult patients.	Yes	English	NR	No	Cinnamon	Our results support the usefulness of cinnamon intake in modulating an imbalanced lipid profile in some metabolic disorders, particularly PCOS, as well as in improving TAC and inter- leukin-6.

**Ahmad et al. [314]**	Review the plant extracts claimed to improve PCOS in the literature.	No	all languages	NR	No	Plant extracts	PCOS characteristics were improved with the use of plant extracts by improving insulin resistance, pregnancy outcomes and ovarian morphology.
**Jazani et al. [315]**	Review herbal medicines used for PCOS worldwide.	No	English	NR	No	Plant extracts	Some plants as natural remedies may have beneficial effects on improving different aspects of PCOS.
**Pundir et al. [316]**	Provide an overview of existing evidence on the effects of non-pharmacological interventions in women with PCOS on fertility and non-fertility outcomes by a review of existing systematic reviews.	Yes	all languages	NR	No	N-acetyl cysteine, omega-3, inositol, vitamin D	N-acetyl-cysteine, inositol and the addition of alternative medicine to ovulation induction agents show preliminary potential to improve fertility.
**Mohseni et al. [317]**	Systematically review all meta-analyses of trials to understand the controversial evidence for the beneficial effects of antioxidative vitamins on dyslipidaemia.	Yes	English	NR	No	Vitamin D, E , C	The beneficial effects of antioxidative vitamins (C, D, E) or their combination with other agents on lipid profile varied based on their dosage, intake duration, and the health status of the individuals.
**Showell et al. [318]**	Determine whether supplementary oral antioxidants compared with placebo, no treatment/standard treatment or another antioxidant improve fertility outcomes for subfertile women.	Yes	English	NR	No	N-acetylcysteine, melatonin, L-arginine, myoinositol, carnitine, selenium, vitamin E, vitamin B, vitamin C, vitamin D plus Calcium, Co Q10, Omega 3 fatty acids.	Antioxidant may benefit subfertile women. Overall, there is no evidence of increased risk of miscarriage, multiple births, gastrointestinal effects or ectopic pregnancies.
**Arentz et al. [319]**	This systematic review examined the effect on menstrual regulation and adverse effects from randomised controlled trials.	Yes	English	Rotterdam, NIH	No	Fish oil, chromium, selenium, vitamin D, vitamin D plus calcium, vitamin B, inositol, camellia sinensis, cimicifuga racemosa, cinnamomum.	There is no high-quality evidence to support the effectiveness of nutritional supplements and herbal medicine for women with PCOS and evidence of safety is lacking.
**Zhang et al. [320]**	Evaluate the efficacy of oral nutritional agents including CoQ10, vitamin E, inositols and vitamin D on androgen-associated hormones, glycolipid metabolism and body weight in women with PCOS.	Yes	all languages	Rotterdam, NIH, AES	No	CoQ10, vitamin E (alone and in combination with CoQ10), vitamin D, or inositols (MI or DCI alone or taken together)	Women with PCOS, inositols supplementation have some certain advantages in increasing SHBG and improving glycolipid metabolism when compared with nutraceuticals like CoQ10, vitamin E, vitamin D. Besides, vitamin E may be a better option in reducing TT and increasing SHBG.
**Florou et al. [321]**	The aim of this study was to systematically review and meta-analyse the best available evidence regarding the effect of CoQ10 supplementation on clinical pregnancy (CPR), live birth (LBR), and miscarriage rates (MR)	Yes	English	NR	Yes	CoQ10	Oral supplementation of CoQ10 may increase CPR when compared with placebo or no-treatment, in women with infertility undergoing ART procedures, without an effect on live birth rate or miscarriages rate.

	compared with placebo or no-treatment in women with infertility undergoing ART.						
**Zhang et al. [322]**	Conduct a meta-analysis of the efficacy and safety of coenzyme Q10 in the treatment of PCOS, and consolidate the evidence of Q10 supplementation on PCOS for the current clinical application.	Yes	all languages	Rotterdam	No	Coenzyme Q10	Based on the current evidence, it could be considered that the addition of CoQ10 is a safe therapy to improve PCOS by improving insulin resistance (reduce HOMA-IR, FINS, FPG), increasing sex hormone levels (increase FSH, reduce testosterone), and improving blood lipids (reduce TG, TC, LDL-C, and increased HDL-C).
**Abdelazeem et al. [323]**	Evaluate the effects of curcumin among patients with PCOS to provide reliable data to clinicians caring for patients with PCOS.	Yes	English	NR	No	Curcumin	The result of our meta-analysis suggests that curcumin is associated with improvement in glycaemic control by decreasing the FBG, insulin level, insulin resistance, and increasing insulin sensitivity when compared with the control group in patients with PCOS. Curcumin was also associated with a decrease in the total cholesterol but has no effect in regard to LDL, triglyceride, HDL, sex hormones, body weight, and CRP.
**Shojaei-Zarghani et al. [324]**	Comprehensively assess all published studies regarding the effects of curcumin/turmeric supplementation on the parameters of PCOS.	Yes	English	NR	No	Curcumin	Present clinical literature is limited to conclude the beneficial effects of curcumin on the management of PCOS symptoms.
**Chien et al. [325]**	Evaluate the effects of curcumin on glycaemic control.	Yes	all languages	NR	No	Curcumin	Curcumin may improve glycaemic control and lipid metabolism in patients with PCOS and metabolic abnormality without significant adverse effects.
**Venkatesan et al. [326]**	Provide a brief review of the existing literature on the association between curcumin supplementation and PCOS.	Yes	English	NR	No	Curcumin	Curcumin supplementation aids in improvement of lipid and glycaemic profiles, body mass index and lowers androgen levels associated with PCOS.
**Liao et al. [327]**	Summarise the clinical evidence of the effects of carnitine on weight management, glycaemic and serum lipids controls in women with PCOS by conducting a meta-analysis of randomized control trials.	Yes	English	NR	No	Carnitine	Based on current evidence, carnitine supplementation in women with PCOS had beneficial effects on weight loss and lipid profiles.
**Gong et al. [328]**	Quantify the effect of carnitine on glucose and lipid metabolic profiles and fertility outcomes in women with PCOS.	Yes	all languages	Rotterdam, AES	No	Carnitine	With the available literature, carnitine seems to improve ovulation and clinical pregnancy and insulin resistance and BMI in women with PCOS.

**Tang et al. [329]**	Derive a more precise estimation of the efficacy of chromium for the management of the clinical symptoms of PCOS.	Yes	all languages	NR	No	Chromium picolinate	Supplementation with chromium may not have significant benefits for women with PCOS.
**Maleki et al. [330]**	Systematically review the effects of chromium on ovarian physiology with a focus on body mass index, as well as hormonal and metabolic dysfunctions in women suffering from PCOS.	No	English	NR	No	Chromium picolinate	The articles reviewed demonstrated that chromium supplementation has limited effects on weight reduction, glucose control, lipid profile, and hormonal disturbance of women with PCOS.
**Fazelian et al. [331]**	Assess the effect of Chromium picolinate supplementation in PCOS that have not yet fully been elucidated.	No	all languages	NR	No	Chromium picolinate	Chromium picolinate supplementation significantly changed fasting insulin, free testosterone and BMI in PCOS patient.
**Shojaei-Zarghani et al. [332]**	Systematically review current animal studies and randomized placebo controlled clinical trials regarding the effects of resveratrol, a natural polyphenolic compound, on PCOS features	Yes	English	NR	No	Resveratrol	The current literature is limited to conclude the beneficial effects of resveratrol on the management of PCOS. Although, according to the promising results of the animal studies and limited RCTs, resveratrol might be an effective phytochemical in PCOS control, especially regarding hormonal and reproductive abnormalities.
**Xie et al. [333]**	Assess the efficacy and safety of berberine on reproductive endocrine and metabolic outcomes in women with PCOS.	Yes	English and Chinese	Descriptive	Yes	Berberine	This review found no solid evidence that berberine could improve live birth or other clinical outcomes in women with PCOS.
**Miraee et al. [334]**	Assess the effect of Berberine (BBR) on women's health to provide greater insights about its effect on women with polycystic syndrome for both patients and health care providers.	Yes	all languages	NR	Yes	Berberine	Berberine led to a significant decrease in waist to hip ratio, profile hormonal insulin resistance, and insulin resistance. Further, androstenedione dropped significantly following treatment with berberine. However, berberine did not have a significant effect on follicle stimulating hormone (FSH) and luteinizing hormone (LH).
**Hajizadeh-Sharafabad et al. [335]**	Evaluate the potential role of selenium in the complications of PCOS.	No	English	NR	No	Selenium	The reviewed studies confirmed inverse relationships between serum selenium levels and some androgenic hormones in PCOS. Selenium is able to attenuate insulin resistance and dyslipidaemia.
**Zhao et al. [336]**	Quantitatively review randomized controlled trials, which examined the effect of the selenium supplementation on PCOS.	Yes	NR	Rotterdam	No	Selenium	Although selenium can improve TAC in PCOS patients, this meta-analysis shows that supplementation of selenium does not significantly improve the level of BMI, Weight, LDL, HDL, Triglycerides, Total Testosterone, HOMA-IR, NO, GSH, MDA and FPG.

**Kazempour et al. [337]**	Assess the impact of selenium supplementation on biochemical markers in women with PCOS.	Yes	NR	Rotterdam	No	Selenium	The study revealed that consuming selenium was associated with increased QUICKI, TAC, and total GSH levels, as well as reduced triglyceride, cholesterol, FPG, insulin, and HOMA-IR levels. Furthermore, regarding SHBG, testosterone levels, MDA, and BMI, no significant differences were observed between the groups.
**Wu et al. [338]**	Explore the efficacy of selenium supplementation for PCOS.	Yes	NR	NR	No	Selenium	Selenium supplementation may be able to improve the metabolic response for polycystic ovary syndrome, and this finding should be interpreted with caution.
**Hu et al. [339]**	Study whether melatonin treatment can increase clinical pregnancy rate and live birth rate in assisted reproductive technology (ART) cycles	Yes	English	NR	Yes	Melatonin	Melatonin treatment significantly increases the clinical pregnancy rate but not live birth rate in ART cycles.
**Shen et al. [340]**	Assess the effectiveness and safety of tea supplements for patients with PCOS.	Yes	all languages	NR	No	Green tea	This meta-analysis suggests that consumption of tea supplementation in women with PCOS could significantly decrease the levels of FBG and FINS as well as reduce body weight.
**Tabrizi et al. [341]**	Evaluate the efficacy of quercetin supplementation in subjects with PCOS.	No	English	NR	No	Quercetin	This review article provides evidence that quercetin could be considered as a potential agent to attenuate PCOS complications.
**Heidari et al. [342]**	Summarise the effects of vitamin E supplementation or vitamin E in combination with omega-3 or magnesium on PCOS.	Yes	NR	NR	No	Vitamin E	This meta-analysis highlights the potential anti-hyperlipidemic, antioxidant, and anti-inflammatory properties of vitamin E supplementation alone or in combination with omega-3 or magnesium on PCOS patients.
**Jafari et al. [343]**	Summarise data from available randomized clinical trials about the impact of folate supplementation on weight and body mass index.	Yes	NR	NR	No	Folic acid	The study outcomes demonstrated that folic acid improves BMI in those with homocysteine levels ≥15 mmol/L and women with PCOS.
**Najafi et al. [344]**	Identify the effect of phytoestrogens on PCOS (clinical evidence and laboratory findings) in women of reproductive age.	Yes	English	NR	No	Phytoestrogen	The currently available knowledge is inconclusive regarding the effect of phytoestrogens on the endocrine disorder of PCOS. These findings should be interpreted with caution due to various limitations/methodological weaknesses of the currently available studies.

Abbreviations: ART, Assisted Reproductive Technology; NRCT, Non-randomized controlled trial; NR, Not reported; AES, Androgen Excess Society; RCT, Randomised controlled trial; NIH, National Institutes of Health

References

1. Agrawal, A.; Mahey, R.; Kachhawa, G.; Khadgawat, R.; Vanamail, P.; Kriplani, A. Comparison of metformin plus myoinositol vs metformin alone in PCOS women undergoing ovulation induction cycles: randomized controlled trial. *Gynecol. Endocrinol.* **2019**, *35*, 511-514, doi:10.1080/09513590.2018.1549656.
2. Nestler, J.E.; Jakubowicz, D.J.; Reamer, P.; Gunn, R.D.; Allan, G. Ovulatory and metabolic effects of D-chiro-inositol in the polycystic ovary syndrome. *N. Engl. J. Med.* **1999**, *340*, 1314-1320, doi:10.1056/NEJM199904293401703.
3. Cheang, K.I.; Baillargeon, J.P.; Essah, P.A.; Ostlund, R.E., Jr.; Apridonize, T.; Islam, L.; Nestler, J.E. Insulin-stimulated release of D-chiro-inositol-containing inositolphosphoglycan mediator correlates with insulin sensitivity in women with polycystic ovary syndrome. *Metabol.* **2008**, *57*, 1390-1397, doi:10.1016/j.metabol.2008.05.008.
4. Emekci Ozay, O.; Ozay, A.C.; Cagliyan, E.; Okyay, R.E.; Gulekli, B. Myo-inositol administration positively effects ovulation induction and intrauterine insemination in patients with polycystic ovary syndrome: a prospective, controlled, randomized trial. *Gynecol. Endocrinol.* **2017**, 1-5, doi:10.1080/09513590.2017.1296127.
5. Costantino, D.; Minozzi, G.; Minozzi, E.; Guaraldi, C. Metabolic and hormonal effects of myo-inositol in women with polycystic ovary syndrome: a double-blind trial. *Eur. Rev. Med. Pharmacol. Sci.* **2009**, *13*, 105-110.
6. Unfer, V.; Carlomagno, G.; Rizzo, P.; Raffone, E.; Roseff, S. Myo-inositol rather than D-chiro-inositol is able to improve oocyte quality in intracytoplasmic sperm injection cycles. A prospective, controlled, randomized trial. *Eur. Rev. Med. Pharmacol. Sci.* **2011**, *15*, 452-457.
7. Genazzani, A.D.; Santagni, S.; Ricchieri, F.; Campedelli, A.; Rattighieri, E.; Chierchia, E.; Marini, G.; Despini, G.; Prati, A.; Simoncini, T. Myo-inositol modulates insulin and luteinizing hormone secretion in normal weight patients with polycystic ovary syndrome. *J. Obstet. Gynaecol. Res.* **2014**, *40*, 1353-1360, doi:10.1111/jog.12319.
8. De Cicco, S.; Immediata, V.; Romualdi, D.; Policola, C.; Tropea, A.; Di Florio, C.; Tagliaferri, V.; Scarinci, E.; Della Casa, S.; Lanzone, A.; et al. Myoinositol combined with alpha-lipoic acid may improve the clinical and endocrine features of polycystic ovary syndrome through an insulin-independent action. *Gynecol. Endocrinol.* **2017**, *33*, 698-701, doi:10.1080/09513590.2017.1313972.
9. Bahadur, A.; Arora, H.; Ravi, A.K.; Naithani, M.; Bahurupi, Y.; Chaturvedi, J.; Ajmani, M.; Mundhra, R. Comparison of Clinical, Metabolic and Hormonal Effects of Metformin Versus Combined Therapy of Metformin With Myoinositol Plus D-

Chiro-Inositol in Women With Polycystic Ovary Syndrome (PCOS): A Randomized Controlled Trial. *Cureus* **2021**, *13*, e15510, doi:10.7759/cureus.15510.
10. Prabhakar, P.; Mahey, R.; Gupta, M.; Khadgawat, R.; Kachhawa, G.; Sharma, J.B.; Vanamail, P.; Kumari, R.; Bhatla, N. Impact of myoinositol with metformin and myoinositol alone in infertile PCOS women undergoing ovulation induction cycles - randomized controlled trial. *Gynecol. Endocrinol.* **2021**, *37*, 332-336, doi:10.1080/09513590.2020.1810657.
11. Lesoine, B.; Regidor, P.-A. Prospective Randomized Study on the Influence of Myoinositol in PCOS Women Undergoing IVF in the Improvement of Oocyte Quality, Fertilization Rate, and Embryo Quality. *Int. J. Endocrinol.* **2016**, 1-5, doi:10.1155/2016/4378507.
12. Troisi, J.; Cinque, C.; Giugliano, L.; Symes, S.; Richards, S.; Adair, D.; Cavallo, P.; Sarno, L.; Scala, G.; Caiazza, M.; et al. Metabolomic change due to combined treatment with myo-inositol, D-chiro-inositol and glucomannan in polycystic ovarian syndrome patients: a pilot study. *J. Ovarian Res.* **2019**, *12*, 25, doi:10.1186/s13048-019-0500-x.
13. Nordio, M.; Basciani, S.; Camajani, E. The 40: 1 myo-inositol/D-chiro-inositol plasma ratio is able to restore ovulation in PCOS patients: comparison with other ratios. *Eur. Rev. Med. Pharmacol. Sci.* **2019**, *23*, 5512-5521, doi:10.26355/eurrev_201906_18223.
14. Stracquadanio, M.; Ciotta, L.; Palumbo, M.A. Effects of myo-inositol, gymnemic acid, and L-methylfolate in polycystic ovary syndrome patients. *Gynecol. Endocrinol.* **2018**, *34*, 495-501, doi:10.1080/09513590.2017.1418852.
15. Januszewski, M.; Issat, T.; Jakimiuk, A.A.; Santor-Zaczynska, M.; Jakimiuk, A.J. Metabolic and hormonal effects of a combined Myo-inositol and d-chiro-inositol therapy on patients with polycystic ovary syndrome (PCOS). *Ginekol. Pol.* **2019**, *90*, 7-10, doi:10.5603/gp.2019.0002.
16. Salehpour, S.; Nazari, L.; Hoseini, S.; Saharkhiz, N.; Ghazi, F.; Sohrabi, M.R. A Potential Therapeutic Role of Myoinositol in the Metabolic and Cardiovascular Profile of PCOS Iranian Women Aged between 30 and 40 Years. *Int. J. Endocrinol.* **2016**, 1-5, doi:10.1155/2016/7493147.
17. Ozay, A.C.; Emekci Ozay, O.; Okyay, R.E.; Cagliyan, E.; Kume, T.; Gulekli, B. Different Effects of Myoinositol plus Folic Acid versus Combined Oral Treatment on Androgen Levels in PCOS Women. *Int. J. Endocrinol.* **2016**, 1-8, doi:10.1155/2016/3206872.

18. Sacchinelli, A.; Venturella, R.; Lico, D.; Di Cello, A.; Lucia, A.; Rania, E.; Cirillo, R.; Zullo, F. The Efficacy of Inositol and N-Acetyl Cysteine Administration (Ovaric HP) in Improving the Ovarian Function in Infertile Women with PCOS with or without Insulin Resistance. *Obstet. Gynecol. Int.* **2014**, 141020-141020, doi:2014/141020.
19. Akhtar, T.; Shaikh, F.; Basma; Ahmed, W.U.N.; Lashari, S.; Bhatti, N. Comparison of myoinositol versus combination of metformin and myoinositol in ovulation induction in polycystic ovarian syndrome. *Pakistan J. Med. Health Sci.* **2021**, *15*, 1494-1496, doi:10.53350/pjmhs211561494.
20. Carlomagno, G.; Antonini, G.; Pacchiarotti, A. Effect of myo-inositol and melatonin versus myo-inositol, in a randomized controlled trial, for improving in vitro fertilization of patients with polycystic ovarian syndrome. *Gynecol. Endocrinol.* **2016**, *32*, 69-73, doi:10.3109/09513590.2015.1101444.
21. Akbari Sene, A.; Tabatabaie, A.; Nikniaz, H.; Alizadeh, A.; Sheibani, K.; Mortezapour Alisaraie, M.; Tabatabaie, M.; Ashrafi, M.; Amjadi, F. The myo-inositol effect on the oocyte quality and fertilization rate among women with polycystic ovary syndrome undergoing assisted reproductive technology cycles: a randomized clinical trial. *Arch. Gynecol. Obstet.* **2019**, *299*, 1701-1707, doi:10.1007/s00404-019-05111-1.
22. Minozzi, M.; Nordio, M.; Pajalich, R. The Combined therapy myo-inositol plus D-Chiro-inositol, in a physiological ratio, reduces the cardiovascular risk by improving the lipid profile in PCOS patients. *Eur. Rev. Med. Pharmacol. Sci.* **2013**, *17*, 537-540.
23. Nordio, M.; Proietti, E. The combined therapy with myo-inositol and D-chiro-inositol reduces the risk of metabolic disease in PCOS overweight patients compared to myo-inositol supplementation alone. *Eur. Rev. Med. Pharmacol. Sci.* **2012**, *16*, 575-581.
24. Alquzwini, T.S. The use of a combination of choline and inositol in the treatment of infertility in women with the polycystic ovarian syndrome. *Int. J. Med. Pharm. Res.* **2020**, *12*, 2777-2780, doi:10.31838/ijpr/2020.12.02.0370.
25. Iuorno, M.J.; Jakubowicz, D.J.; Baillargeon, J.P.; Dillon, P.; Gunn, R.D.; Allan, G.; Nestler, J.E. Effects of d-chiro-inositol in lean women with the polycystic ovary syndrome. *Endocr. Pract.* **2002**, *8*, 417-423, doi:10.4158/EP.8.6.417.
26. Ciotta, L.; Stracquadanio, M.; Pagano, I.; Carbonaro, A.; Palumbo, M.; Gulino, F. Effects of myo-inositol supplementation on oocyte's quality in PCOS patients: a double blind trial. *Eur. Rev. Med. Pharmacol. Sci.* **2011**, *15*, 509-514.
27. Montanino Oliva, M.; Buonomo, G.; Calcagno, M.; Unfer, V. Effects of myo-inositol plus alpha-lactalbumin in myo-inositol-resistant PCOS women. *J. Ovarian Res.* **2018**, *11*, 38, doi:10.1186/s13048-018-0411-2.

28. Fruzzetti, F.; Perini, D.; Russo, M.; Bucci, F.; Gadducci, A. Comparison of two insulin sensitizers, metformin and myo-inositol, in women with polycystic ovary syndrome (PCOS). *Gynecol. Endocrinol.* **2017**, *33*, 39-42, doi:10.1080/09513590.2016.1236078.
29. Colazingari, S.; Treglia, M.; Najjar, R.; Bevilacqua, A. The combined therapy myo-inositol plus D-chiro-inositol, rather than D-chiro-inositol, is able to improve IVF outcomes: results from a randomized controlled trial. *Arch. Gynecol. Obstet.* **2013**, *288*, 1405-1411, doi:10.1007/s00404-013-2855-3.
30. Fruzzetti, F.; Capozzi, A.; Canu, A.; Lello, S. Treatment with d-chiro-inositol and alpha lipoic acid in the management of polycystic ovary syndrome. *Gynecol. Endocrinol.* **2019**, *35*, 506-510, doi:10.1080/09513590.2018.1540573.
31. Benelli, E.; Del Ghianda, S.; Di Cosmo, C.; Tonacchera, M. A Combined Therapy with Myo-Inositol and D-Chiro-Inositol Improves Endocrine Parameters and Insulin Resistance in PCOS Young Overweight Women. *Int. J. Endocrinol.* **2016**, 1-5, doi:10.1155/2016/3204083.
32. Rastegar, F.; Rezaee, Z.; Saedi, N.; Memari, R.; Tajpour, M. Comparison of Effect of Metformin Versus Combination of Folic Acid/Myo-inositol in Infertile Women with Poly Cystic Ovary Syndrome Undergoing in Vitro Fertilization: A Randomized Clinical Trial. *Biomed. Res. Therapy* **2021**, *8*, 4734-4739, doi:10.15419/bmrat.v8i12.710.
33. Soufizadeh, N.; Farhadifar, F.; Seyedoshohadaei, F.; Rezaei, M.; Rasouli, M.A.; Ebrahimpour, K. The effect of inofolic supplementation on women with polycystic ovarian syndrome (Pcos): a randomized clinical trial study. *Ital. J. Gynaecol. Ostet.* **2021**, *33*, 256-262, doi:10.36129/jog.33.04.06.
34. Gerli, S.; Papaleo, E.; Ferrari, A.; Di Renzo, G.C. Randomized, double blind placebo-controlled trial: effects of myo-inositol on ovarian function and metabolic factors in women with PCOS. *Eur. Rev. Med. Pharmacol. Sci.* **2007**, *11*, 347-354.
35. Gerli, S.; Mignosa, M.; Di Renzo, G.C. Effects of inositol on ovarian function and metabolic factors in women with PCOS: a randomized double blind placebo-controlled trial. *Eur. Rev. Med. Pharmacol. Sci.* **2003**, *7*, 151-159.
36. Rajasekaran, K.; Malhotra, N.; Mahey, R.; Khadgawat, R.; Kalaivani, M. Myoinositol versus metformin pretreatment in GnRH-antagonist cycle for women with PCOS undergoing IVF: a double-blinded randomized controlled study. *Gynecol. Endocrinol.* **2021**, doi:10.1080/09513590.2021.1981282.
37. Mendoza, N.; Diaz-Ropero, M.P.; Aragon, M.; Maldonado, V.; Llaneza, P.; Lorente, J.; Mendoza-Tesarik, R.; Maldonado-Lobon, J.; Olivares, M.; Fonolla, J. Comparison of the effect of two combinations of myo-inositol and D-chiro-inositol in

women with polycystic ovary syndrome undergoing ICSI: a randomized controlled trial. *Gynecol. Endocrinol.* **2019**, *35*, 695-700, doi:10.1080/09513590.2019.1576620.
38. Papaleo, E.; Unfer, V.; Baillargeon, J.P.; Fusi, F.; Occhi, F.; De Santis, L. Myo-inositol may improve oocyte quality in intracytoplasmic sperm injection cycles. A prospective, controlled, randomized trial. *Fertil. Steril.* **2009**, *91*, 1750-1754, doi:10.1016/j.fertnstert.2008.01.088.
39. Mendoza, N.; Galan, M.I.; Molina, C.; Mendoza-Tesarik, R.; Conde, C.; Mazheika, M.; Diaz-Ropero, M.P.; Fonolla, J.; Tesarik, J.; Olivares, M. High dose of d-chiro-inositol improves oocyte quality in women with polycystic ovary syndrome undergoing ICSI: a randomized controlled trial. *Gynecol. Endocrinol.* **2020**, *36*, 398-401, doi:10.1080/09513590.2019.1681959.
40. Soldat-Stankovic, V.; Popovic-Pejicic, S.; Stankovic, S.; Prtina, A.; Malesevic, G.; Bjekic-Macut, J.; Livadas, S.; Ognjanovic, S.; Mastorakos, G.; Micic, D.; et al. The effect of metformin and myoinositol on metabolic outcomes in women with polycystic ovary syndrome: role of body mass and adiponectin in a randomized controlled trial. *J. Endocrinol. Invest.* **2022**, *45*, 583-595, doi:10.1007/s40618-021-01691-5.
41. Artini, P.G.; Di Berardino, O.M.; Papini, F.; Genazzani, A.D.; Simi, G.; Ruggiero, M.; Cela, V. Endocrine and clinical effects of myo-inositol administration in polycystic ovary syndrome. A randomized study. *Gynecol. Endocrinol.* **2013**, *29*, 375-379, doi:10.3109/09513590.2012.743020.
42. Kitaya, K.; Takaya, Y.; Nishiyama, R.; Yamaguchi, K.; Matsubayashi, H.; Takeuchi, T.; Mizuta, S.; Ishikawa, T. Myoinositol supplementation on intracytoplasmic sperm injection outcome in Japanese infertile polycystic ovarian syndrome women with non-obese less-androgenic phenotype: A prospective controlled observational study. *Clin. Exp. Obstet. Gynecol.* **2019**, *46*, 549-552, doi:10.12891/ceog4567.2019.
43. Zacche, M.M.; Caputo, L.; Filippis, S.; Zacche, G.; Dindelli, M.; Ferrari, A. Efficacy of myo-inositol in the treatment of cutaneous disorders in young women with polycystic ovary syndrome. *Gynecol. Endocrinol.* **2009**, *25*, 508-513, doi:10.1080/09513590903015544.
44. Isabella, R.; Raffone, E. Does ovary need D-chiro-inositol? *J. Ovarian Res.* **2012**, *5*, doi:10.1186/1757-2215-5-14.
45. Tagliaferri, V.; Romualdi, D.; Immediata, V.; De Cicco, S.; Di Florio, C.; Lanzone, A.; Guido, M. Metformin vs myoinositol: which is better in obese polycystic ovary syndrome patients? A randomized controlled crossover study. *Clin. Endocrinol. (Oxf.)* **2017**, *86*, 725-730, doi:10.1111/cen.13304.

46. Tabatabaie, M.; Amiri, S.; Golestan Jahromi, M.; Sene, A.A.; Zandieh, Z.; Mehdizadeh, M.; Amjadi, F. The effect of Myo-Inositol supplement on molecular regulation of folliculogenesis, steroidogenesis, and assisted reproductive technique outcomes in patients with polycystic ovarian syndrome. *Mol. Biol. Rep.* **2022**, *49*, 875-884, doi:10.1007/s11033-021-06833-9.
47. Piomboni, P.; Focarelli, R.; Capaldo, A.; Stendardi, A.; Cappelli, V.; Cianci, A.; La Marca, A.; Luddi, A.; De Leo, V. Protein modification as oxidative stress marker in follicular fluid from women with polycystic ovary syndrome: the effect of inositol and metformin. *J. Assist. Reprod. Genet.* **2014**, *31*, 1269-1276, doi:10.1007/s10815-014-0307-z.
48. Hernandez Marin, I.; Picconi, O.; Lagana, A.S.; Costabile, L.; Unfer, V. A multicenter clinical study with myo-inositol and alpha-lactalbumin in Mexican and Italian PCOS patients. *Eur. Rev. Med. Pharmacol. Sci.* **2021**, *25*, 3316-3324, doi:10.26355/eurrev_202104_25743.
49. Donà, G.; Sabbadin, C.; Fiore, C.; Bragadin, M.; Giorgino, F.L.; Ragazzi, E.; Clari, G.; Bordin, L.; Armanini, D. Inositol administration reduces oxidative stress in erythrocytes of patients with polycystic ovary syndrome. *Eur J Endocrinol.* **2012**, *166*, 703-710, doi:10.1530/EJE-11-0840.
50. Rolland, A.L.; Peigne, M.; Plouvier, P.; Dumont, A.; Catteau-Jonard, S.; Dewailly, D. Could myo-inositol soft gel capsules outperform clomiphene in inducing ovulation? Results of a pilot study. *Eur. Rev. Med. Pharmacol. Sci.* **2017**, *21*, 10-14.
51. Ozay, A.C.; Emekci Ozay, O.; Okyay, R.E.; Gulekli, B. The effect of myoinositol on ovarian blood flows in women with polycystic ovary syndrome. *Gynecol. Endocrinol.* **2019**, *35*, 237-241, doi:10.1080/09513590.2018.1520827.
52. Morgante, G.; Orvieto, R.; Di Sabatino, A.; Musacchio, M.C.; De Leo, V. The role of inositol supplementation in patients with polycystic ovary syndrome, with insulin resistance, undergoing the low-dose gonadotropin ovulation induction regimen. *Fertil. Steril.* **2011**, *95*, 2642-2644, doi:10.1016/j.fertnstert.2011.01.035.
53. Kamenov, Z.; Kolarov, G.; Gateva, A.; Carlomagno, G.; Genazzani, A.D. Ovulation induction with myo-inositol alone and in combination with clomiphene citrate in polycystic ovarian syndrome patients with insulin resistance. *Gynecol. Endocrinol.* **2015**, *31*, 131-135, doi:10.3109/09513590.2014.964640.
54. Papaleo, E.; Unfer, V.; Baillargeon, J.-P.; De Santis, L.; Fusi, F.; Brigante, C.; Marelli, G.; Cino, I.; Redaelli, A.; Ferrari, A. Myo-inositol in patients with polycystic ovary syndrome: a novel method for ovulation induction. *Gynecol. Endocrinol.* **2007**, *23*, 700-703, doi:10.1080/09513590701672405.

55. Minozzi, M.; Costantino, D.; Guaraldi, C.; Unfer, V. The effect of a combination therapy with myo-inositol and a combined oral contraceptive pill versus a combined oral contraceptive pill alone on metabolic, endocrine, and clinical parameters in polycystic ovary syndrome. *Gynecol. Endocrinol.* **2011**, *27*, 920-924, doi:10.3109/09513590.2011.564685.
56. Nehra, J.; Kaushal, J.; Singhal, S.R.; Ghalaut, V.S. Comparision of myo-inositol versus metformin on anthropometric parameters in polycystic ovarian syndrome in women. *International journal of pharmacy and pharmaceutical sciences* **2017**, *9*, 144-148, doi:10.22159/ijpps.2017v9i4.16359.
57. Nazirudeen, R.; Sridhar, S.; Priyanka, R.; Sumathi, B.; Natarajan, V.; Subbiah, E.; Raghavan, K.S.; Sangumani, J. A randomized controlled trial comparing myoinositol with metformin versus metformin monotherapy in polycystic ovary syndrome. *Clin. Endocrinol. (Oxf.)* **2023**, *99*, 198-205.
58. Isran, B.Z.; Shaikh, S.; Abro, S.H. Two years experience of myo-inositol use in women presented with polycystic ovarian syndrome. *Medical Forum Monthly* **2019**, *30*, 49-53.
59. Vartanyan, E.V.; Tsaturova, K.A.; Devyatova, E.A.; Mikhaylyukova, A.S.; Levin, V.A.; Petuhova, N.L.; Markin, A.V.; Steptsova, E.M. Improvement in quality of oocytes in polycystic ovarian syndrome in programs of in vitro fertilization. *Gynecol. Endocrinol.* **2017**, *33*, 8-11, doi:10.1080/09513590.2017.1399699.
60. Alviggi, C.; Cariati, F.; Conforti, A.; De Rosa, P.; Vallone, R.; Strina, I.; Pivonello, R.; De Placido, G. The effect of FT500 Plus() on ovarian stimulation in PCOS women. *Reprod. Toxicol.* **2016**, *59*, 40-44, doi:10.1016/j.reprotox.2015.10.014.
61. Artini, P.G.; Obino, M.E.R.; Micelli, E.; Malacarne, E.; Vacca, C.; Papini, F.; Cela, V. Effect of d-chiro-inositol and alpha-lipoic acid combination on COH outcomes in overweight/obese PCOS women. *Gynecol. Endocrinol.* **2020**, *36*, 755-759, doi:10.1080/09513590.2020.1737007.
62. Regidor, P.-A.; Schindler, A.E. Myoinositol as a Safe and Alternative Approach in the Treatment of Infertile PCOS Women: A German Observational Study. *Int. J. Endocrinol.* **2016**, 1-5, doi:10.1155/2016/9537632.
63. Yaylali, A.; Bakacak, M.; Bakacak, Z. The efficacy of different insulin-sensitizing agents on treatment outcomes in patients with polycystic ovary syndrome who underwent in-vitro fertilization: A retrospective, record-based, comparative study. *Gynecol. Obstet. Hum. Reprod.* **2021**, *50*, 102006, doi:10.1016/j.jogoh.2020.102006.
64. Fruzzetti, F.; Fidecicchi, T.; Palla, G.; Gambacciani, M. Long-term treatment with alpha-lipoic acid and myo-inositol positively affects clinical and metabolic features of polycystic ovary syndrome. *Gynecol. Endocrinol.* **2020**, *36*, 152-155, doi:10.1080/09513590.2019.1640673.

65. Wdowiak, A. Myoinositol Improves Embryo Development in PCOS Patients Undergoing ICSI. *Int. J. Endocrinol.* **2016**, 1-8, doi:10.1155/2016/6273298.
66. La Marca, A.; Grisendi, V.; Dondi, G.; Sighinolfi, G.; Cianci, A. The menstrual cycle regularization following D-chiro-inositol treatment in PCOS women: a retrospective study. *Gynecol. Endocrinol.* **2015**, *31*, 52-56, doi:10.3109/09513590.2014.964201.
67. Rashad, N.M.; Abd El-Fatah, A.H.; Lashin, M.E.B.; Abomandour, H.G.; Allam, R.M. Impact of vitamin D supplementation on cardio-metabolic status and androgen profile in women with polycystic ovary syndrome: placebo-controlled clinical trial. *Middle East Fertil. Soc. J.* **2019**, *24*, doi:10.1186/s43043-019-0005-y.
68. Al-Bayyari, N.; Al-Domi, H.; Zayed, F.; Hailat, R.; Eaton, A. Androgens and hirsutism score of overweight women with polycystic ovary syndrome improved after vitamin D treatment: a randomized placebo controlled clinical trial. *Clinical nutrition.* **2020**, *40*, 870-878, doi:10.1016/j.clnu.2020.09.024.
69. Javed, Z.; Papageorgiou, M.; Deshmukh, H.; Kilpatrick, E.S.; Mann, V.; Corless, L.; Abouda, G.; Rigby, A.S.; Atkin, S.L.; Sathyapalan, T. A Randomized, Controlled Trial of Vitamin D Supplementation on Cardiovascular Risk Factors, Hormones, and Liver Markers in Women with Polycystic Ovary Syndrome. *Nutrients* **2019**, *11*, 188, doi:10.3390/nu11010188.
70. Abdulameeryahya, A.; Abdulridha, M.K.; Al-Rubuyae, B.J.; Al-Atar, H.A. The effect of vitamin D and Co-enzyme Q10 replacement therapy on hormonal profile and ovulation statusin women with clomiphene citrate resistant polycystic ovary syndrome. *J. Pharm. Sci. Res.* **2019**, *11*, 208-215.
71. Fatemi, F.; Mohammadzadeh, A.; Sadeghi, M.R.; Akhondi, M.M.; Mohammadmoradi, S.; Kamali, K.; Lackpour, N.; Jouhari, S.; Zafadoust, S.; Mokhtar, S.; et al. Role of vitamin E and D3 supplementation in Intra-Cytoplasmic Sperm Injection outcomes of women with polycystic ovarian syndrome: a double blinded randomized placebo-controlled trial. *Clin. Nutr. ESPEN* **2017**, *18*, 23-30, doi:10.1016/j.clnesp.2017.01.002.
72. Firouzabadi, R.d.; Aflatoonian, A.; Modarresi, S.; Sekhavat, L.; MohammadTaheri, S. Therapeutic effects of calcium & vitamin D supplementation in women with PCOS. *Complement. Ther. Clin. Pract.* **2012**, *18*, 85-88, doi:10.1016/j.ctcp.2012.01.005.
73. Kadoura, S.; Alhalabi, M.; Nattouf, A.H. Effect of calcium and vitamin d supplements as an adjuvant therapy to metformin on menstrual cycle abnormalities, hormonal profile, and IGF-1 system in polycystic ovary syndrome patients: a randomized, placebo-controlled clinical trial. *Adv. Pharmacol. Sci.* **2019**, *2019*, doi:10.1155/2019/9680390.

74. Lerchbaum, E.; Theiler-Schwetz, V.; Kollmann, M.; Wölfler, M.; Pilz, S.; Obermayer-Pietsch, B.; Trummer, C.; Barrea, L.; Muscogiuri, G. Effects of Vitamin D Supplementation on Surrogate Markers of Fertility in PCOS Women: A Randomized Controlled Trial. *Nutrients* **2021**, *13*, 547-547, doi:10.3390/nu13020547.
75. Kaddoura, S.; Alhalabi, M.; Nattouf, A.H. The effect of calcium and vitamin d supplements as an adjuvant therapy to metformin on lipid profile in vitamin d deficient/insufficient polycystic ovary syndrome patients. *Res. J. Pharm. Tech.* **2019**, *12*, 1610-1614, doi:10.5958/0974-360X.2019.00268.3.
76. Seyyed Abootorabi, M.; Ayremlou, P.; Behroozi-Lak, T.; Nourisaeidlou, S. The effect of vitamin D supplementation on insulin resistance, visceral fat and adiponectin in vitamin D deficient women with polycystic ovary syndrome: a randomized placebo-controlled trial. *Gynecol. Endocrinol.* **2017**, 1-6, doi:10.1080/09513590.2017.1418311.
77. Rahimi-Ardabili, H.; Pourghassem Gargari, B.; Farzadi, L. Effects of vitamin D on cardiovascular disease risk factors in polycystic ovary syndrome women with vitamin D deficiency. *J. Endocrinol. Invest.* **2013**, *36*, 28-32, doi:10.3275/8303.
78. Wehr, E.; Pieber, T.R.; Obermayer-Pietsch, B. Effect of vitamin D3 treatment on glucose metabolism and menstrual frequency in polycystic ovary syndrome women: a pilot study. *J. Endocrinol. Invest.* **2011**, *34*, 757-763, doi:10.3275/7748.
79. Rasheedy, R.; Sammour, H.; Elkholy, A.; Salim, Y. The efficacy of vitamin D combined with clomiphene citrate in ovulation induction in overweight women with polycystic ovary syndrome: a double blind, randomized clinical trial. *Endocrine* **2020**, *69*, 393-401, doi:10.1007/s12020-020-02315-3.
80. Asadi, M.; Matin, N.; Frootan, M.; Mohamadpour, J.; Qorbani, M.; Tanha, F.D. Vitamin D improves endometrial thickness in PCOS women who need intrauterine insemination: a randomized double-blind placebo-controlled trial. *Arch. Gynecol. Obstet.* **2014**, *289*, 865-870, doi:10.1007/s00404-013-3055-x.
81. Jafari-Sfidvajani, S.; Ahangari, R.; Hozoori, M.; Mozaffari-Khosravi, H.; Fallahzadeh, H.; Nadjarzadeh, A. The effect of vitamin D supplementation in combination with low-calorie diet on anthropometric indices and androgen hormones in women with polycystic ovary syndrome: a double-blind, randomized, placebo-controlled trial. *J. Endocrinol. Invest.* **2017**, 1-11, doi:10.1007/s40618-017-0785-9.
82. Rashidi, H.; Ghaderian, S.B.; Moradi, L. The effect of vitamin D3 on improving lipid profile, fasting glucose and insulin resistance in polycystic ovary syndrome women with vitamin D deficiency. *Middle East Fertil. Soc. J.* **2018**, *23*, 178-183, doi:10.1016/j.mefs.2017.11.002.

83. Rashidi, B.; Haghollahi, F.; Shariat, M.; Zayerii, F. The effects of calcium-vitamin D and metformin on polycystic ovary syndrome: a pilot study. *Taiwan. J. Obstet. Gynecol.* **2009**, *48*, 142-147, doi:10.1016/S1028-4559(09)60275-8.
84. Al-Thuwaynee, S.M.S.; Ahmed, A.R. Effect of vitamin d supplementation on insulin sensitivity and androgen level in vitamin d-deficient polycystic ovary syndrome patients. *Asian J. Pharm. Clin. Res.* **2018**, *11*, 456-458, doi:10.22159/ajpcr.2018.v11i12.29036.
85. Karadağ, C.; Yoldemir, T.; Yavuz, D.G. Effects of vitamin D supplementation on insulin sensitivity and androgen levels in vitamin-D-deficient polycystic ovary syndrome patients. *J. Obstet. Gynaecol. Res.* **2018**, *44*, 270-277, doi:10.1111/jog.13516.
86. Ghazi, S.M.; Farhan, F.S. Evaluation of the hormonal and biochemical changes in obese and non-obese polycystic ovary syndrome (PCOS) Iraqi women before and after vitamin D supplementation. *Int. J. Drug Del. Tech.* **2019**, *9*, 132-136, doi:10.25258/ijddt.v9i3.2.
87. Salehpour, S.; Hosseini, S.; Nazari, L.; Hosseini, M.; Saharkhiz, N. The Effect of Vitamin D Supplementation on Insulin Resistance among Women with Polycystic Ovary Syndrome. *JBRA Assis. Reprod.* **2019**, *23*, 235-238, doi:10.5935/1518-0557.20190032.
88. Bonakdaran, S.; Khorasani, Z.M.; Davachi, B.; Khorasani, J.M. The effects of calcitriol on improvement of insulin resistance, ovulation and comparison with metformin therapy in PCOS patients: a randomized placebo- controlled clinical trial. *Iran J. Reprod. Med.* **2012**, *10*, 465-472.
89. Irani, M.; Seifer, D.B.; Grazi, R.V.; Julka, N.; Bhatt, D.; Kalgi, B.; Irani, S.; Tal, O.; Lambert-Messerlian, G.; Tal, R. Vitamin D Supplementation Decreases TGF-β1 Bioavailability in PCOS: A Randomized Placebo-Controlled Trial. *J. Clin. Endocrinol. Metab.* **2015**, *100*, 4307-4314, doi:10.1210/jc.2015-2580.
90. Raja-Khan, N.; Shah, J.; Stetter, C.M.; Lott, M.E.J.; Kunselman, A.R.; Dodson, W.C.; Legro, R.S. High-dose vitamin D supplementation and measures of insulin sensitivity in polycystic ovary syndrome: a randomized, controlled pilot trial. *Fertil. Steril.* **2014**, *101*, 1740-1746, doi:10.1016/j.fertnstert.2014.02.021.
91. Trummer, C.; Schwetz, V.; Kollmann, M.; Wölfler, M.; Münzker, J.; Pieber, T.R.; Pilz, S.; Heijboer, A.C.; Obermayer-Pietsch, B.; Lerchbaum, E. Effects of vitamin D supplementation on metabolic and endocrine parameters in PCOS: a randomized-controlled trial. *Eur. J. Nutr.* **2019**, *58*, 2019-2028, doi:10.1007/s00394-018-1760-8.
92. Ardabili, H.R.; Gargari, B.P.; Farzadi, L. Vitamin D supplementation has no effect on insulin resistance assessment in women with polycystic ovary syndrome and vitamin D deficiency. *Nutr. Res.* **2012**, *32*, 195-201, doi:10.1016/j.nutres.2012.02.001.

93. Al-Mosawi, R.H.; Hamood, N.; Altaee, H.S. Possible effect of vitamin d3 on follicular maturation in polycyctic ovarian syndrome (Pcos) women. *Indian J. Forensic Med. Toxicol.* **2021**, *15*, 3074-3080, doi:10.37506/ijfmt.v15i2.14844.
94. Figurová, J.; Dravecká, I.; Petríková, J.; Javorský, M.; Lazúrová, I. The effect of alfacalcidiol and metformin on metabolic disturbances in women with polycystic ovary syndrome. *Horm. Mol. Biol. Clin. Investig.* **2017**, *29*, 85-91, doi:10.1515/hmbci-2016-0039.
95. DraveckÁ, I.; FigurovÁ, J.; JavorskÝ, M.; PetrÍKovÁ, J.; VaĽKovÁ, M.; LazÚRovÁ, I. The Effect of Alfacalcidiol and Metformin on Phenotype Manifestations in Women with Polycystic Ovary Syndrome - a Preliminary Study. *Physiol. Res.* **2016**, *65*, 815-822, doi:10.33549/physiolres.933266.
96. Pal, L.; Berry, A.; Coraluzzi, L.; Kustan, E.; Danton, C.; Shaw, J.; Taylor, H. Therapeutic implications of vitamin D and calcium in overweight women with polycystic ovary syndrome. *Gynecol. Endocrinol.* **2012**, *28*, 965-968, doi:10.3109/09513590.2012.696753.
97. Sert, Z.S.; Yilmaz, S.A.; Secilmis, O.; Abusoglu, S.; Unlu, A.; Celik, C. Effect of calcium and vitamin D supplementation on the clinical, hormonal, and metabolic profile in non-obese women with polycystic ovary syndrome. *Ir. J. Med. Sci.* **2022**, doi:10.1007/s11845-021-02899-3.
98. Garg, G.; Kachhawa, G.; Ramot, R.; Khadgawat, R.; Tandon, N.; Sreenivas, V.; Kriplani, A.; Gupta, N. Effect of vitamin D supplementation on insulin kinetics and cardiovascular risk factors in polycystic ovarian syndrome: a pilot study. *Endocr. Connect.* **2015**, *4*, 108-116, doi:10.1530/EC-15-0001.
99. Selimoglu, H.; Duran, C.; Kiyici, S.; Ersoy, C.; Guclu, M.; Ozkaya, G.; Tuncel, E.; Erturk, E.; Imamoglu, S. The effect of vitamin D replacement therapy on insulin resistance and androgen levels in women with polycystic ovary syndrome. *J. Endocrinol. Invest.* **2010**, *33*, 234-238, doi:10.3275/6560.
100. Tehrani, H.G.; Mostajeran, F.; Shahsavari, S. The effect of calcium and vitamin D supplementation on menstrual cycle, body mass index and hyperandrogenism state of women with poly cystic ovarian syndrome. *J. Res. Med. Sci.* **2014**, *19*, 875-880.
101. Kotsa, K.; Yavropoulou, M.P.; Anastasiou, O.; Yovos, J.G. Role of vitamin D treatment in glucose metabolism in polycystic ovary syndrome. *Fertil. Steril.* **2009**, *92*, 1053-1058, doi:10.1016/j.fertnstert.2008.07.1757.
102. Zhuang, L.; Cui, W.; Cong, J.; Zhang, Y. Efficacy of Vitamin D Combined with Metformin and Clomiphene in the Treatment of Patients with Polycystic Ovary Syndrome Combined with Infertility. *Iran J. Public Health* **2019**, *48*, 1802-1809.

103. Kudugunti, N.; Paidipally, S.R.; Himaja, K.; Sahay, R. Effect of vitamin d supplementation on menstrual cycle regularization and metabolic parameters in polycystic ovary syndrome womena retrospective study. *Journal of Krishna Institute of Medical Sciences University* **2021**, *10*, 100-110.
104. Ashok Kumar, M.; Samuel Gideon George, P.; Dasari, A.; Shanmugasundaram, P. A single-blinded randomized trial to evaluate the efficacy of N-acetyl cysteine over metformin in patients with polycystic ovarian syndrome. *Drug invention today* **2018**, *10*, 241-243.
105. Chandil, N.; Pande, S.; Sen, S.S.; Gupta, D. Comparison of Metformin and N Acetylcysteine on Clinical, Metabolic Parameter and Hormonal Profile in Women with Polycystic Ovarian Syndrome. *J. Obstet. Gynaecol. India* **2019**, *69*, 77-81, doi:10.1007/s13224-018-1135-3.
106. Badawy, A.; State, O.; Abdelgawad, S. N-Acetyl cysteine and clomiphene citrate for induction of ovulation in polycystic ovary syndrome: a cross-over trial. *Acta Obstet. Gynecol. Scand.* **2007**, *86*, 218-222, doi:10.1080/00016340601090337.
107. Elnashar, A.; Fahmy, M.; Mansour, A.; Ibrahim, K. N-acetyl cysteine vs. metformin in treatment of clomiphene citrate-resistant polycystic ovary syndrome: a prospective randomized controlled study. *Fertil. Steril.* **2007**, *88*, 406-409, doi:10.1016/j.fertnstert.2006.11.173.
108. Cheraghi, E.; Mehranjani, M.S.; Shariatzadeh, M.A.; Esfahani, M.H.; Ebrahimi, Z. N-Acetylcysteine improves oocyte and embryo quality in polycystic ovary syndrome patients undergoing intracytoplasmic sperm injection: an alternative to metformin. *Reprod. Fertil. Dev.* **2016**, *28*, 723-731, doi:10.1071/RD14182.
109. Cheraghi, E.; Soleimani Mehranjani, M.; Ali Shariatzadeh, M.; Hossein Nasr Esfahani, M.; Ebrahimi, Z. Co-Administration of Metformin and N-Acetyl Cysteine Fails to Improve Clinical Manifestations in PCOS Individual Undergoing ICSI. *Int. J. Fertil. Steril.* **2014**, *8*, 119-128.
110. Nemati, M.; Nemati, S.; Taheri, A.M.; Heidari, B. Comparison of metformin and N-acetyl cysteine, as an adjuvant to clomiphene citrate, in clomiphene-resistant women with polycystic ovary syndrome. *Gynecol. Obstet. Hum. Reprod.* **2017**, *46*, 579-585, doi:10.1016/j.jogoh.2017.07.004.
111. Masha, A.; Manieri, C.; Dinatale, S.; Bruno, G.A.; Ghigo, E.; Martina, V. Prolonged treatment with N-acetylcysteine and L-arginine restores gonadal function in patients with polycistic ovary syndrome. *J. Endocrinol. Invest.* **2009**, *32*, 870-872, doi:10.3275/6185.

112. Waseem, S.; Gohar, S.; Afzal, M.; Wali, Z. Comparison of clomiphene citrate plus n-Acetyl cysteine and clomiphene citrate alone for induction of ovulation in polycystic ovary syndrome. *Pakistan J. Med. Health Sci.* **2021**, *15*, 1253-1255, doi:10.53350/pjmhs211561253.
113. Teimouri, B.; Mollashahi, S.; Paracheh, M.; Farzaneh, F. Comparison of the effect of letrozole alone with letrozole plus n-acetylcysteine on pregnancy rate in patients with polycystic ovarian syndrome: a randomized clinical trial. *Int. J. Womens Health Repr. Sci.* **2021**, *9*, 75-79, doi:10.15296/ijwhr.2021.13.
114. Kilic-Okman, T.; Kucuk, M. N-acetyl-cysteine treatment for polycystic ovary syndrome. *Int. J. Gynecol. Obstet.* **2004**, *85*, 296-297, doi:10.1016/j.ijgo.2004.03.002.
115. Lak, T.B.; Hajshafiha, M.; Nanbakhsh, F.; Oshnouei, S. N-acetyl cysteine in ovulation induction of PCOS women underwent intrauterine insemination: an RCT. *Int. J. Reprod. Biomed.* **2017**, *15*, 203-208.
116. Mostajeran, F.; Tehrani, H.G.; Rahbary, B. N-Acetylcysteine as an adjuvant to letrozole for induction of ovulation in infertile patients with polycystic ovary syndrome. *Adv. Biomed. Res.* **2018**, *7*, 100.
117. Salehpour, S.; Akbari Sene, A.; Saharkhiz, N.; Sohrabi, M.; Moghimian, F. N-acetyl-cysteine as an adjuvant to clomiphene citrate for successful induction of ovulation in infertile patients with polycystic ovary syndrome. *Iran J. Reprod. Med.* **2012**, *10*, 9.
118. Maged, A.M.; Elsawah, H.; Abdelhafez, A.; Bakry, A.; Mostafa, W.A. The adjuvant effect of metformin and N -acetylcysteine to clomiphene citrate in induction of ovulation in patients with Polycystic Ovary Syndrome. *Gynecol. Endocrinol.* **2015**, *31*, 635-638, doi:10.3109/09513590.2015.1037269.
119. Salehpour, S.; Tohidi, M.; Akhound, M.R.; Amirzargar, N. N acetyl cysteine, a novel remedy for poly cystic ovarian syndrome. *Int. J. Fertil. Steril.* **2009**, *3*, 66-73.
120. Gupta, V.; Chaurasia, A.; Khatoon, S.; Singh, U.B. A study of N-acetyl cysteine, metformin and vitamin D3 with calcium on clinical and metabolic profile in PCOS. *Int. J. Reprod. Contracept. Obstet. Gynecol.* **2017**, *6*.
121. Cheraghi, E.; Mehranjani, M.S.; Ali Shariatzadeh, S.M.; Nasr Esfahani, M.H.; Alani, B. N-Acetylcysteine Compared to Metformin, Improves The Expression Profile of Growth Differentiation Factor-9 and Receptor Tyrosine Kinase c-Kit in The Oocytes of Patients with Polycystic Ovarian Syndrome. *Int. J. Fertil. Steril.* **2018**, *11*, 270-278, doi:10.22074/ijfs.2018.5142.

122. Javanmanesh, F.; Kashanian, M.; Rahimi, M.; Sheikhansari, N. A comparison between the effects of metformin and N-acetyl cysteine (NAC) on some metabolic and endocrine characteristics of women with polycystic ovary syndrome. *Gynecol. Endocrinol.* **2016**, *32*, 285-289, doi:10.3109/09513590.2015.1115974.
123. Nasr, A. Effect of N-acetyl-cysteine after ovarian drilling in clomiphene citrate-resistant PCOS women: a pilot study. *Reprod. Biomed. Online* **2010**, *20*, 403-409, doi:10.1016/j.rbmo.2009.12.012.
124. Fulghesu, A.M.; Ciampelli, M.; Muzj, G.; Belosi, C.; Selvaggi, L.; Ayala, G.F.; Lanzone, A. N-acetyl-cysteine treatment improves insulin sensitivity in women with polycystic ovary syndrome. *Fertil. Steril.* **2002**, *77*, 1128-1135, doi:10.1016/s0015-0282(02)03133-3.
125. Rizk, A.Y.; Bedaiwy, M.A.; Al-Inany, H.G. N-acetyl-cysteine is a novel adjuvant to clomiphene citrate in clomiphene citrate-resistant patients with polycystic ovary syndrome. *Fertil. Steril.* **2005**, *83*, 367-370, doi:10.1016/j.fertnstert.2004.07.960.
126. Vine, D.; Proctor, E.; Weaver, O.; Ghosh, M.; Maximova, K.; Proctor, S. A Pilot Trial: Fish Oil and Metformin Effects on ApoB-Remnants and Triglycerides in Women With Polycystic Ovary Syndrome. *J. Endocr. Soc.* **2021**, *5*, 1-12, doi:10.1210/jendso/bvab114.
127. Cussons, A.J.; Watts, G.F.; Mori, T.A.; Stuckey, B.G. Omega-3 fatty acid supplementation decreases liver fat content in polycystic ovary syndrome: a randomized controlled trial employing proton magnetic resonance spectroscopy. *J. Clin. Endocrinol. Metab.* **2009**, *94*, 3842-3848, doi:10.1210/jc.2009-0870.
128. Vargas, M.L.; Almario, R.U.; Buchan, W.; Kim, K.; Karakas, S.E. Metabolic and endocrine effects of long-chain versus essential omega-3 polyunsaturated fatty acids in polycystic ovary syndrome. *Metabolism* **2011**, *60*, 1711-1718, doi:10.1016/j.metabol.2011.04.007.
129. Phelan, N.; O'Connor, A.; Kyaw Tun, T.; Correia, N.; Boran, G.; Roche, H.M.; Gibney, J. Hormonal and metabolic effects of polyunsaturated fatty acids in young women with polycystic ovary syndrome: results from a cross-sectional analysis and a randomized, placebo-controlled, crossover trial. *Am. J. Clin. Nutr.* **2011**, *93*, 652-662, doi:10.3945/ajcn.110.005538.
130. Rafraf, M.; Mohammadi, E.; Asghari-Jafarabadi, M.; Farzadi, L. Omega-3 fatty acids improve glucose metabolism without effects on obesity values and serum visfatin levels in women with polycystic ovary syndrome. *J. Am. Coll. Nutr.* **2012**, *31*, 361-368, doi:10.1080/07315724.2012.10720443.
131. Vaziri, N.; Nadjarzadeh, A.; Daneshbodi, H.; Dehghani Firouzabadi, R.; Lotfi, M.H.; Mozaffari-Khosravi, H. The effect of Omega3 supplementation on androgen profile and menstrual status in women. *Iran J. Reprod. Med.* **2013**, *11*, 45.

132. Trop-Steinberg, S.; Heifetz, E.M.; Azar, Y.; Kafka, I.; Weintraub, A.; Gal, M. Omega-3 Intake Improves Clinical Pregnancy Rate in Polycystic Ovary Syndrome Patients: A Double-Blind, Randomized Study. *Isr. Med. Assoc. J.* **2023**, *25*, 131-136.
133. Nadjarzadeh, A.; Dehghani-Firouzabadi, R.; Daneshbodi, H.; Lotfi, M.H.; Vaziri, N.; Mozaffari-Khosravi, H. Effect of Omega-3 Supplementation on Visfatin, Adiponectin, and Anthropometric Indices in Women with Polycystic Ovarian Syndrome. *J. Reprod. Infertil.* **2015**, *16*, 212-220.
134. Karakas Se Perroud, B.K.T.P.M.F.O. Changes in plasma metabolites and glucose homeostasis during omega-3 polyunsaturated fatty acid supplementation in women with polycystic ovary syndrome. *Biochim. Biophys. Acta* **2016**, *5*, 179-185.
135. Benharrat, L.I.; Senouci, A.; Benhabib, W.; Mekki, K. Omega 3 Supplementation Improves Inflammation and Antioxidant Defense in Women with Polycystic Ovary Syndrome. *Curr. Nutr. Food Sci.* **2022**, *18*, 193-200, doi:10.2174/1573401317666211104121725.
136. Mohammadi, E.; Rafraf, M. Benefits of omega-3 Fatty acids supplementation on serum paraoxonase 1 activity and lipids ratios in polycystic ovary syndrome. *Health Promot. Perspect.* **2012**, *2*, 197-204, doi:10.5681/hpp.2012.023.
137. Khani, B.; Mardanian, F.; Fesharaki, S.J. Omega-3 supplementation effects on polycystic ovary syndrome symptoms and metabolic syndrome. *J. Res. Med. Sci.* **2017**, *22*, 1-6, doi:10.4103/jrms.JRMS_644_16.
138. Mohammadi, E.; Rafraf, M.; Farzadi, L.; Asghari-Jafarabadi, M.; Sabour, S. Effects of omega-3 fatty acids supplementation on serum adiponectin levels and some metabolic risk factors in women with polycystic ovary syndrome. *Asia Pac. J. Clin. Nutr.* **2012**, *21*, 511-518.
139. Fatima Farzana, K.; Abubacker Sulaiman, F.; Ruckmani, A.; Vijayalakshmi, K.; Karunya Lakshmi, G.; Shri Ranjini, S.; Duraivel, M. Effects of flax seeds supplementation in polycystic ovarian syndrome. *Int. J. Pharm. Sci. Rev. Res.* **2015**, *31*, 113-119.
140. Genazzani, A.D.; Shefer, K.; Della Casa, D.; Prati, A.; Napolitano, A.; Manzo, A.; Despini, G.; Simoncini, T. Modulatory effects of alpha-lipoic acid (ALA) administration on insulin sensitivity in obese PCOS patients. *J. Endocrinol. Invest.* **2018**, *41*, 583-590, doi:10.1007/s40618-017-0782-z.
141. Masharani, U.; Gjerde, C.; Evans, J.L.; Youngren, J.F.; Goldfine, I.D. Effects of controlled-release alpha lipoic acid in lean, nondiabetic patients with polycystic ovary syndrome. *J. Diabetes Sci. Technol.* **2010**, *4*, 359-364, doi:10.1177/193229681000400218.

142. Karimi, E.; Heshmati, J.; Shirzad, N.; Vesali, S.; Hosseinzadeh-Attar, M.J.; Moini, A.; Sepidarkish, M. The effect of synbiotics supplementation on anthropometric indicators and lipid profiles in women with polycystic ovary syndrome: a randomized controlled trial. *Lipids Health Dis.* **2020**, *19*, 60, doi:10.1186/s12944-020-01244-4.
143. Zhang, J.; Sun, Z.; Jiang, S.; Bai, X.; Ma, C.; Peng, Q.; Chen, K.; Chang, H.; Fang, T.; Zhang, H. Probiotic Bifidobacterium lactis V9 Regulates the Secretion of Sex Hormones in Polycystic Ovary Syndrome Patients through the Gut-Brain Axis. *mSystems* **2019**, *4*, doi:10.1128/mSystems.00017-19.
144. Ghanei, N.; Rezaei, N.; Amiri, G.A.; Zayeri, F.; Makki, G.; Nasseri, E. The probiotic supplementation reduced inflammation in polycystic ovary syndrome: A randomized, double-blind, placebo-controlled trial. *J. Funct. Foods* **2018**, *42*, 306-311, doi:10.1016/j.jff.2017.12.047.
145. Karimi, E.; Moini, A.; Yaseri, M.; Shirzad, N.; Sepidarkish, M.; Hossein-Boroujerdi, M.; Javad Hosseinzadeh-Attar, M. Effects of synbiotic supplementation on metabolic parameters and apelin in women with polycystic ovary syndrome: a randomised double-blind placebo-controlled trial - Corrigendum. *Br. J. Nutr.* **2020**, 1-, doi:10.1017/S0007114520001488.
146. Darvishi, S.; Rafraf, M.; Asghari-Jafarabadi, M.; Farzadi, L. Synbiotic Supplementation Improves Metabolic Factors and Obesity Values in Women with Polycystic Ovary Syndrome Independent of Affecting Apelin Levels: A Randomized Double-Blind Placebo - Controlled Clinical Trial. *Int. J. Fertil. Steril.* **2021**, *15*, 51-59, doi:10.22074/ijfs.2021.6186.
147. Gholizadeh Shamasbi, S.; Dehghan, P.; Charandabi, S.-A.; Aliasgarzadeh, A.; Mirghafourvand, M. Effect of prebiotic on anthropometric indices in women with polycystic ovarian syndrome: a triple-blind, randomized, controlled clinical trial. *Iran. Red Crescent Med. J.* **2018**, *20*, doi:10.5812/ircmj.67270.
148. Chudzicka-Strugała, I.; Kubiak, A.; Banaszewska, B.; Zwozdziak, B.; Siakowska, M.; Pawelczyk, L.; Duleba, A.J. Effects of Synbiotic Supplementation and Lifestyle Modifications on Women With Polycystic Ovary Syndrome. *J. Clin. Endocrinol. Metab.* **2021**, *106*, 2566-2573, doi:10.1210/clinem/dgab369.
149. Gholizadeh Shamasbi, S.; Dehgan, P.; Mohammad-Alizadeh Charandabi, S.; Aliasgarzadeh, A.; Mirghafourvand, M. The effect of resistant dextrin as a prebiotic on metabolic parameters and androgen level in women with polycystic ovarian syndrome: a randomized, triple-blind, controlled, clinical trial. *Eur. J. Nutr.* **2019**, *58*, 629-640, doi:10.1007/s00394-018-1648-7.
150. Lagowska, K.; Drzymala-Czyz, S. A low glycemic index, energy-restricted diet but not Lactobacillus rhamnosus supplementation changes fecal short-chain fatty acid and serum lipid concentrations in women with overweight or obesity and polycystic ovary syndrome. *Eur. Rev. Med. Pharmacol. Sci.* **2022**, *26*, 917-926, doi:10.26355/eurrev_202202_28001.

151. Kaur, I.; Suri, V.; Sachdeva, N.; Rana, S.V.; Medhi, B.; Sahni, N.; Ahire, J.; Singh, A. Efficacy of multi-strain probiotic along with dietary and lifestyle modifications on polycystic ovary syndrome: a randomised, double-blind placebo-controlled study. *Eur. J. Nutr.* **2022**, *61*, 4145-4154.
152. Talaat, B.; Ammar, I.M.M. The added value of cinnamon to metformin in controlling symptoms of polycystic ovary syndrome, a randomized controlled trial. *Middle East Fertil. Soc. J.* **2018**, *23*, 440-445, doi:10.1016/j.mefs.2018.03.005.
153. Kort, D.H.; Lobo, R.A. Preliminary evidence that cinnamon improves menstrual cyclicity in women with polycystic ovary syndrome: a randomized controlled trial. *Am. J. Obstet. Gynecol.* **2014**, *211*, 487.e481-486, doi:10.1016/j.ajog.2014.05.009.
154. Wiweko, B.; Susanto, C. The Effect of Metformin and Cinnamon on Serum Anti-Mullerian Hormone in Women Having PCOS: a Double-Blind, Randomized, Controlled Trial. *J. Hum. Reprod. Sci.* **2017**, *10*, 31-36, doi:10.4103/jhrs.JHRS_90_16.
155. Mika, T.; Ayaka, T.; Miki, K.; Tsutomu, K.; Keisuke, F.; Borzoei, A.; Rafraf, M.; Asghari-Jafarabadi, M. Cinnamon improves metabolic factors without detectable effects on adiponectin in women with polycystic ovary syndrome. *Asia Pac. J. Clin. Nutr.* **2018**, *27*, 293-299, doi:10.6133/apjcn.062017.13.
156. Borzoei, A.; Rafraf, M.; Niromanesh, S.; Farzadi, L.; Narimani, F.; Doostan, F. Effects of cinnamon supplementation on antioxidant status and serum lipids in women with polycystic ovary syndrome. *J. Tradit. Complement. Med.* **2018**, *8*, 128-133, doi:10.1016/j.jtcme.2017.04.008.
157. Hajimonfarednejad, M.; Nimrouzi, M.; Heydari, M.; Zarshenas, M.M.; Raee, M.J.; Jahromi, B.N. Insulin resistance improvement by cinnamon powder in polycystic ovary syndrome: a randomized double-blind placebo controlled clinical trial. *J. Reprod. Infertil.* **2018**, *19*, 120-121.
158. Khan, A.A.; Begum, W. Efficacy of Darchini in the management of polycystic ovarian syndrome: A randomized clinical study. *J. Herb. Med.* **2019**, *15*, doi:10.1016/j.hermed.2018.11.005.
159. Wang, J.G.; Anderson, R.; Nakhuda, G.S.; Guarnaccia, M.M.; Sauer, M.V.; Lobo, R.A. The effect of cinnamon extract on insulin resistance in polycystic ovary syndrome: a pilot study. *Fertil. Steril.* **2006**, *86*, S9.
160. Permadi, W.; Hestiantoro, A.; Ritonga, M.A.; Ferrina, A.I.; Iswari, W.A.; Sumapraia, K.; Muharram, R.; Djuwantono, T.; Wiweko, B.; Tjandrawinata, R. Administration of Cinnamon and Lagersroemia speciosa Extract on Lipid Profile of Polycystic Ovarian Syndrome Women with High Body Mass Index. *J. Hum. Reprod. Sci.* **2021**, *14*, 16-20, doi:10.4103/jhrs.JHRS_141_20.

161. Kazemi, M.; Lalooha, F.; Nooshabadi, M.R.; Dashti, F.; Kavianpour, M.; Haghighian, H.K. Randomized double blind clinical trial evaluating the Ellagic acid effects on insulin resistance, oxidative stress and sex hormones levels in women with polycystic ovarian syndrome. *J. Ovarian Res.* **2021**, *14*, 100, doi:10.1186/s13048-021-00849-2.
162. Gharaei, R.; Alyasin, A.; Mahdavinezhad, F.; Samadian, E.; Ashrafnezhad, Z.; Amidi, F. Randomized controlled trial of astaxanthin impacts on antioxidant status and assisted reproductive technology outcomes in women with polycystic ovarian syndrome. *J. Assist. Reprod. Genet.* **2022**, doi:10.1007/s10815-022-02432-0.
163. Arentz, S.; Smith, C.A.; Abbott, J.; Fahey, P.; Cheema, B.S.; Bensoussan, A. Combined Lifestyle and Herbal Medicine in Overweight Women with Polycystic Ovary Syndrome (PCOS): a Randomized Controlled Trial. *Phytother. Res.* **2017**, *31*, 1330-1340, doi:10.1002/ptr.5858.
164. Ainehchi, N.; Khaki, A.; Ouladsahebmadarek, E.; Hammadeh, M.; Farzadi, L.; Farshbaf-Khalili, A.; Asnaashari, S.; Khamnei, H.J.; Khaki, A.A.; Shokoohi, M. The effect of clomiphene citrate, herbal mixture, and herbal mixture along with clomiphene citrate on clinical and para-clinical parameters in infertile women with polycystic ovary syndrome: a randomized controlled clinical trial. *Arch. Med. Sci.* **2020**, *16*, 1304-1318, doi:10.5114/aoms.2020.93271.
165. Schiuma, N.; Costantino, A.; Bartolotti, T.; Dattilo, M.; Bini, V.; Aglietti, M.C.; Renga, M.; Favilli, A.; Falorni, A.; Gerli, S. Micronutrients in support to the one carbon cycle for the modulation of blood fasting homocysteine in PCOS women. *J. Endocrinol. Invest.* **2020**, *43*, 779-786, doi:10.1007/s40618-019-01163-x.
166. Kucuk, T.; Horozal, P.E.; Karakulak, A.; Timucin, E.; Dattilo, M. Follicular homocysteine as a marker of oocyte quality in PCOS and the role of micronutrients. *J. Assist. Reprod. Genet.* **2023**, 1-9.
167. Pingarrón Santofímia, C.; Poyo Torcal, S.; López Verdú, H.; Henríquez Linares, A.; Calvente Aguilar, V.; Terol Sánchez, P.; Martínez García, M.S.; Lafuente González, P. Evaluation of the efficacy of an antioxidant combination for the modulation of metabolic, endocrine, and clinical parameters in patients with polycystic ovary syndrome. *Gynecol. Endocrinol.* **2023**, *39*, 2227277.
168. Hager, M.; Nouri, K.; Imhof, M.; Egarter, C.; Ott, J. The impact of a standardized micronutrient supplementation on PCOS-typical parameters: a randomized controlled trial. *Arch. Gynecol. Obstet.* **2019**, *300*, 455-460, doi:10.1007/s00404-019-05194-w.
169. Hernndez-Yero, A.; Santana Pérez, F.; Ovies Carballo, G.; Cabrera-Rode, E. Diamel therapy in polycystic ovary syndrome reduces hyperinsulinaemia, insulin resistance, and hyperandrogenaemia. *Int. J. Endocrinol.* **2012**, *2012*, doi:10.1155/2012/382719.

170. Al-Qadhi, H.I.; Kadhim, E.J.; Ali, R.H. Coenzyme Q10 effects on body weight, serum testosterone level and oxidative stress in women with polycystic ovarian syndrome (PCOS). *International journal of research in pharmaceutical sciences* **2017**, *8*, 378-383.
171. Ammar, I.M.M.; Abdou, A.M. Effect of Ubiquinol supplementation on ovulation induction in Clomiphene Citrate resistance. *Middle East Fertil. Soc. J.* **2021**, *26*, doi:10.1186/s43043-021-00070-7.
172. Karamali, M.; Gholizadeh, M. The effects of coenzyme Q10 supplementation on metabolic profiles and parameters of mental health in women with polycystic ovary syndrome. *Gynecol. Endocrinol.* **2021**, doi:10.1080/09513590.2021.1991910.
173. Izadi, A.; Shirazi, S.; Taghizadeh, S.; Gargari, B.P. Independent and Additive Effects of Coenzyme Q10 and Vitamin E on Cardiometabolic Outcomes and Visceral Adiposity in Women With Polycystic Ovary Syndrome. *Arch. Med. Res.* **2019**, *50*, 1-10, doi:10.1016/j.arcmed.2019.04.004.
174. El Refaeey, A.; Selem, A.; Badawy, A. Combined coenzyme Q10 and clomiphene citrate for ovulation induction in clomiphene-citrate-resistant polycystic ovary syndrome. *Reprod. Biomed. Online* **2014**, *29*, 119-124, doi:10.1016/j.rbmo.2014.03.011.
175. Izadi, A.; Ebrahimi, S.; Shirazi, S.; Taghizadeh, S.; Parizad, M.; Farzadi, L.; Gargari, B.P. Hormonal and Metabolic Effects of Coenzyme Q10 and/or Vitamin E in Patients With Polycystic Ovary Syndrome. *J. Clin. Endocrinol. Metab.* **2019**, *104*, 319-327, doi:10.1210/jc.2018-01221.
176. Jamal, H.; Waheed, K.; Mazhar, R.; Sarwar, M.Z. Comparative Study Of Combined Co-Enzyme Q10 And Clomiphene Citrate Vs Clomiphene Citrate Alone For Ovulation Induction In Patients With Polycystic Ovarian Syndrome. *J. Pak. Med. Assoc.* **2023**, *73*, 1502-1505.
177. Kuru Pekcan, M.; Tokmak, A.; Özaksit, G. Effect of short term supplementation of coenzyme q10 on cumulative pregnancy rates in young infertilewomen with polycystic ovarian syndrome. *J. Clin. Obstet. Gynecol.* **2019**, *29*, 62-67, doi:10.5336/jcog.2019-66096.
178. Sheida, A.; Davar, R.; Tabibnejad, N.; Eftekhar, M. The effect of adding L-Carnitine to the GnRH-antagonist protocol on assisted reproductive technology outcome in women with polycystic ovarian syndrome: a randomized clinical trial. *Gynecol. Endocrinol.* **2021**, doi:10.1080/09513590.2021.1878135.
179. Sangouni, A.A.; Pakravanfar, F.; Ghadiri-Anari, A.; Nadjarzadeh, A.; Fallahzadeh, H.; Hosseinzadeh, M. The effect of l-carnitine supplementation on insulin resistance, sex hormone-binding globulin and lipid profile in overweight/obese women with polycystic ovary syndrome: a randomized clinical trial. *Eur. J. Nutr.* **2021**, doi:10.1007/s00394-021-02659-0.

180. Tauqir, S.; Israr, M.; Rauf, B.; Malik, M.O.; Habib, S.H.; Shah, F.A.; Usman, M.; Raza, M.A.; Shah, I.; Badshah, H.; et al. Acetyl-L-Carnitine Ameliorates Metabolic and Endocrine Alterations in Women with PCOS: A Double-Blind Randomized Clinical Trial. *Adv. Ther.* **2021**, *38*, 3842-3856, doi:10.1007/s12325-021-01789-5.
181. Ismail, A.M.; Hamed, A.H.; Saso, S.; Thabet, H.H. Adding L-carnitine to clomiphene resistant PCOS women improves the quality of ovulation and the pregnancy rate. A randomized clinical trial. *Eur. J. Obstet. Gynecol. Reprod. Biol.* **2014**, *180*, 148-152, doi:10.1016/j.ejogrb.2014.06.008.
182. Sangouni, A.A.; Sasanfar, B.; Ghadiri-Anari, A.; Hosseinzadeh, M. Effect of l-carnitine supplementation on liver fat content and cardiometabolic indices in overweight/obese women with polycystic ovary syndrome: A randomized controlled trial. *Clin. Nutr. ESPEN* **2021**, *46*, 54-59, doi:10.1016/j.clnesp.2021.08.005.
183. Pakravanfar, F.; Ghadiri-Anari, A.; Nadjarzadeh, A.; Fallahzadeh, H.; Hosseinzadeh, M. The Effect of L-Carnitine Supplementation on Weight and Body Composition in Women with Polycystic Ovary Syndrome: A Double-Blind Randomized Clinical Trial. *J. Nutr. Food Security* **2020**, *5*, 282-289, doi:10.18502/JNFS.V5I3.3801.
184. El Sharkwy, I.; Sharaf El-Din, M. l-Carnitine plus metformin in clomiphene-resistant obese PCOS women, reproductive and metabolic effects: a randomized clinical trial. *Gynecol. Endocrinol.* **2019**, *35*, 701-705, doi:10.1080/09513590.2019.1576622.
185. Sohaei, S.; Amani, R.; Tarrahi, M.J.; Ghasemi-Tehrani, H. The effects of curcumin supplementation on glycemic status, lipid profile and hs-CRP levels in overweight/obese women with polycystic ovary syndrome: A randomized, double-blind, placebo-controlled clinical trial. *Complement. Ther. Med.* **2019**, *47*, 102201.
186. Sohrevardi, S.M.; Heydari, B.; Azarpazhooh, M.R.; Teymourzadeh, M.; Simental-Mendía, L.E.; Atkin, S.L.; Sahebkar, A.; Karimi-Zarchi, M. Therapeutic Effect of Curcumin in Women with Polycystic Ovary Syndrome Receiving Metformin: a Randomized Controlled Trial. *Adv. Exp. Med. Biol.* **2021**, *1308*, 109-117, doi:10.1007/978-3-030-64872-5_9.
187. Asan, S.A.; Bas, M.; Eren, B.; Karaca, E. The effects of curcumin supplementation added to diet on anthropometric and biochemical status in women with polycystic ovary syndrome: A randomized, placebo-controlled trial. *Progress Nutr.* **2020**, *22*, doi:10.23751/pn.v22i4.10460.
188. Heshmati, J.; Moini, A.; Sepidarkish, M.; Morvaridzadeh, M.; Salehi, M.; Palmowski, A.; Mojtahedi, M.F.; Shidfar, F. Effects of curcumin supplementation on blood glucose, insulin resistance and androgens in patients with polycystic ovary syndrome: A randomized double-blind placebo-controlled clinical trial. *Phytomedicine* **2021**, *80*, N.PAG-N.PAG, doi:10.1016/j.phymed.2020.153395.

189. Amooee, S.; Parsanejad, M.E.; Ravanbod Shiraze, M.; Alborzi, S.; Samsami, A. Metformin versus chromium picolinate in clomiphene citrate-resistant patients with polycystic ovary. *Int. J. Fertil. Steril.* **2012**, *6*, 106-107.
190. Lucidi, R.S.; Thyer, A.C.; Easton, C.A.; Holden, A.E.C.; Schenken, R.S.; Brzyski, R.G. Effect of chromium supplementation on insulin resistance and ovarian and menstrual cyclicity in women with polycystic ovary syndrome. *Fertil. Steril.* **2005**, *84*, 1755-1757, doi:10.1016/j.fertnstert.2005.06.028.
191. Ashoush, S.; Abou-Gamrah, A.; Bayoumy, H.; Othman, N. Chromium picolinate reduces insulin resistance in polycystic ovary syndrome: Randomized controlled trial. *J. Obstet. Gynaecol. Res.* **2016**, *42*, 279-285, doi:10.1111/jog.12907.
192. Lydic, M.L.; McNurlan, M.; Bembo, S.; Mitchell, L.; Komaroff, E.; Gelato, M. Chromium picolinate improves insulin sensitivity in obese subjects with polycystic ovary syndrome. *Fertil. Steril.* **2006**, *86*, 243-246, doi:10.1016/j.fertnstert.2005.11.069.
193. Taheri, A.P.H.; Moradi, B.; Radmard, A.R.; Sanginabadi, M.; Qorbani, M.; Mohajeri-Tehrani, M.R.; Shirzad, N.; Hosseini, S.; Hekmatdoost, A.; Asadi, S.; et al. Effect of resveratrol administration on ovarian morphology, determined by transvaginal ultrasound for the women with PCOS. *Br. J. Nutr.* **2021**, doi:10.1017/S0007114521003330.
194. Banaszewska, B.; Wrotyńska-Barczyńska, J.; Spaczynski, R.Z.; Pawelczyk, L.; Duleba, A.J. Effects of Resveratrol on Polycystic Ovary Syndrome: A Double-blind, Randomized, Placebo-controlled Trial. *J. Clin. Endocrinol. Metab.* **2016**, jc20161858-jc20161858.
195. Brenjian, S.; Moini, A.; Yamini, N.; Kashani, L.; Faridmojtahedi, M.; Bahramrezaie, M.; Khodarahmian, M.; Amidi, F. Resveratrol treatment in patients with polycystic ovary syndrome decreased pro-inflammatory and endoplasmic reticulum stress markers. *Am. J. Reprod. Immunol.* **2020**, *83*, e13186, doi:10.1111/aji.13186.
196. Mansour, A.; Samadi, M.; Sanginabadi, M.; Gerami, H.; Karimi, S.; Hosseini, S.; Shirzad, N.; Hekmatdoost, A.; Mahdavi-Gorabi, A.; Mohajeri-Tehrani, M.R.; et al. Effect of resveratrol on menstrual cyclicity, hyperandrogenism and metabolic profile in women with PCOS. *Clinical Nutrition* **2021**, *40*, 4106-4112, doi:10.1016/j.clnu.2021.02.004.
197. Bahramrezaie, M.; Amidi, F.; Aleyasin, A.; Saremi, A.; Aghahoseini, M.; Brenjian, S.; Khodarahmian, M.; Pooladi, A. Effects of resveratrol on VEGF & HIF1 genes expression in granulosa cells in the angiogenesis pathway and laboratory parameters of polycystic ovary syndrome: a triple-blind randomized clinical trial. *J. Assist. Reprod. Genet.* **2019**, *36*, 1701-1712, doi:10.1007/s10815-019-01461-6.

198. Hassan, S.; Shah, M.; Malik, M.O.; Ehtesham, E.; Habib, S.H.; Rauf, B. Treatment with combined resveratrol and myoinositol ameliorates endocrine, metabolic alterations and perceived stress response in women with PCOS: a double-blind randomized clinical trial. *Endocrine* **2023**, *79*, 208-220.
199. Rondanelli, M.; Riva, A.; Petrangolini, G.; Allegrini, P.; Giacosa, A.; Fazia, T.; Bernardinelli, L.; Gasparri, C.; Peroni, G.; Perna, S. Berberine Phospholipid Is an Effective Insulin Sensitizer and Improves Metabolic and Hormonal Disorders in Women with Polycystic Ovary Syndrome: A One-Group Pretest–Post-Test Explanatory Study. *Nutrients* **2021**, *13*, 3665-3665, doi:10.3390/nu13103665.
200. Wei, W.; Zhao, H.; Wang, A.; Sui, M.; Liang, K.; Deng, H.; Ma, Y.; Zhang, Y.; Zhang, H.; Guan, Y. A clinical study on the short-term effect of berberine in comparison to metformin on the metabolic characteristics of women with polycystic ovary syndrome. *Eur J Endocrinol.* **2012**, *166*, 99-105, doi:10.1530/EJE-11-0616.
201. Wu, X.-K.; Wang, Y.-Y.; Liu, J.-P.; Liang, R.-N.; Xue, H.-Y.; Ma, H.-X.; Shao, X.-G.; Ng, E.H.Y. Randomized controlled trial of letrozole, berberine, or a combination for infertility in the polycystic ovary syndrome. *Fertil. Steril.* **2016**, *106*, 757-765.e751, doi:10.1016/j.fertnstert.2016.05.022.
202. An, Y.; Sun, Z.; Zhang, Y.; Liu, B.; Guan, Y.; Lu, M. The use of berberine for women with polycystic ovary syndrome undergoing IVF treatment. *Clin. Endocrinol. (Oxf.)* **2014**, *80*, 425-431, doi:10.1111/cen.12294.
203. Li, L.; Li, C.; Pan, P.; Chen, X.; Wu, X.; Ng, E.H.Y.; Yang, D. A single arm pilot study of effects of berberine on the menstrual pattern, ovulation rate, hormonal and metabolic profiles in anovulatory Chinese women with polycystic ovary syndrome. *PLoS One* **2015**, *10*, doi:10.1371/journal.pone.0144072.
204. Rashidi, B.H.; Mohammad Hosseinzadeh, F.; Alipoor, E.; Asghari, S.; Yekaninejad, M.S.; Hosseinzadeh-Attar, M.J. Effects of Selenium Supplementation on Asymmetric Dimethylarginine and Cardiometabolic Risk Factors in Patients with Polycystic Ovary Syndrome. *Biol. Trace Elem. Res.* **2020**, *196*, 430-437, doi:10.1007/s12011-019-01954-6.
205. Mohammad Hosseinzadeh, F.; Hosseinzadeh-Attar, M.J.; Yekaninejad, M.S.; Rashidi, B. Effects of selenium supplementation on glucose homeostasis and free androgen index in women with polycystic ovary syndrome: a randomized, double blinded, placebo controlled clinical trial. *J. Trace Elem. Med. Biol.* **2016**, *34*, 56-61, doi:10.1016/j.jtemb.2016.01.002.
206. Alizadeh, M.; Karandish, M.; Asghari Jafarabadi, M.; Heidari, L.; Nikbakht, R.; Babaahmadi Rezaei, H.; Mousavi, R. Metabolic and hormonal effects of melatonin and/or magnesium supplementation in women with polycystic ovary

syndrome: a randomized, double-blind, placebo-controlled trial. *Nutr. Metab. (Lond.)* **2021**, *18*, 57, doi:10.1186/s12986-021-00586-9.
207. Mousavi, R.; Alizadeh, M.; Asghari Jafarabadi, M.; Heidari, L.; Nikbakht, R.; Babaahmadi Rezaei, H.; Karandish, M. Effects of Melatonin and/or Magnesium Supplementation on Biomarkers of Inflammation and Oxidative Stress in Women with Polycystic Ovary Syndrome: a Randomized, Double-Blind, Placebo-Controlled Trial. *Biol. Trace Elem. Res.* **2022**, *200*, 1010-1019, doi:10.1007/s12011-021-02725-y.
208. Tagliaferri, V.; Romualdi, D.; Scarinci, E.; Cicco, S.D.; Florio, C.D.; Immediata, V.; Tropea, A.; Santarsiero, C.M.; Lanzone, A.; Apa, R. Melatonin Treatment May Be Able to Restore Menstrual Cyclicity in Women With PCOS: A Pilot Study. *Reprod. Sci.* **2018**, *25*, 269-275, doi:10.1177/1933719117711262.
209. Al-Qadhi, H.I. Effect of melatonin supplementation on serum lh level and bmi in women with polycystic ovarian syndrome. *J. Pharm. Sci. Res.* **2018**, *10*, 1-4.
210. Mokhtari, F.; Asbagh, F.A.; Azmoodeh, O.; Bakhtiyari, M.; Almasi-Hashiani, A. Effects of Melatonin Administration on Chemical Pregnancy Rates of Polycystic Ovary Syndrome Patients Undergoing Intrauterine Insemination: A Randomized Clinical Trial. *Int. J. Fertil. Steril.* **2019**, *13*, 225-229, doi:10.22074/ijfs.2019.5717.
211. Farhadian, M.; Barati, S.; Mahmoodi, M.; Barati Mosleh, A.; Yavangui, M. Comparison of green tea and metformin effects on anthropometric indicators in women with polycystic ovarian syndrome: A clinical trial study. *J. Rep. Pharm. Sci.* **2020**, *9*, 97-103, doi:10.4103/jrptps.JRPTPS_14_19.
212. Chan, C.C.; Koo, M.W.; Ng, E.H.; Tang, O.S.; Yeung, W.S.; Ho, P.C. Effects of Chinese green tea on weight, and hormonal and biochemical profiles in obese patients with polycystic ovary syndrome--a randomized placebo-controlled trial. *J. Soc. Gynecol. Investig.* **2006**, *13*, 63-68, doi:10.1016/j.jsgi.2005.10.006.
213. Tehrani, H.G.; Allahdadian, M.; Zarre, F.; Ranjbar, H.; Allahdadian, F. Effect of green tea on metabolic and hormonal aspect of polycystic ovarian syndrome in overweight and obese women suffering from polycystic ovarian syndrome: A clinical trial. *J. Educ. Health Promot.* **2017**, *6*, 36, doi:10.4103/jehp.jehp_67_15.
214. Mombaini, E.; Jafarirad, S.; Husain, D.; Haghighizadeh, M.H.; Padfar, P. The Impact of Green Tea Supplementation on Anthropometric Indices and Inflammatory Cytokines in Women with Polycystic Ovary Syndrome. *Phytother. Res.* **2017**, *31*, 747-754, doi:10.1002/ptr.5795.

215. Shirazi, S.; Pourghassem Gargari, B.; Izadi, A.; Taghizadeh, S.; Parizad, M. Effect of Vitamin E on Serum Levels of Vascular Endothelial Growth Factor and Angiopoietin-1 in Women with Polycystic Ovary Syndrome: A Pilot Randomized, Placebo-Controlled Trial. *Int. J. Fertil. Steril.* **2021**, *15*, 44-50.
216. Morsy, A.A.; Sabri, N.A.; Mourad, A.M.; Mojahed, E.M.; Shawki, M.A. Randomized controlled open-label study of the effect of vitamin E supplementation on fertility in clomiphene citrate-resistant polycystic ovary syndrome. *J. Obstet. Gynaecol. Res.* **2020**, *46*, 2375-2382, doi:10.1111/jog.14467.
217. Chen, J.; Guo, Q.; Pei, Y.-h.; Ren, Q.-l.; Chi, L.; Hu, R.-k.; Tan, Y. Effect of a short-term vitamin E supplementation on oxidative stress in infertile PCOS women under ovulation induction: a retrospective cohort study. *BMC Womens Health* **2020**, *20*, 1-9, doi:10.1186/s12905-020-00930-w.
218. Romualdi, D.; Costantini, B.; Campagna, G.; Lanzone, A.; Guido, M. Is there a role for soy isoflavones in the therapeutic approach to polycystic ovary syndrome? Results from a pilot study. *Fertil. Steril.* **2008**, *90*, 1826-1833, doi:10.1016/j.fertnstert.2007.09.020.
219. Kamel, H.H. Role of phyto-oestrogens in ovulation induction in women with polycystic ovarian syndrome. *Eur. J. Obstet. Gynecol. Reprod. Biol.* **2013**, *168*, 60-63, doi:10.1016/j.ejogrb.2012.12.025.
220. Shahin, A.Y.; Mohammed, S.A. Adding the phytoestrogen Cimicifugae Racemosae to clomiphene induction cycles with timed intercourse in polycystic ovary syndrome improves cycle outcomes and pregnancy rates - a randomized trial. *Gynecol. Endocrinol.* **2014**, *30*, 505-510, doi:10.3109/09513590.2014.895983.
221. Rezvan, N.; Moini, A.; Gorgani-Firuzjaee, S.; Hosseinzadeh-Attar, M.J. Oral quercetin supplementation enhances adiponectin receptor transcript expression in polycystic ovary syndrome patients: a randomized placebo-controlled double-blind clinical trial. *Cell J.* **2018**, *19*, 627-633, doi:10.22074/cellj.2018.4577.
222. Rezvan, N.; Moini, A.; Janani, L.; Mohammad, K.; Saedisomeolia, A.; Nourbakhsh, M.; Gorgani-Firuzjaee, S.; Mazaherioun, M.; Hosseinzadeh-Attar, M.J. Effects of Quercetin on Adiponectin-Mediated Insulin Sensitivity in Polycystic Ovary Syndrome: a Randomized Placebo-Controlled Double-Blind Clinical Trial. *Horm. Metab. Res.* **2016**, *(no pagination)*, doi:10.1055/s-0042-118705.
223. Khorshidi, M.; Moini, A.; Alipoor, E.; Rezvan, N.; Gorgani-Firuzjaee, S.; Yaseri, M.; Hosseinzadeh-Attar, M.J. The effects of quercetin supplementation on metabolic and hormonal parameters as well as plasma concentration and gene expression of

resistin in overweight or obese women with polycystic ovary syndrome. *Phytother. Res.* **2018**, *32*, 2282-2289, doi:10.1002/ptr.6166.
224. Ghavi, F.; Taghizadeh, M.; Taebi, M.; Abdolahian, S. Effect of Foeniculum vulgare essence on symptoms of polycystic ovarian syndrome (PCOS): A randomized double-blind, placebo-controlled trial. *J. Herb. Med.* **2019**, *17-18*, doi:10.1016/j.hermed.2019.100277.
225. Hosseini Marnani, E.; Ghadiri-Anari, A.; Ramezani-Jolfaie, N.; Mohammadi, M.; abdollahi, N.; Namayandeh, S.M.; Mozaffari-Khosravi, H.; Salehi-Abargouei, A.; Nadjarzadeh, A. Effect of fennel supplementation along with high-protein, low-carbohydrate weight-loss diet on insulin resistance and percentage of fat and muscle mass in overweight/obese women with polycystic ovary syndrome. *J. Funct. Foods* **2020**, *67*, doi:10.1016/j.jff.2020.103848.
226. Nadjarzadeh, A.; Ghadiri-Anari, A.; Ramezani-Jolfaie, N.; Mohammadi, M.; Salehi-Abargouei, A.; Namayande, S.M.; Mozaffari-Khosravi, H.; Hosseini-Marnani, E. Effect of hypocaloric high-protein, low-carbohydrate diet supplemented with fennel on androgenic and anthropometric indices in overweight and obese women with polycystic ovary syndrome: A randomized placebo-controlled trial. *Complement. Ther. Med.* **2021**, *56*, N.PAG-N.PAG, doi:10.1016/j.ctim.2020.102633.
227. Amirkhanloo, F.; Esmaeilzadeh, S.; Mirabi, P.; Abedini, A.; Amiri, M.; Saghebi, R.; Golsorkhtabaramiri, M. Comparison of Foeniculum Vulgare versus metformin on insulin resistance and anthropometric indices of women with polycystic ovary, an open-label controlled trial study. *Obesity Medicine* **2022**, *31*, doi:10.1016/j.obmed.2022.100401.
228. Schachter, M.; Raziel, A.; Strassburger, D.; Rotem, C.; Ron-El, R.; Friedler, S. Prospective, randomized trial of metformin and vitamins for the reduction of plasma homocysteine in insulin-resistant polycystic ovary syndrome. *Fertil. Steril.* **2007**, *88*, 227-230, doi:10.1016/j.fertnstert.2006.11.071.
229. Palomba, S.; Falbo, A.; Giallauria, F.; Russo, T.; Tolino, A.; Zullo, F.; Colao, A.; Orio, F. Effects of metformin with or without supplementation with folate on homocysteine levels and vascular endothelium of women with polycystic ovary syndrome. *Diabetes Care* **2010**, *33*, 246-251, doi:10.2337/dc09-1516.
230. Ammar, I.M.M.; Salem, M.A.A. Amelioration of polycystic ovary syndrome-related disorders by supplementation of thymoquinone and metformin. *Middle East Fertil. Soc. J.* **2021**, *26*, doi:10.1186/s43043-021-00076-1.
231. Naeimi, S.A.; Tansaz, M.; Hajimehdipoor, H.; Saber, S. Comparing the effect of Nigella sativa oil soft gel and placebo on oligomenorrhea, amenorrhea and laboratory characteristics in patients with polycystic ovarian syndrome, a randomized clinical trial. *Res. J. Pharmacogn.* **2020**, *7*, 49-58, doi:10.22127/rjp.2019.206178.1530.

232. Farsinejad-Marj, M.; Azadbakht, L.; Mardanian, F.; Saneei, P.; Esmaillzadeh, A. Clinical and Metabolic Responses to Magnesium Supplementation in Women with Polycystic Ovary Syndrome. *Biol. Trace Elem. Res.* **2020**, *196*, 349-358, doi:10.1007/s12011-019-01923-z.
233. Muneyyirci-Delale, O.; Kaplan, J.; Joulak, I.; Yang, L.; Von Gizycki, H.; Nacharaju, V.L. Serum free fatty acid levels in PCOS patients treated with glucophage, magnesium oxide and spironolactone. *Gynecol. Endocrinol.* **2013**, *29*, 474-477, doi:10.3109/09513590.2013.769515.
234. Swaroop, A.; Jaipuriar, A.S.; Gupta, S.K.; Bagchi, M.; Kumar, P.; Preuss, H.G.; Bagchi, D. Efficacy of a Novel Fenugreek Seed Extract (Trigonella foenum-graecum, Furocyst) in Polycystic Ovary Syndrome (PCOS). *Int. J. Med. Sci.* **2015**, *12*, 825-831, doi:10.7150/ijms.13024.
235. Bashtian, M.H.; Emami, S.A.; Mousavifar, N.; Esmaily, H.A.; Mahmoudi, M.; Poor, A.H.M. Evaluation of fenugreek (Trigonella foenum-graceum L.), effects seeds extract on insulin resistance in women with polycystic ovarian syndrome. *Iran. J. Pharm. Res.* **2013**, *12*, 475-481.
236. Pourteymour Fard Tabrizi, F.; Abbasalizad Farhangi, M.; Vaezi, M.; Hemmati, S. Changes of body composition and circulating neopterin, omentin-1, and chemerin in response to thylakoid-rich spinach extract with a hypocaloric diet in obese women with polycystic ovary syndrome: A randomized controlled trial. *Phytother. Res.* **2020**, doi:10.1002/ptr.6999.
237. Tabrizi, F.P.F.; Farhangi, M.A.; Vaezi, M.; Hemmati, S. The effects of spinach-derived thylakoid supplementation in combination with calorie restriction on anthropometric parameters and metabolic profiles in obese women with polycystic ovary syndrome: a randomized, double-blind, placebo-controlled clinical trial. *Nutr. J.* **2020**, *19*, 82, doi:10.1186/s12937-020-00601-4.
238. Battaglia, C.; Mancini, F.; Battaglia, B.; Facchinetti, F.; Artini, P.G.; Venturoli, S. L-arginine plus drospirenone-ethinyl estradiol in the treatment of patients with PCOS: a prospective, placebo controlled, randomised, pilot study. *Gynecol. Endocrinol.* **2010**, *26*, 861-868, doi:10.3109/09513590.2010.501882.
239. Chen, J.; Tominaga, K.; Sato, Y.; Anzai, H.; Matsuoka, R. Maitake mushroom (Grifola frondosa) extract induces ovulation in patients with polycystic ovary syndrome: a possible monotherapy and a combination therapy after failure with first-line clomiphene citrate. *J. Altern. Complement. Med.* **2010**, *16*, 1295-1299, doi:10.1089/acm.2009.0696.

240. Shayan, A.; Masoumi, S.Z.; Shobeiri, F.; Tohidi, S.; Khalili, A. Comparing the effects of agnugol and metformin on oligomenorrhea in patients with polycystic ovary syndrome: a randomized clinical trial. *J. Clin. Diagn. Res.* **2016**, *10*, QC13-QC16, doi:10.7860/JCDR/2016/22584.9040.
241. Shahnazi, M.; Khalili, A.F.; Hamdi, K.; Ghahremaninasab, P. The effects of combined low-dose oral contraceptives and Vitex agnus on the improvement of clinical and paraclinical parameters of polycystic ovarian syndrome: a triple-blind, randomized, controlled clinical trial. *Iran. Red Crescent Med. J.* **2016**, *18*, doi:10.5812/ircmj.37510.
242. Armanini, D.; Castello, R.; Scaroni, C.; Bonanni, G.; Faccini, G.; Pellati, D.; Bertoldo, A.; Fiore, C.; Moghetti, P. Treatment of polycystic ovary syndrome with spironolactone plus licorice. *Eur. J. Obstet. Gynecol. Reprod. Biol.* **2007**, *131*, 61-67, doi:10.1016/j.ejogrb.2006.10.013.
243. Pourteymour Fard Tabrizi, F.; Alipoor, B.; Ostadrahimi, A.R.; Mehrzad Sadagiani, M. Effect of zinc supplementation on inflammatory markers in women with polycystic ovary syndrome. *Shiraz E Med. J.* **2011**, *12*, 30-38.
244. Tarkesh, F.; Namavar Jahromi, B.; Hejazi, N.; Tabatabaee, H. Beneficial health effects of Menaquinone-7 on body composition, glycemic indices, lipid profile, and endocrine markers in polycystic ovary syndrome patients. *Food Sci. Nutr.* **2020**, *8*, 5612-5621, doi:10.1002/fsn3.1837.
245. Jazani, A.M.; Nazemiyeh, H.; Tansaz, M.; Bazargani, H.S.; Fazljou, S.M.B.; Azgomi, R.N.D.; Hamdi, K. Celery plus anise versus metformin for treatment of oligomenorrhea in polycystic ovary syndrome: a triple-blind, randomized clinical trial. *Iran. Red Crescent Med. J.* **2018**, *20*, doi:10.5812/ircmj.67181.
246. Zadhoush, R.; Alavi-Naeini, A.; Feizi, A.; Naghshineh, E.; Ghazvini, M.R. The effect of garlic (Allium sativum) supplementation on the lipid parameters and blood pressure levels in women with polycystic ovary syndrome: a randomized controlled trial. *Phytother. Res.* **2021**, *35*, 6335-6342, doi:10.1002/ptr.7282.
247. Mousavi, A.; Saedisomeolia, A.; Yekaninejad, M.; Ildarabadi, A.; Meshkani, M.; Vahid-Dastjerdi, M. Effect of green coffee supplementation on androgens level in women with polycystic ovary syndrome: A Randomized Clinical Trial. *Obesity Medicine* **2020**, *20*, doi:10.1016/j.obmed.2020.100298.
248. Cheshmeh, S.; Ghayyem, M.; Khamooshi, F.; Heidarzadeh-Esfahani, N.; Rahmani, N.; Hojati, N.; Mosaieby, E.; Moradi, S.; Pasdar, Y. Green cardamom plus low-calorie diet can decrease the expression of inflammatory genes among obese women with polycystic ovary syndrome: a double-blind randomized clinical trial. *Eat. Weight Disord.* **2021**, doi:10.1007/s40519-021-01223-3.

249. Qorbani, M.; Sanginabadi, M.; Mohajeri-Tehrani, M.R.; Karimi, S.; Gerami, H.; Mahdavi-Gorabi, A.; Shirzad, N.; Samadi, M.; Baygi, F.; Hosseini, S.; et al. The Effect of Oligopin Supplementation on Hormonal and Metabolic Profiles in the Polycystic Ovary Syndrome: a Randomized Controlled Trial. *Front. Endocrinol. (Lausanne)* **2020**, *11*, 590392, doi:10.3389/fendo.2020.590392.
250. Pourbehi, F.; Ayremlou, P.; Mehdizadeh, A.; Zarrin, R. Effect of psyllium supplementation on insulin resistance and lipid profile in non-diabetic women with polycystic ovary syndrome: a randomized placebo-controlled trial. *Int. J. Womens Health Repr. Sci.* **2020**, *8*, 184-191, doi:10.15296/ijwhr.2020.29.
251. Afandak, F.; Aryaeian, N.; Kashanian, M.; Janani, L.; Namizadeh, T.; Karimi, M.Y.; Morvaridi, M. Effect of sumac powder on clinical symptoms, hyperandrogenism, inflammation, blood glucose, lipid profiles in women with polycystic ovary syndrome: A double-blind randomized clinical trial. *Phytother. Res.* **2023**.
252. Laganà, A.S.; Vitagliano, A.; Noventa, M.; Ambrosini, G.; D'Anna, R.; D'Anna, R. Myo-inositol supplementation reduces the amount of gonadotropins and length of ovarian stimulation in women undergoing IVF: a systematic review and meta-analysis of randomized controlled trials. *Arch. Gynecol. Obstet.* **2018**, *298*, 675-684, doi:10.1007/s00404-018-4861-y.
253. Tang, T.; Lord, J.M.; Norman, R.J.; Yasmin, E.; Balen, A.H. Insulin-sensitising drugs (metformin, rosiglitazone, pioglitazone, D-chiro-inositol) for women with polycystic ovary syndrome, oligo amenorrhoea and subfertility. *Cochrane Database Syst. Rev.* **2010**, doi:10.1002/14651858.CD003053.pub4.
254. Morley, L.C.; Tang, T.; Yasmin, E.; Norman, R.J.; Balen, A.H. Insulin-sensitising drugs (metformin, rosiglitazone, pioglitazone, D-chiro-inositol) for women with polycystic ovary syndrome, oligo amenorrhoea and subfertility. *Cochrane Database Syst. Rev.* **2017**.
255. Mendoza, N.; Perez, L.; Simoncini, T.; Genazzani, A. Inositol supplementation in women with polycystic ovary syndrome undergoing intracytoplasmic sperm injection: a systematic review and meta-analysis of randomized controlled trials. *Reprod. Biomed. Online* **2017**, *35*, 529-535, doi:10.1016/j.rbmo.2017.07.005.
256. Facchinetti, F.; Orro, B.; Grandi, G.; Unfer, V. Short-term effects of metformin and myo-inositol in women with polycystic ovarian syndrome (PCOS): a meta-analysis of randomized clinical trials. *Gynecol. Endocrinol.* **2019**, *35*, 198-206, doi:10.1080/09513590.2018.1540578.

257. Pundir, J.; Psaroudakis, D.; Savnur, P.; Bhide, P.; Sabatini, L.; Teede, H.; Coomarasamy, A.; Thangaratinam, S. Inositol treatment of anovulation in women with polycystic ovary syndrome: a meta-analysis of randomised trials. *BJOG* **2018**, *125*, 299-308, doi:10.1111/1471-0528.14754.
258. Galazis, N.; Galazi, M.; Atiomo, W. D-Chiro-inositol and its significance in polycystic ovary syndrome: a systematic review. *Gynecol. Endocrinol.* **2011**, *27*, 256-262, doi:10.3109/09513590.2010.538099.
259. Tang, T.; Lord, J.M.; Norman, R.J.; Yasmin, E.; Balen, A.H. Insulin-sensitising drugs (metformin, rosiglitazone, pioglitazone, D-chiro-inositol) for women with polycystic ovary syndrome, oligo amenorrhoea and subfertility. *Cochrane Database Syst. Rev.* **2009**, doi:10.1002/14651858.CD003053.pub3.
260. Showell, M.G.; Mackenzie-Proctor, R.; Jordan, V.; Hodgson, R.; Farquhar, C. Inositol for subfertile women with polycystic ovary syndrome. *Cochrane Database Syst. Rev.* **2018**, doi:10.1002/14651858.CD012378.pub2.
261. Zarezadeh, M.; Dehghani, A.; Faghfouri, A.H.; Radkhah, N.; Naemi Kermanshahi, M.; Hamedi Kalajahi, F.; Mohammadzadeh Honarvar, N.; Ghoreishi, Z.; Ostadrahimi, A.; Ebrahimi Mamaghani, M. Inositol supplementation and body mass index: A systematic review and meta-analysis of randomized clinical trials. *Obes. Sci. Pract.* **2021**, doi:10.1002/osp4.569.
262. Tang, T.; Lord, J.M.; Norman, R.J.; Yasmin, E.; Balen, A.H. Insulin-sensitising drugs (metformin, rosiglitazone, pioglitazone, D-chiro-inositol) for women with polycystic ovary syndrome, oligo amenorrhoea and subfertility. *Cochrane Database Syst. Rev.* **2012**.
263. Lord, J.; Flight, I.; Norman, R. Insulin-sensitising drugs (metformin, troglitazone, rosiglitazone, pioglitazone, D-chiro-inositol) for polycystic ovary syndrome. *Cochrane Database Syst Rev* **2003**, CD003053-CD003053.
264. Unfer, V.; Nestler, J.E.; Kamenov, Z.A.; Prapas, N.; Facchinetti, F. Effects of Inositol(s) in Women with PCOS: A Systematic Review of Randomized Controlled Trials. *Int. J. Endocrinol.* **2016**, 1-12, doi:10.1155/2016/1849162.
265. Azizi Kutenaei, M.; Hosseini Teshnizi, S.; Ghaemmaghami, P.; Eini, F.; Roozbeh, N. The effects of myo-inositol vs. metformin on the ovarian function in the polycystic ovary syndrome: a systematic review and meta-analysis. *Eur. Rev. Med. Pharmacol. Sci.* **2021**, *25*, 3105-3115, doi:10.26355/eurrev_202104_25565.
266. Zeng, L.; Yang, K. Effectiveness of myoinositol for polycystic ovary syndrome: a systematic review and meta-analysis. *Endocrine* **2018**, *59*, 30-38, doi:10.1007/s12020-017-1442-y.

267. Zhang, J.Q.; Xing, C.; He, B. Short period-administration of myo-inositol and metformin on hormonal and glycolipid profiles in patients with polycystic ovary syndrome: a systematic review and updated meta-analysis of randomized controlled trials. *Eur. Rev. Med. Pharmacol. Sci.* **2022**, *26*, 1792-1802, doi:10.26355/eurrev_202203_28322.
268. Zhao, H.; Xing, C.; Zhang, J.; He, B. Comparative efficacy of oral insulin sensitizers metformin, thiazolidinediones, inositol, and berberine in improving endocrine and metabolic profiles in women with PCOS: a network meta-analysis. *Reproductive health* **2021**, *18*, 171, doi:10.1186/s12978-021-01207-7.
269. Unfer, V.; Carlomagno, G.; Dante, G.; Facchinetti, F. Effects of myo-inositol in women with PCOS: a systematic review of randomized controlled trials. *Gynecol. Endocrinol.* **2012**, *28*, 509-515, doi:10.3109/09513590.2011.650660.
270. Greff, D.; Juhász, A.E.; Váncsa, S.; Váradi, A.; Sipos, Z.; Szinte, J.; Park, S.; Hegyi, P.; Nyirády, P.; Ács, N. Inositol is an effective and safe treatment in polycystic ovary syndrome: a systematic review and meta-analysis of randomized controlled trials. *Reprod. Biol. Endocrinol.* **2023**, *21*, 10.
271. Unanyan, A.; Pivazyan, L.; Krylova, E.; Eskin, A.; Zakaryan, A.; Sarkisova, A.; Ishchenko, A. Effectiveness of inositol, metformin and their combination in women with PCOS undergoing assisted reproduction: systematic review and meta-analysis. *Gynecol. Endocrinol.* **2022**, *38*, 1035-1046.
272. Abboud, M.; Rizk, R.; AlAnouti, F.; Papandreou, D.; Haidar, S.; Mahboub, N. The Health Effects of Vitamin D and Probiotic Co-Supplementation: A Systematic Review of Randomized Controlled Trials. *Nutrients* **2020**, *13*, doi:10.3390/nu13010111.
273. Jia, X.Z.; Wang, Y.M.; Zhang, N.; Guo, L.N.; Zhen, X.L.; Li, H.; Wei, L. Effect of vitamin D on clinical and biochemical parameters in polycystic ovary syndrome women: A meta-analysis. *J. Obstet. Gynaecol. Res.* **2015**, *41*, 1791-1802, doi:10.1111/jog.12793.
274. Shojaeian, Z.; Sadeghi, R.; Latifnejad Roudsari, R. Calcium and vitamin D supplementation effects on metabolic factors, menstrual cycles and follicular responses in women with polycystic ocvary syndrome: A systematic review and meta-analysis. *Caspian J. Intern. Med.* **2019**, *10*, 359-369, doi:10.22088/cjim.10.4.359.
275. Williams, A.; Babu, J.R.; Wadsworth, D.D.; Burnett, D.; Geetha, T. The Effects of Vitamin D on Metabolic Profiles in Women with Polycystic Ovary Syndrome: A Systematic Review. *Horm. Metab. Res.* **2020**, *52*, 485-491, doi:10.1055/a-1160-9902.
276. Trummer, C.; Pilz, S.; Schwetz, V.; Obermayer-Pietsch, B.; Lerchbaum, E. Vitamin D, PCOS and androgens in men: a systematic review. *Endocr. Connect.* **2018**, *7*, R95-R113, doi:10.1530/ec-18-0009.

277. Guo, S.; Tal, R.; Jiang, H.; Yuan, T.; Liu, Y. Vitamin D Supplementation Ameliorates Metabolic Dysfunction in Patients with PCOS: A SystematicReview of RCTs and Insight into the Underlying Mechanism. *Int. J. Endocrinol.* **2020**, *2020*, 7850816, doi:10.1155/2020/7850816.
278. Xue, Y.; Xu, P.; Xue, K.; Duan, X.; Cao, J.; Luan, T.; Li, Q.; Gu, L. Effect of vitamin D on biochemical parameters in polycystic ovary syndrome women: a meta-analysis. *Arch. Gynecol. Obstet.* **2017**, *295*, 487-496, doi:10.1007/s00404-016-4247-y.
279. Pergialiotis, V.; Karampetsou, N.; Panagopoulos, P.; Trakakis, E.; Papantoniou, N. The effect of Vitamin D supplementation on hormonal and glycaemic profile of patients with PCOS: A meta-analysis of randomised trials. *Int. J. Clin. Pract.* **2017**, *71*, n/a-N.PAG, doi:10.1111/ijcp.12957.
280. Miao, C.-Y.; Fang, X.-J.; Chen, Y.; Zhang, Q. Effect of vitamin D supplementation on polycystic ovary syndrome: A meta-analysis. *Exp. Ther. Med.* **2020**, *19*, 2641-2649, doi:10.3892/etm.2020.8525.
281. Łagowska, K.; Bajerska, J.; Jamka, M. The Role of Vitamin D Oral Supplementation in Insulin Resistance in Women with Polycystic Ovary Syndrome: A Systematic Review and Meta-Analysis of Randomized Controlled Trials. *Nutrients* **2018**, *10*, 1637, doi:10.3390/nu10111637.
282. Luo, J.; Li, T.; Yuan, J. Effectiveness of vitamin D supplementation on lipid profile in polycystic ovary syndrome women: a meta-analysis of randomized controlled trials. *Ann. Palliat. Med.* **2021**, *10*, 114-129, doi:10.21037/apm-20-2492.
283. Wang, L.; Wen, X.; Lv, S.; Tian, S.; Jiang, Y.; Yang, X. Effects of vitamin D supplementation on metabolic parameters of women with polycystic ovary syndrome: a meta-analysis of randomized controlled trials. *Gynecol. Endocrinol.* **2021**, *37*, 446-455, doi:10.1080/09513590.2020.1813272.
284. Zhao, J.-F.; Li, B.-X.; Zhang, Q. Vitamin D improves levels of hormonal, oxidative stress and inflammatory parameters in polycystic ovary syndrome: a meta-analysis study. *Ann. Palliat. Med.* **2021**, *10*, 169-183, doi:10.21037/apm-20-2201.
285. Azadi-Yazdi, M.; Nadjarzadeh, A.; Khosravi-Boroujeni, H.; Salehi-Abargouei, A. The Effect of Vitamin D Supplementation on the Androgenic Profile in Patients with Polycystic Ovary Syndrome: A Systematic Review and Meta-Analysis of Clinical Trials. *Horm. Metab. Res.* **2017**, *49*, 174-179, doi:10.1055/s-0043-103573.
286. Han, Y.; Cao, Q.; Qiao, X.; Huang, W. Effect of vitamin D supplementation on hormones and menstrual cycle regularization in polycystic ovary syndrome women: A systemic review and meta-analysis. *J. Obstet. Gynaecol. Res.* **2023**.

287. Yang, M.; Shen, X.; Lu, D.; Peng, J.; Zhou, S.; Xu, L.; Zhang, J. Effects of vitamin D supplementation on ovulation and pregnancy in women with polycystic ovary syndrome: a systematic review and meta-analysis. *Front. Endocrinol. (Lausanne)* **2023**, *14*.
288. Devi, N.; Boya, C.; Chhabra, M.; Bansal, D. N-acetyl-cysteine as adjuvant therapy in female infertility: a systematic review and meta-analysis. *J. Basic Clin. Physiol. Pharmacol.* **2020**, *32*, 899-910, doi:10.1515/jbcpp-2020-0107.
289. Thakker, D.; Raval, A.; Patel, I.; Walia, R. N-Acetylcysteine for Polycystic Ovary Syndrome: A Systematic Review and Meta-Analysis of Randomized Controlled Clinical Trials. *Obstet. Gynecol. Int.* **2015**, *2015*, 1-13, doi:10.1155/2015/817849.
290. Song, Y.; Wang, H.; Huang, H.; Zhu, Z. Comparison of the efficacy between NAC and metformin in treating PCOS patients: a meta-analysis. *Gynecol. Endocrinol.* **2020**, *36*, 204-210, doi:10.1080/09513590.2019.1689553.
291. Liu, J.; Su, H.; Jin, X.; Wang, L.; Huang, J. The effects of N-acetylcysteine supplement on metabolic parameters in women with polycystic ovary syndrome: a systematic review and meta-analysis. *Front. Nutr.* **2023**, *10*.
292. Asl, Z.S.; Parastouei, K.; Eskandari, E. The effects of N-acetylcysteine on ovulation and sex hormones profile in women with polycystic ovary syndrome: A systematic review and meta-analysis. *Br. J. Nutr.* **2023**, 1-28.
293. Yang, K.; Zeng, L.; Bao, T.; Ge, J. Effectiveness of Omega-3 fatty acid for polycystic ovary syndrome: a systematic review and meta-analysis. *Reprod. Biol. Endocrinol.* **2018**, *16*, 27, doi:10.1186/s12958-018-0346-x.
294. Xia, Y.; Wang, Y.; Cui, M.; Su, D. Efficacy of omega-3 fatty acid supplementation on cardiovascular risk factors in patients with polycystic ovary syndrome: a systematic review and meta-analysis. *Ann. Palliat. Med.* **2021**, *10*, 6425-6437, doi:10.21037/apm-21-1050.
295. Melo, V.; Silva, T.; Silva, T.; Freitas, J.; Sacramento, J.; Vazquez, M.; Araujo, E. Omega-3 supplementation in the treatment of polycystic ovary syndrome (PCOS) - a review of clinical trials and cohort. *Endocr. Regul.* **2022**, *56*, 66-79, doi:10.2478/enr-2022-0008.
296. Yuan, J.; Wen, X.; Jia, M. Efficacy of omega-3 polyunsaturated fatty acids on hormones, oxidative stress, and inflammatory parameters among polycystic ovary syndrome: a systematic review and meta-analysis. *Ann. Palliat. Med.* **2021**, *10*, 8991-9001, doi:10.21037/apm-21-2018.
297. Sadeghi, A.; Djafarian, K.; Mohammadi, H.; Shab-Bidar, S. Effect of omega-3 fatty acids supplementation on insulin resistance in women with polycystic ovary syndrome: Meta-analysis of randomized controlled trials. *Diabetes Metab. Syndr.* **2017**, *11*, 157-162, doi:10.1016/j.dsx.2016.06.025.

298. Zhou, J.; Zuo, W.; Tan, Y.; Wang, X.; Zhu, M.; Zhang, H. Effects of n-3 polyunsaturated fatty acid on metabolic status in women with polycystic ovary syndrome: a meta-analysis of randomized controlled trials. *J. Ovarian Res.* **2023**, *16*, 1-21.
299. Hajishafiee, M.; Askari, G.; Iranj, B.; Ghiasvand, R.; Bellissimo, N.; Totosy de Zepetnek, J.; Salehi-Abargouei, A. The Effect of n-3 Polyunsaturated Fatty Acid Supplementation on Androgen Status in Patients with Polycystic Ovary Syndrome: A Systematic Review and Meta-Analysis of Clinical Trials. *Horm. Metab. Res.* **2016**, *48*, 281-289, doi:10.1055/s-0042-105288.
300. Di Tucci, C.; Galati, G.; Mattei, G.; Bonanni, V.; Capri, O.; D'Amelio, R.; Muzii, L.; Benedetti Panici, P. The role of alpha lipoic acid in female and male infertility: a systematic review. *Gynecol. Endocrinol.* **2021**, *37*, 497-505, doi:10.1080/09513590.2020.1843619.
301. Emamat, H.; Najafpour Boushehri, S.; Eslami, M.A.; Saneei Totmaj, A.; Ghalandari, H. The effects of flaxseed or its oil supplementations on polycystic ovary syndrome: A systematic review of clinical trials. *Phytother. Res.* **2023**, *37*, 1082-1091.
302. Shirvani-Rad, S.; Tabatabaei-Malazy, O.; Mohseni, S.; Hasani-Ranjbar, S.; Soroush, A.-R.; Hoseini-Tavassol, Z.; Ejtahed, H.-S.; Larijani, B. Probiotics as a Complementary Therapy for Management of Obesity: A Systematic Review. *Evid. Based Complement. Alternat. Med.* **2021**, *2021*, 6688450, doi:10.1155/2021/6688450.
303. Shamasbi, S.G.; Ghanbari-Homayi, S.; Mirghafourvand, M. The effect of probiotics, prebiotics, and synbiotics on hormonal and inflammatory indices in women with polycystic ovary syndrome: a systematic review and meta-analysis. *Eur. J. Nutr.* **2020**, *59*, 433-450, doi:10.1007/s00394-019-02033-1.
304. Cozzolino, M.; Vitagliano, A.; Pellegrini, L.; Chiurazzi, M.; Andriasani, A.; Ambrosini, G.; Garrido, N. Therapy with probiotics and synbiotics for polycystic ovarian syndrome: a systematic review and meta-analysis. *Eur. J. Nutr.* **2020**, *59*, 2841-2856, doi:10.1007/s00394-020-02233-0.
305. Heshmati, J.; Farsi, F.; Yosaee, S.; Razavi, M.; Rezaeinejad, M.; Karimie, E.; Sepidarkish, M. The Effects of Probiotics or Synbiotics Supplementation in Women with Polycystic Ovarian Syndrome: a Systematic Review and Meta-Analysis of Randomized Clinical Trials. *Probiotics Antimicrob. Proteins* **2019**, *11*, 1236-1247, doi:10.1007/s12602-018-9493-9.
306. Hadi, A.; Moradi, S.; Ghavami, A.; Khalesi, S.; Kafeshani, M. Effect of probiotics and synbiotics on selected anthropometric and biochemical measures in women with polycystic ovary syndrome: a systematic review and meta-analysis. *Eur. J. Clin. Nutr.* **2020**, *74*, 543-547, doi:10.1038/s41430-019-0434-9.

307. Talebi, S.; Zeraattalab-Motlagh, S.; Jalilpiran, Y.; Payandeh, N.; Ansari, S.; Mohammadi, H.; Djafarian, K.; Ranjbar, M.; Sadeghi, S.; Taghizadeh, M. The effects of pro-, pre-, and synbiotics supplementation on polycystic ovary syndrome: an umbrella review of meta-analyses of randomized controlled trials. *Front. Nutr.* **2023**, *10*.
308. Angoorani, P.; Ejtahed, H.-S.; Ettehad Marvasti, F.; Taghavi, M.; Mohammadpour Ahranjani, B.; Hasani-Ranjbar, S.; Larijani, B. The effects of probiotics, prebiotics, and synbiotics on polycystic ovarian syndrome: an overview of systematic reviews. *Front. Med.* **2023**, *10*, 1141355.
309. Heydarpour, F.; Hemati, N.; Hadi, A.; Moradi, S.; Mohammadi, E.; Farzaei, M.H. Effects of cinnamon on controlling metabolic parameters of polycystic ovary syndrome: A systematic review and meta-analysis. *J. Ethnopharmacol.* **2020**, *254*, N.PAG-N.PAG, doi:10.1016/j.jep.2020.112741.
310. Heshmati, J.; Sepidarkish, M.; Morvaridzadeh, M.; Farsi, F.; Tripathi, N.; Razavi, M.; Rezaeinejad, M. The effect of cinnamon supplementation on glycemic control in women with polycystic ovary syndrome: A systematic review and meta-analysis. *J. Food Biochem.* **2021**, *45*, e13543, doi:10.1111/jfbc.13543.
311. Keramati, M.; Musazadeh, V.; Malekahmadi, M.; Jamilian, P.; Jamilian, P.; Ghoreishi, Z.; Zarezadeh, M.; Ostadrahimi, A. Cinnamon, an effective anti-obesity agent: Evidence from an umbrella meta-analysis. *J. Food Biochem.* **2022**, e14166, doi:10.1111/jfbc.14166.
312. Maleki, V.; Faghfouri, A.H.; Tabrizi, F.P.F.; Moludi, J.; Saleh-Ghadimi, S.; Jafari-Vayghan, H.; Qaisar, S.A. Mechanistic and therapeutic insight into the effects of cinnamon in polycystic ovary syndrome: a systematic review. *J. Ovarian Res.* **2021**, *14*, 130, doi:10.1186/s13048-021-00870-5.
313. Sarmadi, B.; Musazadeh, V.; Dehghan, P.; Karimi, E. The Effect of Cinnamon Consumption on Lipid Profile, Oxidative Stress, and Inflammation Biomarkers in adults: An umbrella meta-analysis of randomized controlled trials. *Nutr. Metab. Cardiovasc. Dis.* **2023**.
314. Ahmad, F.; Safuan, S. Assessing the effectiveness of plant extracts in polycystic ovarian syndrome: A systematic review. *Malays. J. Med. Health Sci.* **2019**, *15*, 120-129.
315. Moini Jazani, A.; Nasimi Doost Azgomi, H.; Nasimi Doost Azgomi, A.; Nasimi Doost Azgomi, R. A comprehensive review of clinical studies with herbal medicine on polycystic ovary syndrome (PCOS). *DARU* **2019**, *27*, 863-877, doi:10.1007/s40199-019-00312-0.

316. Pundir, J.; Charles, D.; Sabatini, L.; Hiam, D.; Jitpiriyaroj, S.; Teede, H.; Coomarasamy, A.; Moran, L.; Thangaratinam, S. Overview of systematic reviews of non-pharmacological interventions in women with polycystic ovary syndrome. *Hum. Reprod. Update* **2019**, *25*, 243-256, doi:10.1093/humupd/dmy045.
317. Mohseni, S.; Tabatabaei-Malazy, O.; Shadman, Z.; Khashayar, P.; Mohajeri-Tehrani, M.; Larijani, B. Targeting dyslipidemia with antioxidative vitamins C, D, and E; a systematic review of meta-analysis studies: Dyslipidemia and antioxidative vitamins. *J. Diabetes Metab. Disord.* **2021**, *20*, 2037-2047, doi:10.1007/s40200-021-00919-8.
318. Showell, M.G.; Mackenzie-Proctor, R.; Jordan, V.; Hart, R.J. Antioxidants for female subfertility. *Cochrane Database Syst. Rev.* **2020**, doi:10.1002/14651858.CD007807.pub4.
319. Arentz, S.; Smith, C.A.; Abbott, J.; Bensoussan, A. Nutritional supplements and herbal medicines for women with polycystic ovary syndrome; a systematic review and meta-analysis. *BMC Complement. Altern. Med.* **2017**, *17*, 1-14.
320. Zhang, J.; Xing, C.; Zhao, H.; He, B. The effectiveness of coenzyme Q10, vitamin E, inositols, and vitamin D in improving the endocrine and metabolic profiles in women with polycystic ovary syndrome: a network Meta-analysis. *Gynecol. Endocrinol.* **2021**, *37*, 1063-1071, doi:10.1080/09513590.2021.1926975.
321. Florou, P.; Anagnostis, P.; Theocharis, P.; Chourdakis, M.; Goulis, D.G. Does coenzyme Q10 supplementation improve fertility outcomes in women undergoing assisted reproductive technology procedures? A systematic review and meta-analysis of randomized-controlled trials. *J. Assist. Reprod. Genet.* **2020**, *37*, 2377-2387, doi:10.1007/s10815-020-01906-3.
322. Zhang, T.; He, Q.; Xiu, H.; Zhang, Z.; Liu, Y.; Chen, Z.; Hu, H. Efficacy and safety of Coenzyme Q10 supplementation in the treatment of Polycystic Ovary Syndrome: A systematic review and meta-analysis. *Reprod. Sci.* **2023**, *30*, 1033-1048.
323. Abdelazeem, B.; Abbas, K.S.; Shehata, J.; Baral, N.; Banour, S.; Hassan, M. The effects of curcumin as dietary supplement for patients with polycystic ovary syndrome: An updated systematic review and meta-analysis of randomized clinical trials. *Phytother. Res.* **2022**, *36*, 22-32, doi:10.1002/ptr.7274.
324. Shojaei-Zarghani, S.; Molani-Gol, R.; Rafraf, M. Curcumin and Polycystic Ovary Syndrome: a Systematic Review. *Reprod. Sci.* **2022**, doi:10.1007/s43032-021-00826-6.
325. Chien, Y.-J.; Chang, C.-Y.; Wu, M.-Y.; Chen, C.-H.; Horng, Y.-S.; Wu, H.-C.; Ciarmela, P. Effects of Curcumin on Glycemic Control and Lipid Profile in Polycystic Ovary Syndrome: Systematic Review with Meta-Analysis and Trial Sequential Analysis. *Nutrients* **2021**, *13*, 684-684, doi:10.3390/nu13020684.

326. Venkatesan, K.; Menon, S.; Firoz, I.N.; Narendrula, R.; Kunnath, J.T.; Kalapurakal, A.M.; Haroon, N.; Iqbal, A.M.; Ashok, A. Complementary administration of curcumin in PCOS: a systematic review. *Minerva Obstet. Gynecol.* **2022**.
327. Liao, D.; Liu, X.; Yuan, X.; Feng, P.; Ouyang, Z.; Liu, Y.; Li, C. Clinical evidence of the effects of carnitine supplementation on body weight, glycemic control and serum lipids in women with polycystic ovary syndrome: a systematic review and meta-analysis. *Gynecol. Endocrinol.* **2022**, *38*, 110-115, doi:10.1080/09513590.2021.1988559.
328. Gong, Y.; Jiang, T.; He, H.; Wang, Y.; Wu, G.L.; Shi, Y.; Cai, Q.; Xiong, C.L.; Shen, R.; Li, J. Effects of carnitine on glucose and lipid metabolic profiles and fertility outcomes in women with polycystic ovary syndrome: A systematic review and meta-analysis. *Clin. Endocrinol. (Oxf.)* **2023**, *98*, 682-691.
329. Tang, X.L.; Sun, Z.; Gong, L. Chromium supplementation in women with polycystic ovary syndrome: Systematic review and meta-analysis. *J. Obstet. Gynaecol. Res.* **2018**, *44*, 134-143, doi:10.1111/jog.13462.
330. Maleki, V.; Izadi, A.; Farsad-Naeimi, A.; Alizadeh, M. Chromium supplementation does not improve weight loss or metabolic and hormonal variables in patients with polycystic ovary syndrome: A systematic review. *Nutr. Res.* **2018**, *56*, 1-10, doi:10.1016/j.nutres.2018.04.003.
331. Fazelian, S.; Rouhani, M.H.; Bank, S.S.; Amani, R. Chromium supplementation and polycystic ovary syndrome: A systematic review and meta-analysis. *J. Trace Elem. Med. Biol.* **2017**, *42*, 92-96, doi:10.1016/j.jtemb.2017.04.008.
332. Shojaei-Zarghani, S.; Rafraf, M. Resveratrol and Markers of Polycystic Ovary Syndrome: a Systematic Review of Animal and Clinical Studies. *Reprod. Sci.* **2021**, doi:10.1007/s43032-021-00653-9.
333. Xie, L.; Zhang, D.; Ma, H.; He, H.; Xia, Q.; Shen, W.; Chang, H.; Deng, Y.; Wu, Q.; Cong, J.; et al. The Effect of Berberine on Reproduction and Metabolism in Women with Polycystic Ovary Syndrome: A Systematic Review and Meta-Analysis of Randomized Control Trials. *Evid. Based Complement. Alternat. Med.* **2019**, *2019*, 7918631-7918631, doi:10.1155/2019/7918631.
334. Mirzaee, F.; Razmjouei, P.; Shahrahmani, H.; Vafisani, F.; Najaf Najafi, M.; Ghazanfarpour, M. The effect and safety of Berberine on polycystic ovary syndrome: a systematic review. *J. Obstet. Gynaecol.* **2021**, *41*, 684-689, doi:10.1080/01443615.2020.1787964.
335. Hajizadeh-Sharafabad, F.; Moludi, J.; Tutunchi, H.; Taheri, E.; Izadi, A.; Maleki, V. Selenium and Polycystic Ovary Syndrome; Current Knowledge and Future Directions: A Systematic Review. *Horm. Metab. Res.* **2019**, *51*, 279-287, doi:10.1055/a-0890-6823.

336. Zhao, J.; Dong, L.; Lin, Z.; Sui, X.; Wang, Y.; Li, L.; Liu, T.; Liu, J. Effects of selenium supplementation on Polycystic Ovarian Syndrome: a systematic review and meta-analysis on randomized clinical trials. *BMC Endocr. Disord.* **2023**, *23*, 33.
337. Kazempour, R.; Abedi, P.; Siahkal, S.F.; Sharifipour, F.; Zahedian, M. Effect of Selenium Supplementation on Biochemical Markers of Women with Polycystic Ovarian Syndrome: A Systematic Review. *Prev. Nutr. Food Sci.* **2023**, *28*, 121.
338. Wu, P.-y.; Tan, X.; Wang, M.; Zheng, X.; Lou, J.-h. Selenium supplementation for polycystic ovary syndrome: a meta-analysis of randomized controlled trials. *Gynecol. Endocrinol.* **2022**, *38*, 928-934.
339. Hu, K.-L.; Ye, X.; Wang, S.; Zhang, D. Melatonin Application in Assisted Reproductive Technology: A Systematic Review and Meta-Analysis of Randomized Trials. *Front. Endocrinol. (Lausanne)* **2020**, *11*, 160, doi:10.3389/fendo.2020.00160.
340. Shen, W.; Pan, Y.; Jin, B.; Zhang, Z.; You, T.; Qu, Y.; Han, M.; Yuan, X.; Zhang, Y. Effects of Tea Consumption on Anthropometric Parameters, Metabolic Indexes and Hormone Levels of Women with Polycystic Ovarian Syndrome: A Systematic Review and Meta-Analysis of Randomized Controlled Trials. *Front. Endocrinol. (Lausanne)* **2021**, *12*, 736867, doi:10.3389/fendo.2021.736867.
341. Pourteymour Fard Tabrizi, F.; Hajizadeh-Sharafabad, F.; Vaezi, M.; Jafari-Vayghan, H.; Alizadeh, M.; Maleki, V. Quercetin and polycystic ovary syndrome, current evidence and future directions: a systematic review. *J. Ovarian Res.* **2020**, *13*, 11, doi:10.1186/s13048-020-0616-z.
342. Heidari, H.; Hajhashemy, Z.; Saneei, P. A meta-analysis of effects of vitamin E supplementation alone and in combination with omega-3 or magnesium on polycystic ovary syndrome. *Sci. Rep.* **2022**, *12*, 19927.
343. Jafari, A.; Gholizadeh, E.; Sadrmanesh, O.; Tajpour, S.; Yarizadeh, H.; Zamani, B.; Sohrabi, Z. The effect of folic acid supplementation on body weight and body mass index: a systematic review and meta-analysis of randomized controlled trials. *Clin. Nutr. ESPEN* **2022**.
344. Najafi, M.N.; Kasaian, J.; Kovatsi, L.; Leon, G.; Solout, E.K.; Hashemzaei, M.; Rezaee, R.; Modiramani, P.; Ghazanfarpour, M. Phytoestrogens and the polycystic ovary syndrome: A systematic review of clinical evidence and laboratory findings. *Farmacia* **2018**, *66*, 223-229.
